# Linking Structure to Electrocatalytic Performance: Graphene Nanoplatelets-Derived Novel Mixed Oxide–Carbon Composites as Supports for Pt Electrocatalysts with Enhanced Stability

**DOI:** 10.3390/nano15231753

**Published:** 2025-11-22

**Authors:** Ilgar Ayyubov, Emília Tálas, Irina Borbáth, Zoltán Pászti, László Trif, Ágnes Szegedi, Catia Cannilla, Giuseppe Bonura, Tamás Szabó, Erzsébet Dodony, András Tompos

**Affiliations:** 1 Institute of Materials and Environmental Chemistry, HUN-REN Research Centre for Natural Sciences, Magyar Tudósok körútja 2, H-1117 Budapest, Hungary; ilgar.ayyubov@ttk.hu (I.A.); talas.emilia@ttk.hu (E.T.); borbath.irina@ttk.hu (I.B.); trif.laszlo@ttk.hu (L.T.); szegedi.agnes@ttk.hu (Á.S.); 2 Institute of Advanced Technology for Energy CNR-ITAE, S. Lucia Sopra Contesse 5, 98126 Messina, Italy; catia.cannilla@cnr.it (C.C.); giuseppe.bonura@cnr.it (G.B.); 3 Department of Physical Chemistry and Materials Science, University of Szeged, Rerrich Béla tér 1, H-6720 Szeged, Hungary; sztamas@chem.u-szeged.hu; 4 Institute for Technical Physics and Materials Science, HUN-REN Centre for Energy Research, Konkoly-Thege M. út 29-33, H-1121 Budapest, Hungary; dodony.erzsebet@ek.hun-ren.hu

**Keywords:** electrocatalyst, mixed oxide-carbon composites, sol–gel method, graphene nanoplatelets (GNPs), GNPs-graphite oxide (GO) mixture, XRD, TG-MS, TEM, SEM, XPS

## Abstract

The lifetime of polymer electrolyte membrane fuel cells (PEMFCs) is significantly influenced by the degradation of their catalysts. A composite-type electrocatalyst support with the formula Ti_(1−x)_Mo_x_O_2_-C (x: 0–0.2, C: carbon) has been found to provide higher stability for the Pt active metal than carbon alone. Non-traditional carbon materials such as graphene nanoplatelets (GNPs) and graphite oxide (GO) offer new possibilities for supports. This work aims to explore whether it is possible to combine the advantageous properties of GNP and GO in composite-supported Pt electrocatalysts. Composites prepared using the modified sol–gel method and Pt catalysts supported on them were characterized by physicochemical methods. Electrochemical behavior in terms of CO tolerance, activity and stability was studied. Although GO transformed into a mainly graphitic material during composite synthesis, its addition still increased the functional group content of the carbonaceous backbone. The electrical conductivity was significantly higher when GNPs-GO mixtures were used as the starting carbon material compared to the use of pure GNPs. Increased CO oxidation activity was achieved due to the incorporated Mo. Stability of the composite-supported Pt catalyst was significantly higher than that of commercial Pt/C. Increased stability of the GNPs-GO-derived catalyst compared to the GNP-derived one was obtained.

## 1. Introduction

Polymer electrolyte membrane fuel cells (PEMFCs) convert chemical energy into electricity efficiently and in an environmentally friendly way, and their use provides advantages in a wide variety of areas [1,2,3,4]. The lifetime of PEMFCs is significantly influenced by the degradation of the catalyst used in them [5,6,7]. Due to its activity both in the oxygen reduction reaction (ORR) on the cathode and in the hydrogen oxidation reaction (HOR) on the anode side, nano-dispersed platinum is a favorable electrocatalyst [6,8]. Likewise, due to its excellent conductivity, carbon is a suitable catalyst support. As a consequence, carbon-supported platinum (Pt/C) is a widely used electrocatalyst for commercial PEMFC; however, Pt/C is prone to electrocorrosion [9,10,11] and is not resistant to CO poisoning [8,12]. One of the reasons behind electrocorrosion is that carbon is not thermodynamically stable under the operating conditions in PEMFCs [13]. The effect of catalyst degradation is offset by applying high Pt loading to the electrodes, which improves the lifetime of the PEMFC to an acceptable level. Since Pt is one of the critical and strategic raw materials [14,15,16], the development of Pt electrocatalysts with increased stability contributing to lower Pt need has great importance.

In our previous works, we found that composite-type catalyst supports with the formula Ti_(1−x)_Mo_x_O_2_-C (x: 0–0.2, C: Black Pearls 2000, Vulcan XC-72 (Cabot)) provided higher stability for the Pt active metal than simple conductive carbon supports [17]. Every element of the above composite has an important function: the TiO_2_ skeleton being strongly resistant to electrocorrosion increases the stability and hinders the dissolution of the incorporated dopant; the Mo doping ions act as a co-catalyst providing enhanced CO oxidation activity and CO tolerance; and the carbon material provides appropriate specific surface area (SSA_BET_) and electrical conductivity [17,18]. We have already conducted some preliminary studies to produce this type of catalyst support from non-traditional carbon materials such as graphite oxide (GO) [17,18] and graphene nanoplatelets (GNPs) [18].

Graphene-based, non-traditional carbon materials such as carbon nanotubes (CNT) and graphene nanosheets [19,20,21,22,23,24,25,26,27] offer new possibilities for electrocatalyst support, but certain functionalization is required for their use [28,29]. The functionalization of the carbonaceous materials is especially important when preparing their composites with inorganic materials [30,31]. The method of modification strongly depends on the type of the graphene materials, since the family of graphene derivatives is rather extended [32]. They are classified both according to their functional groups and their size range [32,33]. GO is known for a long time [34,35] and is now popularly used as a starting material for various syntheses [23,36]. Delaminated GO, which is actually graphene oxide, can be considered “as derivatized graphene with a myriad of oxygen functionalities due to the introduction of carbonyl, hydroxyl, and epoxy groups on the planar surfaces and edges of the carbon sheets” [33]. The specific structure of GO results in its thermal instability [37], weak acidity in water and more hydrophilic character [38]. Its amphiphilic character makes it suitable for easy use in aqueous syntheses [36]. Nevertheless, non-oxidized aromatic regions and/or isolated C = C double bonds are also characteristic of the structure of GO [37]. However, GO is relatively expensive, and harmful waste materials are formed during its production. In contrast, the other commonly used “graphene” material, GNP, is relatively cheap and commercially available in large quantities [39,40,41]. There is increasing interest in the use of GNP as an electrocatalyst support [42,43,44,45,46]. Due to its relatively extensive condensed aromatic ring system, the thermal and electrical conductivity of GNP is excellent [47]. However, GNPs are much less hydrophilic than GO, and the amount of O-containing functional groups on them is insufficient, as they are positioned only on the edges of the nanoparticles [48]. The latter is not particularly advantageous for preparing composites in which cohesive forces are based on covalent bonds, such as composites of inorganic components synthesized via the sol–gel method.

Nevertheless, other interactions besides covalent bonds may also play a decisive role in carbonaceous–carbonaceous composites. The π-π* interaction is supposed to be responsible for the formation of graphene–CNT hybrid materials [49] and composites of CNT- hyperbranched polyesters with terminal functional groups [50]. π-π bond-based, so-called non-covalent functionalization was used to create an N-type anchoring site for Pt by the introduction of electron-conducting polyaniline (PANI) onto the surface of single wall nanotubes (SWCNTs) [51]. Non-covalent functionalization with amphiphilic polymers resulted in very well-dispersed multi-walled carbon nanotubes (MWNTs) in a solvent of dimethylformamide, water and chloroform [52]. In another work, phenyl glycidyl ether molecules were loaded onto MWCNTs based on π-π* interaction, which increased the dispersibility of MWCNTs in polyamide nanocomposite fibers [53]. A hexaazatrinaphthalene-conductive reduced graphene oxide (rGO) composite for aqueous proton storage was successfully prepared, in which π-π interaction existed [54]. GO has been described to serve as a superior dispersant to disperse pristine CNTs [55] or GNPs [48,56] into water to form stable suspensions. These observations can be attributed to interactions between the carbonaceous sheets of the different graphene derivatives, including π-π attraction [48,56], as well as to the hydrophilic nature of GO, due to its O-containing functional groups.

This work aimed to explore whether it is possible to combine the advantageous properties of GNPs and GO in the catalyst production method we developed. Therefore, we studied the effect of mixtures of GNPs and GO on the behavior of the mixed oxide–carbon composite-supported electrocatalyst. A further question is whether the presence of GO during the preparation of the composite results in a more stable catalyst than pure GNPs. Finally, could the properties of the new catalysts indicate a GO-GNP interaction; can we assume non-covalent functionalization of GNPs by GO? In order to investigate the utilization of advanced carbon materials, a series of Ti_(1−x)_Mo_x_O_2_-C composite-type supports and supported Pt electrocatalysts were prepared using GNPs or GO-GNP mixtures (3:1, 1:1, 1:3) as starting carbon-containing materials. To the best of our knowledge, preparation of a mixed oxide–carbon composite-type electrocatalyst support from GNP-GO carbonaceous components, as well as the changes in the carbonaceous part during the composite formation, are reported here for the first time in the literature. The new types of support materials and the catalysts prepared by loading them with 20 wt.% Pt are compared based on their physicochemical properties and electrochemical behavior.

## 2. Materials and Methods

### 2.1. Materials

Three different commercial GNPs (Nanografi, Ankara, Turkey, specific surface area: 700 m^2^/g; Sigma-Aldrich No900394 (Sigma-Aldrich, St. Louis, MO, USA), average surface area: 300 m^2^/g; Sigma-Aldrich No900407 (Sigma-Aldrich, St. Louis, MO, USA), average surface area: 750 m^2^/g) and homemade GO were used as carbon precursors for composite preparation. GO suspension (0.95 wt.% for carbon) was prepared by the Hummers–Offeman technique [35]. The Ti and Mo precursor compounds were titanium-isopropoxide (Ti(O-*i*-Pr)_4_, Sigma-Aldrich, St. Louis, MO, USA, 97%) and ammonium heptamolybdate tetrahydrate ((NH_4_)_6_Mo_7_O_24_ × 4H_2_O, Merck, Darmstadt, Germany, 99%). The Pt precursor was hexachloroplatinic acid hexahydrate (H_2_PtCl_6_ × 6H_2_O, Sigma-Aldrich, St. Louis, MO, USA, 37.5% Pt), and 20 wt.% Pt/Vulcan XC-72 (Quintech, Göppingen, Germany) was used as reference Pt/C electrocatalyst. Nitric acid (HNO_3_, 65%, a.r.), 2-propanol (*i*-C_3_H_5_OH, 99.9 *V*/*V*%, a.r.) ethylene-glycol (EG, 99.8%) and sodium borohydride (NaBH_4_, 99.95%) were purchased from Molar Chemicals, Halásztelek, Hungary. Sulfuric acid (H_2_SO_4_, 96% p.a., Merck, Darmstadt, Germany) and hydrochloric acid (HCl, 36,4%, AnalaR NORMAPUR, VWR International, Fontenay-sous-Bois, France) were also used. Sodium hydroxide (NaOH, >98%) and hydrofluoric acid (38–41%, a.r.) were Reanal (Budapest, Hungary) products. The gases (Ar, N_2_, H_2_) used in this work were products of Linde Gáz Magyarország Zrt. (Budapest, Hungary) with 5.0 purity. Catalyst ink was prepared using a 5% Nafion^®^ dispersion (DuPont™ Nafion^®^ PFSA Polymer Dispersions DE 520, DuPont, Wilmington, DE, USA).

### 2.2. Preparation of Composite Supports and Supported Pt Electrocatalysts

Denomination of the composite materials used as electrocatalyst supports is shown in Table 1. Synthesis of the composites started with preparation of the mixture of carbonaceous components either from GNPs and water or from GO sol, GNPs and water. It is important to mention that due to the highly apolar nature of GNPs, intensive sonication by a Hielscher UP200S ultrasonic device (Hielscher Ultrasonics GmbH, Teltow, Germany) was used. In the case of the GO component, the pH of the mixtures was adjusted to 9 with concentrated NaOH solution to reach more intense exfoliation. In parallel, we started the formation of TiO_2_ nuclei from Ti(O-*i*-Pr)_4_ in acidic sol (5 h), and then the two mixtures were combined according to the sol–gel based multi-step synthesis method optimized in our previous works [17,57]. This procedure involves three separate steps: (i) formation and aging of TiO_2_ nuclei on the carbonaceous material (6 days), (ii) addition of Mo-precursor and drying at low temperature (overnight at 85 °C), and (iii) high-temperature heat treatment (HTT) in Ar at 600 °C for the crystallization of mixed oxide and Mo incorporation.

Details of the present composite preparation method can be found in the Supplementary Materials (Section S1.1 and Figures S1 and S2). In this work we aimed to prepare the mixed oxide part of the new composites from GNPs and from GNP-GO mixtures with the same nominal compositions as we used previously; therefore, the oxide/carbon mass ratio of 75/25 and Ti/Mo atomic ratio of 80/20 were chosen [17,57]. We kept the Ti/Mo ratio relatively low, as we previously found that at doping levels above 20–30%, the Mo dopant incorporated incompletely into the mixed oxide, and an undesirable, separate MoO_2_ phase appeared. Another prerequisite of the exclusive Mo incorporation into the mixed oxide is the formation of a rutile-TiO_2_ phase [17]. Therefore, composite formation and aging of TiO_2_ nuclei on the carbonaceous material were carried out in diluted HNO_3_ solution.

It should be mentioned that in the case of the GO-containing samples, prior to Mo introduction, a washing step with HNO_3_ was included in order to avoid harmful NaNO_3_ formation [18]. Further synthesis parameters of the composites are summarized in Table 1.

To obtain platinum-containing electrocatalysts, the Ti_(1×x)_Mo_x_O_2_-C supports were loaded with 20 wt.% Pt by a modified, NaBH_4_-assisted EG reduction–precipitation technique, as we described previously [58], demonstrated in Figure S3 (Section S1 of the Supplementary Materials).

### 2.3. Physicochemical Characterization of Composite Supports and Supported Pt Electrocatalysts

Details of the nitrogen physisorption measurements, transmission electron microscopy (TEM) and scanning electron microscopy (SEM) studies, inductively coupled plasma-optical emission spectrometry (ICP-OES), X-ray photoelectron spectroscopy (XPS) and X-ray powder diffraction (XRD) measurements are described in the Supplementary Materials (Section S2.1). The average crystallite sizes of Pt and mixed oxide were determined from XRD data by means of the Scherrer formula [59,60].

The surface morphology of the samples was investigated using an Ultra-High-Resolution Scanning Electron Microscope (UHR-SEM-FEG, Helios 5 UC DualBeam, Thermo Scientific, Waltham, MA, USA) equipped with a Field Emission Gun (FEG), which provides high spatial resolution and improved image quality.

The simultaneous thermogravimetric (TG) and mass spectrometric (MS) evolved gas analyses were recorded on a Setaram LabsysEvo (Setaram, Lyon, France) thermal analyzer, in high purity (99.9999%) He, with a flow rate of 90 cm^3^/min. The measurements were performed with a heating rate of 20 °C/min, in the temperature range of 25–1000 °C. Samples were weighed without further treatment in 100 μL alumina crucibles. The results were baseline-corrected, and then evaluated using the thermal analyzer’s processing software (AKTS Calisto Processing, ver. 2.15). In parallel with the TG measurements, the analysis of the evolved gas was carried out on a Pfeiffer Vacuum OmniStar™ (Pfeiffer Vacuum, Asslar, Germany) gas analysis system. The gas splitter and transfer line to the mass spectrometer was preheated to 200 °C. The scanned *m*/*z* interval was 11–80 amu, with a scan speed of 50 ms amu^−1^. The mass spectrometer was operated in electron impact mode.

The conductivity of the powdered electrocatalysts was assessed using a home-made device in a two-electrode cell arrangement (Figure S4 in the Supplementary Materials). Details of the method are shown elsewhere [18].

### 2.4. Electrochemical Characterization

The electrochemical measurements were performed using standard three-electrode electrochemical cell and a Biologic SP 150 potentiostat (BioLogic, Seyssinet-Pariset, France). The electrolyte was 0.5 M H_2_SO_4_ solution prepared from Milli-Q water and concentrated H_2_SO_4_. The working electrode was made of glassy carbon (GC; d = 0.3 cm) and had a surface area of 0.0707 cm^2^. A hydrogen electrode was used as reference electrode, while a platinum wire was used as counter electrode. All potentials are given on reversible hydrogen electrode (RHE) scale. The detailed description of the working electrode preparation, the catalyst ink composition and electrocatalytic measurements can be found in Refs. [58,61]. The Pt loading of working electrodes was 10 µg cm^−2^.

Cyclic voltammetry, CO_ads_-stripping voltammetry and short-term (500-cycle) and long-term (10,000-cycle) stability tests were performed to calculate the electrochemically active Pt surface area (ECSA), to investigate the CO oxidation activity and to assess the stability of the 20 wt.% Pt/Ti_0.8_Mo_0.2_O_2_-C electrocatalysts. Linear sweep voltammetry tests were performed on rotating disk electrodes (RDEs) in order to investigate the ORR activity. Further details are described in the Supplementary Materials (Section S2.2).

The electrochemical performance of the newly developed electrocatalysts, involving activity in ORR and long-term stability, was compared with that of commercial 20 wt.% Pt/C (Quintech).

## 3. Results

### 3.1. Preliminary Results Obtained by Use of GNPs from Different Sources

Our previous results have shown that a relationship exists between the specific surface area of the parent carbonaceous material and that of the composite formed: the larger the surface area of the parent carbon, the larger the surface area of the composite [17]. Moreover, if the carbon did not provide a surface large enough to accommodate TiO_2_, e.g., the TiO_2_/C ratio was too high, the desired Mo incorporation under the HTT was only marginal, and separate Mo-oxide phases were formed [62]. Thus, it could be concluded that the carbon material behaves as a template during preparation of composites byour sol–gel based method. We have also found in our previous work that the SSA_BET_ of the composite Ti_(1−x)_Mo_x_O_2_-C (C: Black Pearls 2000, Vulcan XC-72) decreased significantly compared to the parent carbon material [17]. This observation can be explained by pore blocking and/or partial filling of pores and spaces between particles during the formation of the TiO_2_-based mixed oxide. In fact, SSA_BET_ is one of the metrics which is obtained on the base of physisorption measurements and it is strongly connected to the pore structure. In Section 2.1, the surface area provided by the manufacturer was listed for the investigated commercially available GNPs. In addition, in a series of experiments, we also investigated the N_2_ physisorption behavior of these GNP materials, in order to choose the appropriate parent carbon for further experiments. The main parameters of the various parent GNPs and the composites obtained from these measurements are summarized in Table 2 and Table S1, Figures S5–S7 in the Supplementary Materials.

Regarding the XRD patterns of parent GNPs (Figure S7A in the Supplementary Materials), it can be seen that GNP-S1, which had lower SSA_BET_ than the other two GNP materials, showed much higher intensity at around 2θ = 26° (graphite (002) reflection). This is in fact reasonable, since the higher crystallinity of GNP-S1, as proven by XRD, would lead to lower SSA_BET_. This high intensity peak attributed to the carbon is observed in the GNP-S1-based composite material (100GNP-S1) before (Figure S7B line a) and after (Figure S7C line a) the HTT, while such a peak was not detected for 100GNP-S2 and 100GNP-NG composites. XRD patterns of all the composites after the drying step at 85 °C proved the presence of rutile-TiO_2_ nuclei (Figure S7B). The intensities of the peaks characteristic of the mixed oxide part increased after HTT, which reveals that the crystallinity of the samples was improved by HTT (see Figure S7C). Although XRD results proved that the desired rutile phase was formed and no peak attributed to MoO_2_ was obtained, demonstrating the successful incorporation for all supports (Figure S7C), the synthesis process resulted in significant SSA_BET_ drop, and the values of total pore volume were moderate (Table 2) in every case. The isotherms of all three 100GNP samples were similar to each other; they showed type IV isotherms according to the IUPAC classification [63] with a slight H3 type hysteresis loop, characteristic for slit-like mesopores with wide pore size distribution (see Figure S6 in the Supplementary Materials). Compared to the parent GNP supports, the specific surface area significantly decreased due to the penetration of precipitated metal oxides into the mesopores and to some pore blocking effects. It can be seen from the data of Table 2 that the SSA_BET_ of all three composites was close to the recommended value required for electrocatalyst support materials (100 m^2^/g [64]).

Consistent with the idea of the role of carbon as a template, 100GNP-S2 has the highest SSA_BET_; consequently, GNP-S2 was chosen for further experiments.

### 3.2. Physicochemical Characterization of Composite Supports and Supported Pt Electrocatalysts

#### 3.2.1. Characterization of Composite Type of Catalyst Supports

Results of the N_2_ physisorption experiments of the composite type of supports prepared from GNP-S2 and GO are summarized in Table 3; the isotherms are presented in Figure S8 in the Section S3.2.1 of the Supplementary Materials.

The shapes of the isotherms are very similar to each other but slightly differ from that of pure GNP-originated ones (cf. Figures S6 and S8 in the Supplementary Materials). According to IUPAC classification [63], the support materials have a type IV isotherm, with an H3/H4 type of hysteresis loop that denotes aggregated flat particles and reflects the structure of the parent carbonaceous material—that is, the graphene-oxide sheets of the delaminated GO. The specific surface area and total pore volume values are in the same range; no correlation was found between the GO or GNPs content and SSA_BET_ or pore volume.

**Figure 1:**
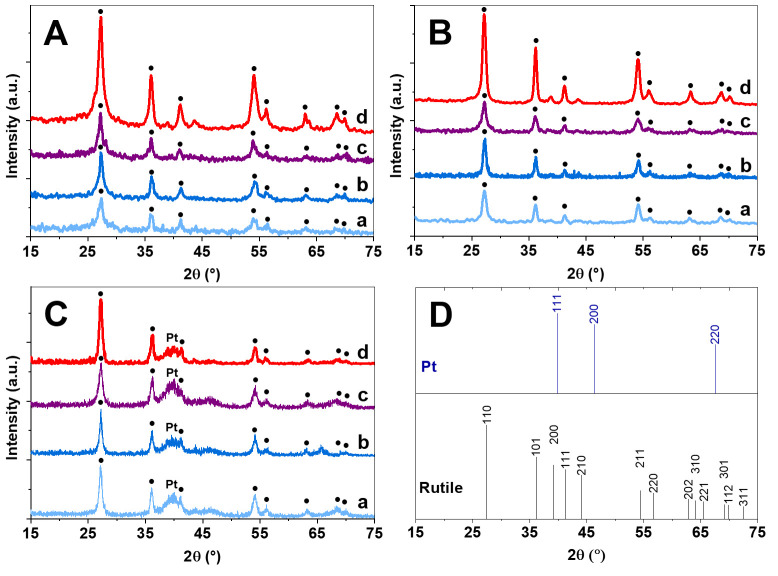
XRD patterns of the composite samples from GNP-GO mixtures with various GNPs/GO ratios in the different stages of their preparation. (**A**) before HTT, (**B**) after HTT, (**C**) after Pt loading. a: 25GNP-S2, b: 50GNP-S2, c: 75GNP-S2, d: 100GNP-S2, ●—rutile. (**D**) Reflection positions for rutile TiO_2_ (JCPDS card no. 21-1276) and Pt (JCPDS card no. 87-0647).

The changes in the oxide part of the composite during the preparation can be followed using the XRD measurements of the samples from the different stages of the modified sol–gel procedure (Figure 1).

Rutile nuclei appeared in all samples during the aging step of TiO_2_ with the carbonaceous material (Figure 1A), which led to the predominant formation of rutile, i.e., crystalline titania-based phases detected by XRD contain exclusively rutile after the HTT, as we found in our previous works [17,18,58]. No separate crystalline Mo-oxide could be observed. The calculated lattice parameters of the samples after the HTT were the same for all samples (a~4.63 Å, c~2.94 Å), which indicated approximately 18% Mo incorporation. The broad applicability of our synthesis method is demonstrated by the fact that the structural properties of the supports met the expectations. The average (primary) oxide particle size calculated from the XRD results was between 9 and 13 nm, and also showed no correlation with GO or GNPs content (Table 3). It should be noted that, according to our previous experience, the oxide phase exists in large, star-like aggregates and smaller units, although their ratio may vary significantly from sample to sample, depending on parameters such as SSA_BET_ or functionalization of the carbon [17].

**Figure 2:**
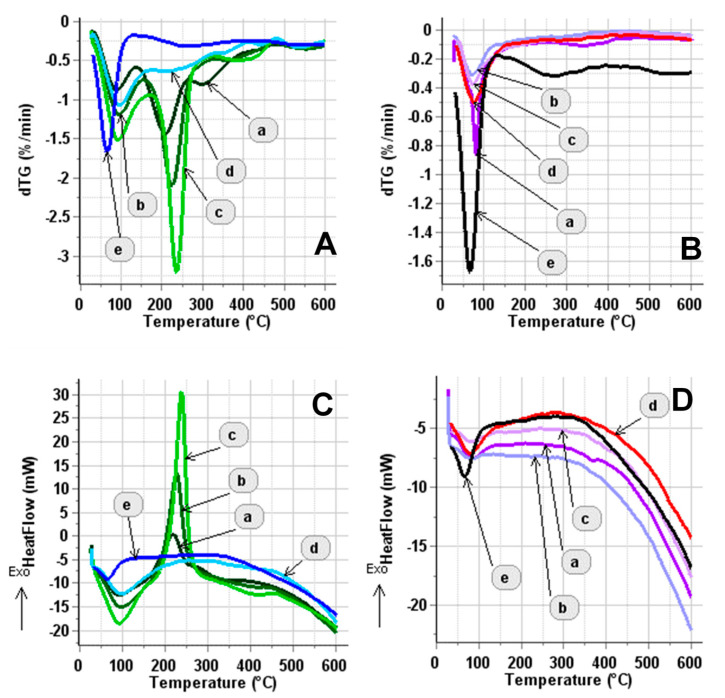
Mass loss rate (DTG) and heat flow curves of Ti_(1−x)_Mo_x_O_2_-C composites before and after HTT. Appropriate curves of parent GNP-S2 are given for comparison. (**A**) DTG curves of samples before HTT; (**B**) DTG curves of samples after HTT; (**C**) heat flow curves of samples before HTT; (**D**) Heat flow curves of samples after HTT; a: 75GNP-S2; b: 50GNP-S2; c: 25GNP-S2; d: 100GNP-S2; e: parent GNP-S2 as-received.

The changes in the carbonaceous part of the composite during the preparation were followed by the simultaneous thermogravimetric (TG) and mass spectrometric (MS) evolved gas analyses performed on the samples from the different stages of the modified sol–gel procedure. The motivation for the above measurements is that GO is thermally unstable [65]; their functional groups, which are known to be heat-sensitive, are gradually lost with increasing temperature [66]. However, high-temperature treatment is required for the dopant element to be incorporated into the rutile-TiO_2_ phase. The results of the thermal analysis are summarized in Figure 2 and in Tables S2–S6 (Section S3.2.2 in the Supplementary Materials).

It can be seen that thermal changes in the composites before HTT can be divided into two main intervals, such as low-temperature changes (range up to~150 °C) and medium-temperature changes (range ~150–300 °C), both well-distinguishable in the case of samples obtained after the drying step at 80 °C (Figure 2A,C). The resulting features are proportional to the GO content in both the DTG (Figure 2A) and heat flow (Figure 2C) measurements. Based on the TG-MS trace (Section S3.2.2 in the Supplementary Materials), it could be concluded that the low-temperature region of up to ~150 °C is related to the removal of adsorbed water. The parallel formation of H_2_O, CO_2_, CO, and formaldehyde (H_2_CO) between ~150–300 °C is due to partial removal of the oxygen-containing functional groups from the carbonaceous part of the composite, in line with the literature finding related to the decomposition of pure GO [66]. Increasing GO content in the composite led to increasing mass loss rate and heat flow (exothermic) in the region of ~150–300 °C. Ref. [66] summarized that oxygen-containing groups of GO were generally decomposed in three temperature regions of 170–250, 500–600 and 750–1000 °C. In our case, i.e., the composite with incorporated GO, the decomposition in the middle region mentioned in the literature is hardly detectable.

It should also be noted that in the case of the samples after HTT (see Figure 2B,D and Section S3.2.2 in the Supplementary Materials), the signal intensities were much lower and almost no changes between ~150–300 °C were observed. Obviously, as a result of the high-temperature heat treatment, only moisture desorption was present in these cases. In the case of the parent GNP, the signal intensity is the weakest in all regions, proving that there are only a very limited number of functional groups on GNPs (line e in Figure 2).

All these observations indicate that not only the mixed oxide part of the composite, but also its carbonaceous content, undergo significant changes during HTT. In good agreement with the TG-MS investigations, XPS measurements also confirmed removal of a significant amount of the functional groups of GO, resulting in similar, graphite-like C 1s spectra for all investigated composites.

**Figure 3:**
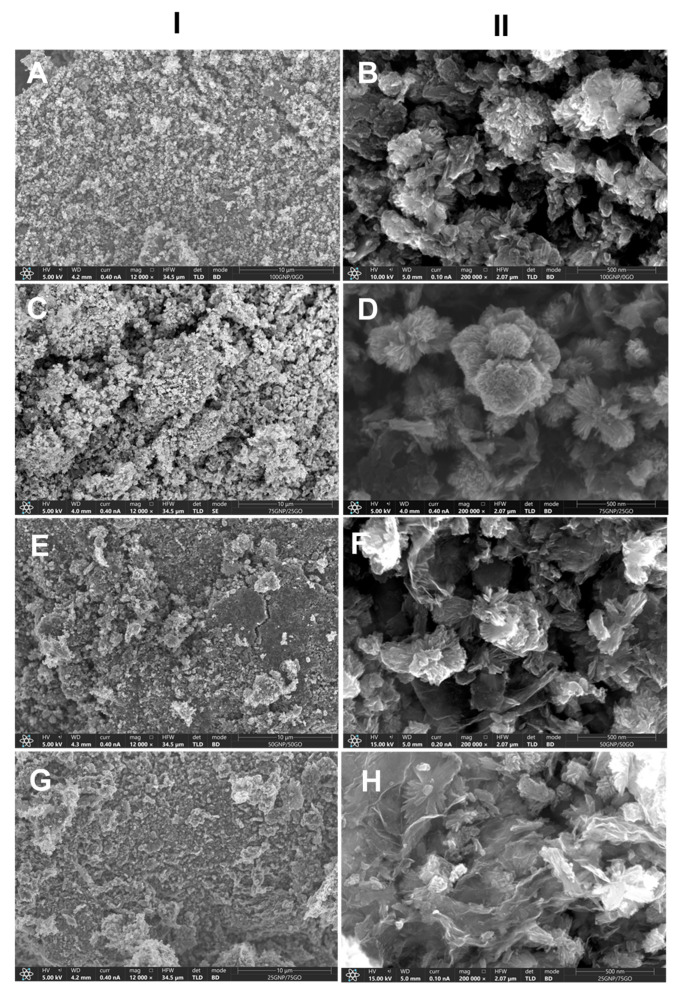
SEM images of Ti_(1−x)_Mo_x_O_2_-C composites prepared from GNP-GO mixtures with different GNPs/GO ratios. (**A**,**B**) 100GNP-S2, (**C**,**D**) 75GNP-S2, (**E**,**F**) 50GNP-S2, (**G**,**H**) 25GNP-S2. Column I: 12,000× magnification; Column II: 200,000× magnification.

SEM images provided information about the morphology of the surface of the composites with different GNPs/GO ratios (see Figure 3). The texture of the composite gradually changed with increasing GO content. Layer-like structures attributed to the GO-derived material became more dominant from composite to composite, alongside irregular rectangles derived from GNPs.

#### 3.2.2. Characterization of Composite-Supported Platinum Catalyst

According to XRD measurement (Figure 1C), all the composite-supported catalysts contained Pt in highly dispersed form. A broad band at about 40° (Pt(111) reflection) appeared (Figure 1C) in all samples. Average crystallite size values of Pt calculated from these patterns were relatively similar (see Table 4).

This result can be attributed to the EG-assisted NaBH_4_ reduction (see details in Section S1.2 in the Supplementary Materials), which was found to be a very effective Pt loading method in our previous works [17]. The Pt content of the various samples measured by ICP-OES, a bulk technique, was only slightly less than the nominal value (Table 4). The same can be observed in the case of the introduced Mo dopant. In contrast, XPS revealed a very high apparent Pt content for all samples; this can be explained by the localization of the Pt particles on the surface of relatively large support grains. A notable feature is that the apparent oxide content derived from XPS measurements is smaller in all samples than the nominal value. In fact, this phenomenon was generally observed for mixed oxide–carbon composite materials [17] and was explained by the existence of large oxide crystals: if a part of the oxide material is present in the form of particles thicker than the information depth of XPS (around 10 nm), its amount is underestimated, as the oxide material in the interior of these particles is not accessible for the method. At the same time, the surface Mo content was slightly higher (i.e., smaller Ti/Mo value) than that observed by ICP-OES, but the sample-to-sample differences were small (Table 4).

The XPS results concerning the chemical states of the components of the electrocatalyst samples are detailed in Section S3.2.3 in the Supplementary Materials (Table S7, Figures S9 and S10). In fact, many characteristics such as the completely oxidized nature of Ti and the coexistence of the dominant Mo^6+^ states with the less abundant Mo^5+^ and Mo^4+^ states in the mixed oxide, the metallic nature of Pt or the predominantly graphitic state of the carbonaceous component were very similar to those documented in other mixed oxide–carbon composite-supported systems [17,18]. C 1s spectra (Figure S10) demonstrated the transformation of the GO component into a predominantly graphitic material during the catalyst synthesis procedure: its functional groups containing carbon singly bound to oxygen were largely eliminated or (in a small fraction) further oxidized into a carboxyl/lactone-like state [67]. Nevertheless, both the C 1s and O 1s spectra revealed that the amount of these types of oxygen-containing functional groups slightly increased with higher nominal GO content. As no correlation was observed by TG-MS between the functional group content of the composites and their GO content after HTT, this kind of functionalization can be regarded as a surface phenomenon.

**Figure 4:**
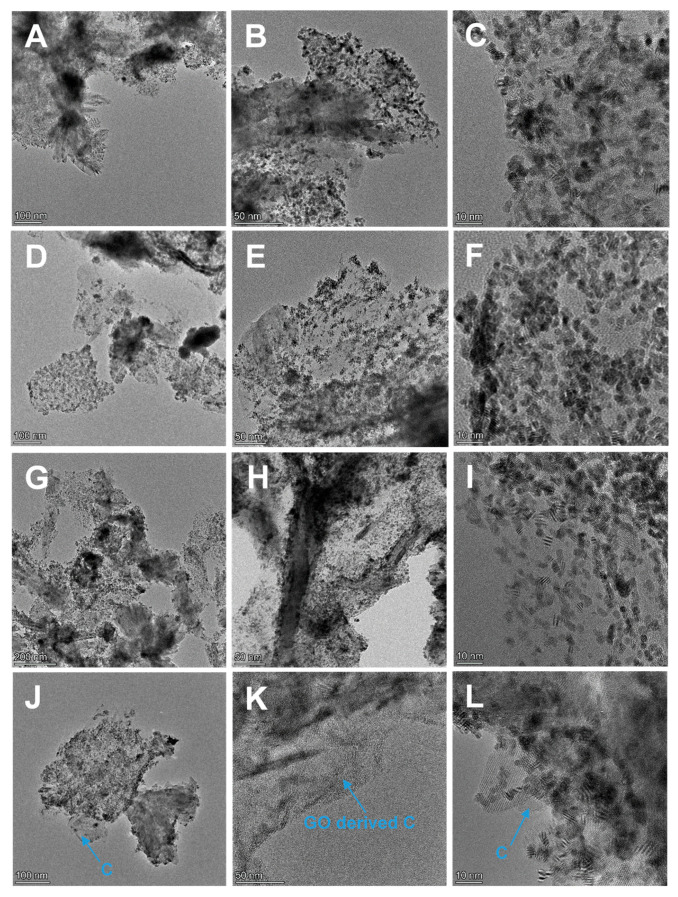
TEM images of Pt/Ti_(1−x)_Mo_x_O_2_-C catalysts prepared from GNP-GO mixtures with different GNPs/GO ratios. (**A**–**C**) Pt/100GNP-S2, (**D**–**F**) Pt/75GNP-S2, (**G**–**I**) Pt/50GNP-S2, (**J**–**L**) Pt/25GNP-S2.

TEM micrographs of the electrocatalyst samples are shown in Figure 4 and Figure S11. At low magnification (Figure S11A,C,E) the objects providing dark contrast are large mixed oxide crystals, which frequently formed flower-like aggregates and often covered the major part of the catalyst grains. In case of the Pt/100GNP-S2 electrocatalyst, individual catalyst grains were scattered through the field of view (Figure S11A). If the support contained GO, sheets, sometimes with wrinkled paper-like appearance, were frequently attached to the grains carrying the large oxide crystals; these sheets are identified as the derivatives of GO formed during the synthesis of the composite support (Figure S11C,E). In the case of the Pt/50GNP-S2 sample (Figure S11C), the sheets formed interconnections between the grains with large oxide crystals. In the case of the Pt/25GNP-S2 catalyst (Figure 4J,K,L and Figure S11E), the sheet-like objects represented the major part of the backbone of the support. Micrographs taken at higher magnification (Figure 4 and Figure S11B,D,F) confirm that Pt existed in a highly dispersed form of particles, which in places formed some small aggregates, while the mixed oxide appeared both in the form of small and large crystals in all samples. Smaller oxide crystals with low contrast were typically found in the sheet-like regions (Figure S11D,F). At high resolution, apart from the abundant fringes of the Pt nanoparticles, occasionally fringes separated by some 0.342–0.344 nm were also visible, clearly demonstrating the presence of multilayered carbon particles (Figure S11B,G). The more ordered carbon particles (Figure S11B) can be identified as GNPs or their fragments formed during composite synthesis. Since these features were observable in all samples, it has to be assumed that the support grains always contained a considerable amount of stacked GNPs and/or GNP fragments. The less ordered ones may also be regarded as agglomerated sheets of GO reduced by HTT.

**Figure 5:**
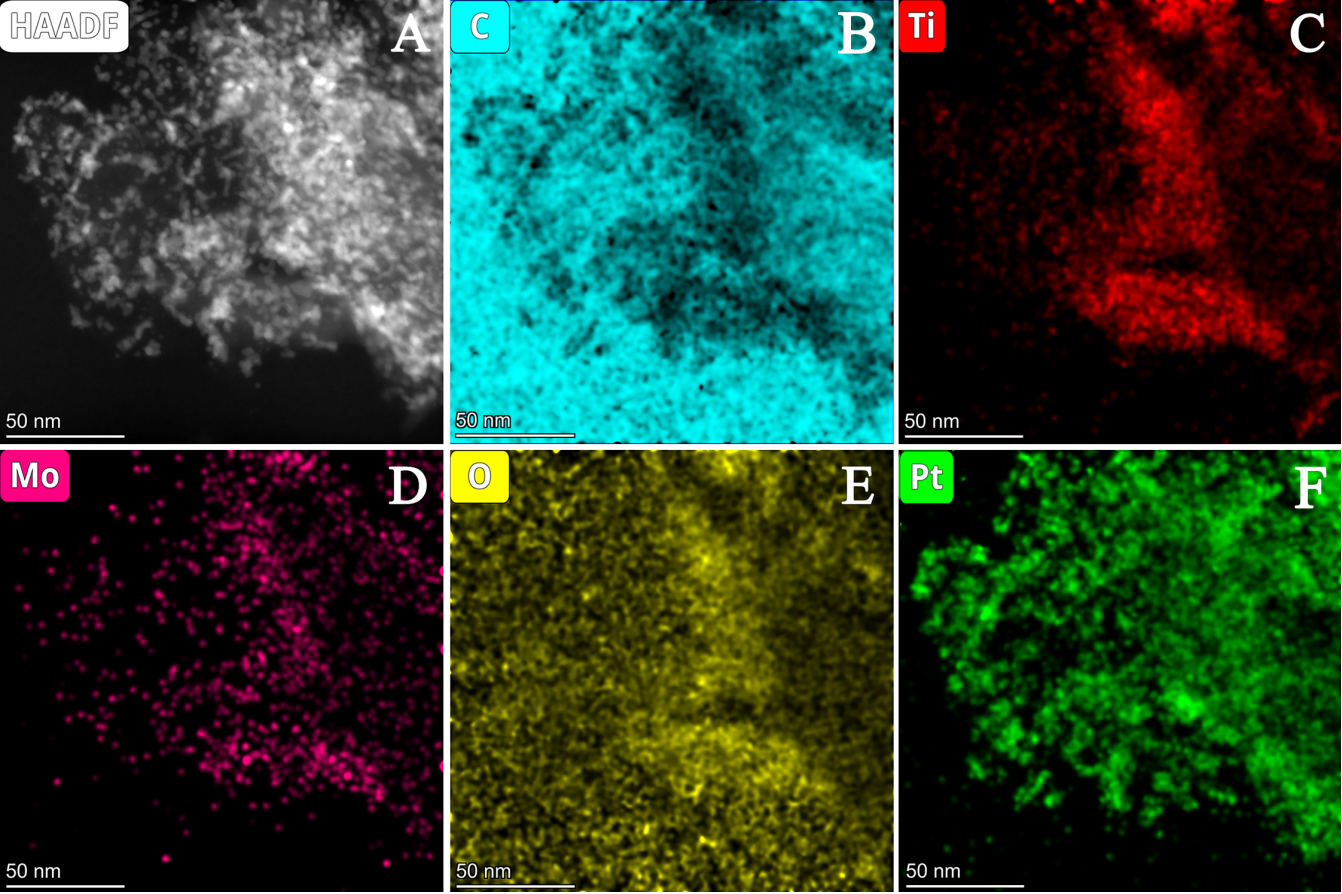
Elemental map of Pt/100GNP-S2 electrocatalyst. (**A**) HAADF, (**B**) C, (**C**) Ti, (**D**) Mo, (**E**) O, (**F**): Pt.

**Figure 6:**
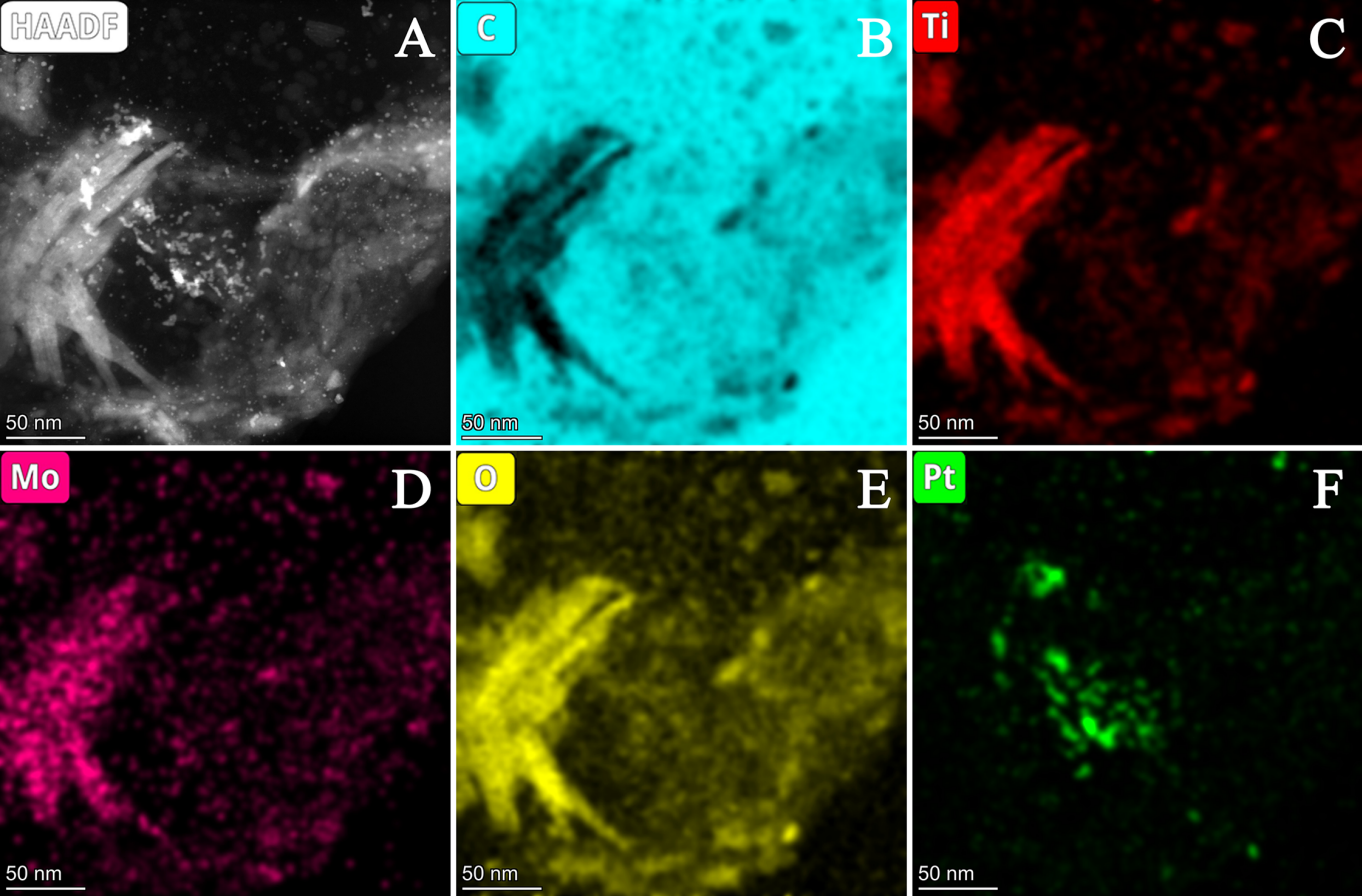
Elemental map of Pt/25GNP-S2 electrocatalyst. (**A**) HAADF, (**B**) C, (**C**) Ti, (**D**) Mo, (**E**) O, (**F**) Pt.

Element maps of Pt/100GNP-S2 (Figure 5) were almost completely congruent for Ti, O and Mo which can be proof that Mo incorporated and/or tightly bound to the mixed oxide surface, as we found previously [18]. It is conceivable that the formation of the bent shapes seen in the O, Ti and Mo element maps was induced by the shape of the GNP particles (Figure 5C–E). The elemental map of Pt was less strongly correlated with those of Mo and Ti, indicating slightly more spreading of the active metal on the surface (Figure 5F). A similar phenomenon can be observed in the element map of the Pt/25GNP-S2 sample (Figure 6C–E). The Ti, O and Mo maps of the Pt/25GNP-S2 sample also clearly show the existence of curved shapes. The possibility cannot be excluded that these objects originated from the mixed oxide formed at the edges of the GNP. However, the Pt map did not follow the aforementioned pattern. The curved shapes are also found in the element maps of samples Pt/75GNP-S2 and Pt/50GNP-S2 (see Figures S12 and S13 in the Supplementary Materials).

**Figure 7:**
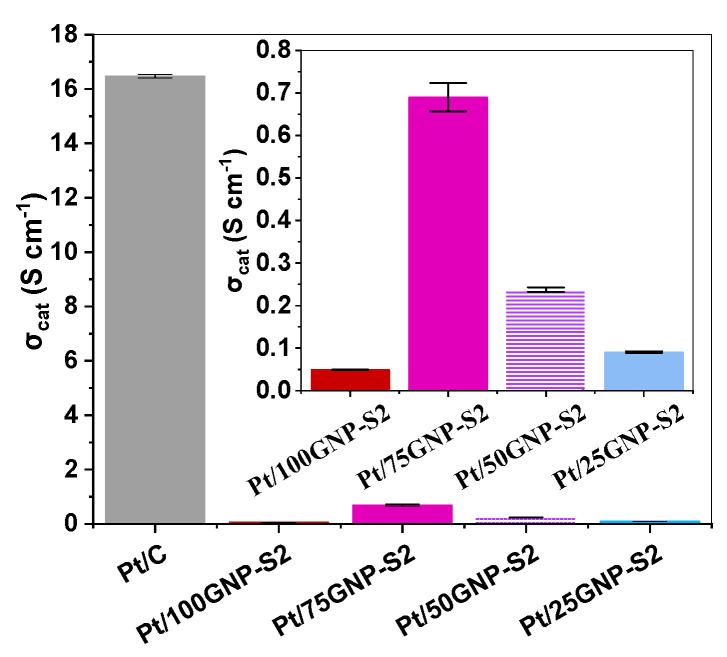
Electrical conductivities of the catalysts. Pt/C: commercial reference catalyst (Quintech).

The electrical conductivity of all composite-supported catalysts was much lower than that of the Pt/C catalysts (see Figure 7). This is not a surprise, because only a quarter of the weight of the support was carbon, and the rest was built from a TiO_2_-based mixed oxide, which can be considered a semiconductor. The conductivity of samples containing the GNPs-GO mixture-derived carbon decreased with decreasing GNPs content, which can be explained by the excellent conductivity of the starting GNPs, while GO is a worse conductor even in its graphitized form.

Surprisingly, the pure GNPs-originated catalyst had the lowest electrical conductivity in these series. A possible explanation is that the carbonaceous aggregates of the support, carrying and/or intermixed with the oxide, are separated by the (frequently large) surface oxide crystals (Figure S11A), as the formation of TiO_2_ starts at the functional groups located at the edges of the little GNP units and spreads from there.

**Scheme 1:**
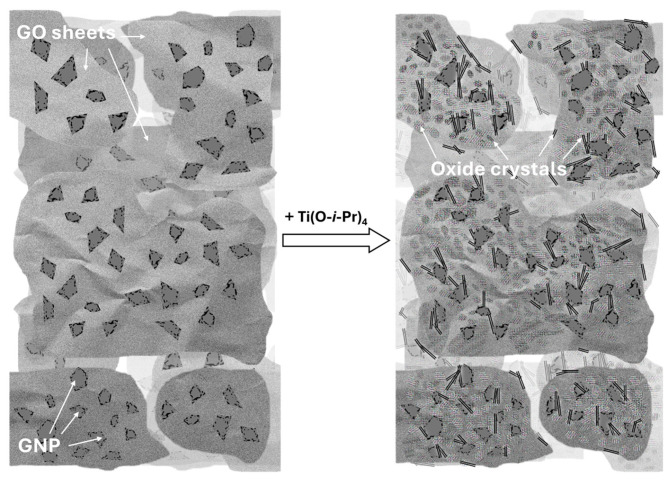
Schematic view of the sticking of the GNP particles to the surface of the delaminated GO sheet (**left**) and formation of the oxide particles around the GNP particles and on the GO sheet at the beginning (**right**). According to the results described in this and previous works [17], some of the oxide particles develop into large rod-like structures (represented by black sticks), which may form flower-like aggregates, while other oxide particles remain small and give minimal contrast in TEM, although their presence is revealed by strong Pt-oxide coupling in electrochemical experiments (light-gray oxide elements).

However, if there is a close interaction between the larger GO sheets and GNP units, as seen in the TEM image published in the literature (Figure 4 in Ref. [48]) and as depicted in Scheme 1, or GO bridges are formed between the GNP nanoparticles or their assemblies in the starting carbonaceous material, a percolating carbon network is formed during titania formation in the mixed oxide–carbon composite, which contributes to the increased conductivity. We believe such a situation is shown in Figure S11C. It is important to mention that the intense ultrasonic treatment, which is necessary to apply during aqueous synthesis due to the strongly apolar nature of GNPs, is able to rupture the GO sheets and reduce the extension of the carbon networks.

The increasing presence of the GO derivatives with increasing nominal GO content was already suggested by the SEM results of the composite support (Figure 3). If one assumes that the increasing GO content results in better interconnections, while GO is a worse conductor than GNPs, even the optimum observed in the conductivity–GO content relationship (Figure 7) can be understood.

In summary, based on the physicochemical characterization of the catalysts, it can be concluded that the composite-supported catalyst samples prepared with and without GO showed similar oxide structures and similarly high dispersion and homogeneous distribution of Pt. However, their texture and electrical conductivity clearly reflected the presence of GO in the system.

### 3.3. Electrochemical Behavior of Pt/Ti_(1−x)_Mo_x_O_2_-C Composite Type of Catalysts Derived from GNPs-GO Mixtures with Different GNPs/GO Ratios

The effect of the GNPs/GO ratio in the mixture of two carbonaceous components used for the preparation of the Ti_0.8_Mo_0.2_O_2_-C composite-supported 20 wt.% Pt catalysts on the electrochemical performance was investigated.

In addition, the results obtained on the reference Pt/C and catalysts containing only GNPs (Pt/100GNP-S2) or GO (Pt/100GO) as the starting carbonaceous materials are also presented.

**Figure 8:**
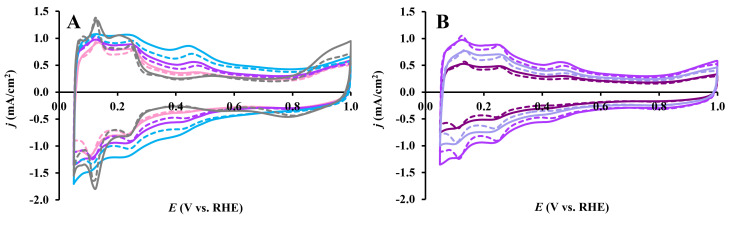
Cyclic voltammograms of the electrocatalysts recorded in 0.5 M H_2_SO_4_ before (solid curves) and after 500 cycles (dashed curves) of the stability test: (**A**) Pt/100GNP-S2 (█), Pt/50GNP-S2 (█), Pt/100GO (█) and reference Pt/C (█); (**B**) Pt/75GNP-S2 (█), Pt/50GNP-S2 (█) and Pt/25GNP-S2 (█). Sweep rate: 100 mV/s.

The cyclic voltammograms of the electrocatalysts recorded in 0.5 M H_2_SO_4_ before and after 500 polarization cycles of the stability test are demonstrated in Figure 8; for comparison, Figure 8A also shows the CVs of the reference Pt/C catalyst. As shown in Figure 8, the voltammograms of all Mo-containing catalysts exhibited the usual redox peak pair at 380–530 mV in addition to the typical under-potentially deposited hydrogen adsorption/desorption between 50 and 350 mV characteristic of Pt catalysts (see CV of the reference Pt/C).

As shown in Figure 8, the voltammograms of all Mo-containing catalysts exhibited the usual redox peak pair at 380–530 mV in addition to the typical under-potentially deposited hydrogen adsorption/desorption between 50 and 350 mV characteristic of Pt catalysts (see CV of the reference Pt/C). The presence of these Mo redox peaks confirms the statement in the literature [68,69,70] that an active interface exists between the Pt nanoparticles and the Mo-containing support, as already demonstrated in our earlier studies [61,71].

According to the extensive literature on this subject [68,70], the redox peak pair observed in Mo-containing Pt catalytic systems is generally considered to be the intercalation/de-intercalation of H atoms into the MoO_x_ lattice leading to the formation/decomposition of hydrogen molybdenum bronze species and, as a consequence, should be subtracted from the calculated H_UPD_ charges.

The electrochemically active Pt surface area of the catalysts determined for all catalysts in the first cycle (see ECSA_1_ values presented in Table 5) was calculated from the oxidation charge of the monolayer hydrogen [72], taking into account the capacitive currents, originated from the double-layer charging of the CVs [73,74] and considering the above-mentioned overlapping features of different origin in the voltammograms of the Mo-containing catalysts, which may lead to some uncertainty in the results obtained.

As can be seen from Figure 8, the presence of Mo redox peaks of different sizes prevents the use of a consistent potential range for calculating the ECSA values using the H_UPD_ method. By slightly modifying the potential ranges used for H_UPD_ integration for the Mo-containing catalysts presented in this work, it was possible to perform the calculation in such a way that the Mo oxidation peak was not included. This may result in slightly lower ECSA values compared to catalysts where such peaks are absent, as in the case of the reference Pt/C catalyst, or where these redox peaks are not as pronounced. However, it should be emphasized that in the case of Mo-containing catalysts, these overlapping peaks can be separated much more easily compared to, for example, the Pt/WO_x_ type of electrocatalysts, in which the peak observed in the negative-going scan at 200 mV vs. RHE, attributed to the formation of tungsten bronzes H_y_WO_3_ or substoichiometric tungsten oxides WO_3-x_ [76], excludes the possibility of determining the ECSA using the H_UPD_ method.

It should be noted that, as shown in Table 5, in this series of experiments there was a fairly large difference in the ECSA_1_ values obtained for the different catalysts prepared using GNPs-GO mixture with various GNPs/GO ratios. According to the ECSA_1_ values shown in Table 5, Pt/75GNP-S2 has the lowest electrochemically active Pt surface area, which can be related the lowest Pt content (16.5 wt.%), determined in this series of experiments by ICP-OES (see Table 4). In addition to the slightly lower Pt loading, the markedly lower ECSA_1_ observed for Pt/75GNP-S2 can be attributed to the microstructural characteristics of the support at this specific GNPs/GO ratio. Although this sample exhibits a relatively high BET surface area, its lower total pore volume and potentially less favorable pore connectivity likely limit electrolyte access to a portion of the Pt surface. In contrast, the Pt/50GNP-S2 catalyst, which contains a more balanced distribution of GO anchoring sites and GNP conductive domains with a more open composite structure, facilitates uniform Pt dispersion and improved electrochemical accessibility. This indicates that the relationship between GNPs/GO ratio and ECSA is not linear, and that optimal Pt utilization is achieved when sufficient GO is present to provide enough functional groups for anchoring nanoparticles [77], while GNP content remains high enough to maintain electronic conductivity and structural openness for appropriate mass transport. Since Pt/50GNP-S2 has both higher intrinsic surface (slightly higher loading) and a more favorable support microstructure, the absolute number of electrochemically accessible Pt sites is much larger than in Pt/75GNP-S2, resulting in higher ECSA_1_.

As shown in Figure 8, only relatively small changes in the voltammogram shape after the 500 polarization cycles of the stability tests can be seen on the catalysts containing pure GNPs (Figure 8A) and GNPs-GO mixtures (Figure 8B). As demonstrated in Figure 8A, the most pronounced change was observed on the Pt/100GO catalyst and the reference Pt/C.

Table 5 summarizes the values of the ECSA loss observed after the 500 and 10,000 polarization cycles of the stability tests (ΔECSA_500_ and ΔECSA_10,000_, respectively) between 50 and 1000 mV potential limits. For convenience, the ΔECSA_500_ values presented in Table 5 were calculated from data collected during stability measurements over 10,000 polarization cycles. As already mentioned above, the most pronounced changes were observed on the Pt/100GO catalyst (see Figure 8A), which was reflected in the highest ΔECSA_500_ value obtained on the Mo-containing catalysts presented in Table 5. But an even higher ΔECSA_500_ value was obtained on the commercial Pt/C catalyst (ΔECSA_500_ = 12.7%).

**Figure 9:**
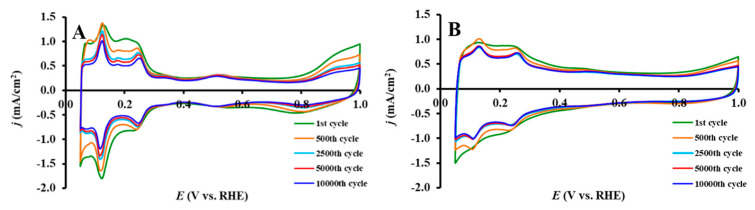
Cyclic voltammograms of the reference Pt/C (**A**) and Pt/50GNP-S2 (**B**) electrocatalysts obtained during a 10,000-cycle stability test. Recorded in 0.5 M H_2_SO_4_ solution with 100 mV s^−1^ sweep rate; T = 25 °C.

However, in this regard, a more informative comparison of the stability of the catalysts studied can be obtained based on the results of the long-term stability tests. The changes in the shape of the cyclic voltammograms obtained during the 10,000 polarization cycles are demonstrated in Figure 9 and Figure S17 in the Supplementary Materials. Figure 9 compares the results obtained on the reference Pt/C and the Pt/50GNP-S2 sample, while the cyclic voltammograms of other composite-supported catalysts are presented in Figure S17.

As expected, during the long-term stability test, the main changes in the shape of the voltammograms are associated with the hydrogen adsorption/desorption peaks between 50 and 350 mV and the Pt oxidation/reduction peaks above 800 mV. As shown in Figure 9, the difference in the behavior of the commercial Pt/C and the most stable Pt/50GNP-S2 electrocatalyst during polarization for 10,000 cycles becomes obvious after 2500 cycles: while on the Pt/50GNP-S2 catalyst the obtained cyclic voltammograms of the 2500, 5000 and 10,000 cycles are practically indistinguishable (see Figure 9B), the reference Pt/C demonstrates much more significant changes (Figure 9A).

**Figure 10:**
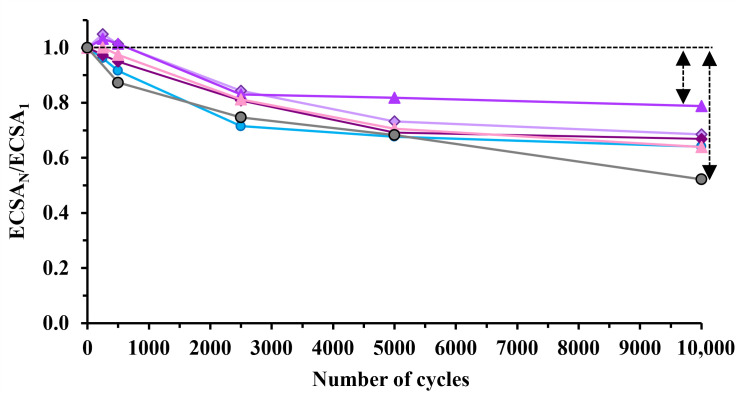
Change in the ECSA during 10,000 polarization cycles for the reference Pt/C (█) and composite-supported electrocatalysts: Pt/100GNP-S2 (█), Pt/75GNP-S2 (█), Pt/50GNP-S2 (█), Pt/25GNP-S2 (█) and Pt/100GO (█). ECSA_N_/ECSA_1_ values are compared as a function of the cycles number.

The changes in the ECSA over 10,000 CV cycles for the reference Pt/C and Mo-containing Pt electrocatalysts are presented in Figure 10.

Similar behavior in the long-term stability tests was recently demonstrated for Sn-containing composite-supported electrocatalysts [78]. For the Pt/Ti_0.9_Sn_0.1_O_2_–C catalytic systems, it was noticed that the initial period (up to 2500 polarization cycles), characterized by a pronounced decrease in the normalized ECSA values, was followed by a period of a very moderate decrease in the ECSA_N_/ECSA_1_ values. This behavior is confirmed in Figure 10 for the Pt/50GNP-S2 electrocatalyst, where the observed changes on this catalyst after 2500 cycles are very small. As shown in Figure 10, a similar behavior to the Pt/50GNP-S2 catalyst is also observed for the Pt/100GO sample, showing a significant slowdown in the decrease rate in the normalized ECSA value after 2500 polarization cycles. However, as can be seen from Figure 10, for the other three GNP-containing catalysts, a similar pronounced slowdown in the decline of the normalized values of the ECSA is observed only after 5000 cycles of cyclic polarization. Meanwhile, for Pt/C, a constant decrease in the ECSA value is characteristic throughout the 10,000-cycle stability test. Thus, taking into account the tendency of a sharper drop in the electrochemical surface area of Pt in the reference Pt/C catalyst, it can be assumed that when conducting longer experiments, the difference in the stability of the reference and composite-supported Pt electrocatalysts will be even more pronounced.

Figure 10 also presents the results obtained from the 500-cycle stability test, which demonstrate some small increase in ECSA during the short-term stability tests for two catalysts (Pt/50GNP-S2 and Pt/25GNP-S2), reflected by the negative values for ΔECSA_500_ presented in Table 5. This behavior may be due to the cleaning of the catalyst surface from remaining contaminants or oxides that have a blocking effect [18,75].

Based on our synthesis route (for details see Section 2.2), the most probable blocking species that are electrochemically removed during the first cycles are (i) organic-origin residues (adsorbed ethylene glycol decomposition products, incomplete reduction by-products or remaining solvent fragments), (ii) adsorbed anions (residual Cl^−^ from Pt precursor and from the HCl treatment) that can block adsorption sites and (iii) native Pt oxides/hydroxides (PtO_x_) formed on the metal surface during handling and drying. In addition, oxygen-containing functional groups on GO (–OH, –COOH, epoxide) and impurities in the parent GO suspension can both partially block Pt sites or alter Pt-support interaction and change during electrochemical conditioning. It is important to note that our protocol includes four centrifugal washing cycles to remove chloride; nevertheless, trace Cl^−^ cannot be fully excluded. In addition, in our previous study it has been demonstrated [75] that a similar effect is observed during reductive pretreatment of Mo-containing Pt electrocatalysts, which leads to the migration of Mo onto the surface of Pt particles and the blocking of this surface. During the first cycles of the polarization, removal of these species and releasing the Pt particle surface may lead to a temporary increase in the ECSA values.

In our previous studies, we found that carbon materials behave as a solid template during the synthesis of composite materials using our sol–gel method. The key to high stability is obtaining the homogeneous distribution of mixed oxide layers or particles over a carbon backbone [17,30]. The best results on electrocatalysts with 20 wt.% Pt were obtained using composite supports with increased carbon content (50–75 wt.%) and/or upon using functionalized carbon materials [17,30]. We propose that carbon functionalization alters to some extent the nucleation and growth of the mixed oxide particles over the carbon surface, resulting in a more uniform distribution of the mixed oxide.

As can be seen from Figure 10 and Table 5, the most pronounced ECSA decrease was observed on the reference Pt/C (ΔECSA_10,000_ = 47.8%). The best stability after 10,000 polarization cycles was demonstrated by the Pt/50GNP-S2 catalyst with a GNPs/GO = 50/50 ratio, which is reflected in the lowest values of ΔECSA_10,000_, presented in Table 5 (ΔECSA_10,000_ = 21.2%). The other two catalysts, prepared using the GNPs-GO mixture with different GNPs/GO ratios, have slightly higher ΔECSA values (31 and 33%; cf. ΔECSA_10,000_ values for the Pt/25GNP-S2 and Pt/75GNP-S2 catalysts in Table 5). However, it should be noted that catalysts prepared using only GNPs (Pt/100GNP-S2) or GO (Pt/100GO) as starting carbonaceous materials also exhibit good stability (~36%), characteristic of Mo-containing systems with high mixed oxide content (Ti_0.8_Mo_0.2_O_2_/C ratios of 75/25 [17]).

Our best results from the 10,000-cycle stability tests obtained on 20 wt.% Pt/Ti_0.8_Mo_0.2_O_2_-C catalysts prepared using different carbonaceous material and various post-treatments are summarized in Table 6.

According to the results presented in Table 6, the combination of various unmodified, functionalized and non-traditional carbon materials with different treatments carried out during or after sol–gel synthesis resulted in innovative supported Pt electrocatalysts with excellent long-term stability. Thus, as shown in Table 6, the results for the Pt/50GNP-S2 catalyst, containing GNPs-GO mixture with the ratio of 50/50, are among the best ones we have obtained on Mo-containing composite-supported Pt catalysts.

High CO electrooxidation activity is critical to maintaining the performance and longevity of fuel cells, since the presence of even small traces of CO in hydrogen gas leads to the poisoning of catalysts and deteriorates their activity [79,80]. The traditional Pt/C catalysts are able to oxidize CO only at about 800 mV [81,82]. However, under practical fuel cell operating conditions, such a high potential is never reached at the anode. In this regard, the key factor determining CO tolerance is the ability of electrocatalysts to oxidize CO at low potentials (below 600 mV). From this point of view, a state-of-the-art PtRu/C catalyst may be a good choice. However, the instability of Ru increases significantly at anode potentials ~450 mV vs. RHE, leading to the dissolution of ruthenium and degradation of the catalyst [83].

It is well known in the literature [80] and our studies [58] that, due to the ability of molybdenum to adsorb hydroxyl groups via a bifunctional mechanism, catalysts containing Mo have increased resistance to CO poisoning and can oxidize CO even at very low electrode potentials. As has been repeatedly noted in our previous studies [17,61], the most important indicator of the CO-tolerant behavior of these catalytic systems is the increase in current in the “pre-peak” region (observed between 50 and 500 mV), which precedes the main peak of CO electrooxidation at higher potential. In this regard, our previous results have demonstrated the increased CO tolerance and better electrocatalytic stability of the Pt/Ti_0.8_Mo_0.2_O_2_-C electrocatalyst compared to the PtRu/C [57]. We demonstrated that CO electrooxidation began on the Mo-containing catalysts at 50 mV, while on the PtRu/C the E_CO,onset_ was observed at 255 mV.

It should be noted that the “pre-peak” region is complex and includes contributions from the oxidation of both weakly bound CO on Pt in close proximity to Mo, which provides the OH_ads_ required for the reaction at low potential, and the oxidation of Mo surface species [61]. On the other hand, it can be argued that the presence of a “pre-peak” region on the CO_ads_-stripping voltammogram demonstrates the close location of Pt and easily reducible Mo species [30,84,85].

Considering the above-mentioned facts, it is important to emphasize that there is no relationship between the magnitude of the main peak of CO electrooxidation, which in the case of the reference Pt/C catalyst was observed at around 805 mV (see Table 5), and the activity in this reaction.

**Figure 11:**
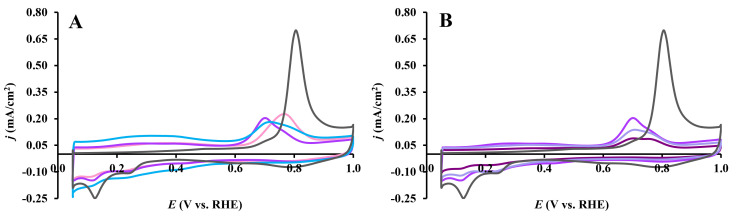
CO_ads_-stripping voltammograms of the electrocatalysts recorded in 0.5 M H_2_SO_4_: (**A**) Pt/100GNP-S2 (█), Pt/50GNP-S2 (█) and Pt/100GO (█); (**B**) Pt/75GNP-S2 (█), Pt/50GNP-S2 (█) and Pt/25GNP-S2 (█). Results obtained on the reference Pt/C (█) were included for comparison. Sweep rate: 10 mV/s.

The activity of the prepared Mo-containing Pt catalysts and the reference Pt/C in the CO electrooxidation was studied using CO_ads_-stripping voltammetry measurements, and the results obtained are presented in Figure 11.

The activity in this reaction is expressed by both the initial potential (E_CO,onset_) and the position of the main peak (E_CO,max_) of CO oxidation [17,61]. As can be seen from Figure 11, for all Mo-containing composite-supported catalysts presented in this study, the E_CO,onset_ of the CO oxidation did not exceed 50–100 mV. The low E_CO,onset_ and the presence of a “pre-peak” are evidence that in a PEM fuel cell, at the operating anode voltage, the electrode containing this catalyst is capable of oxidizing CO.

Confirmation of this was obtained in previously conducted fuel cell experiments carried out using a CO contaminant containing H_2_ [86] and reformate gas [87].

As shown in Figure 11, a characteristic “pre-peak” is observed on all CO_ads_-stripping voltammograms of Mo-containing catalysts; the most pronounced “pre-peak” area was observed on the Pt/100GO catalyst (see Figure 11A). In our recent study [17], this behavior was explained by the presence of a fairly large number of very small Pt nanoparticles (less than 1 nm) in this sample.

In addition, it should be noted that for the studied Mo-containing catalysts, the ECSA values determined from CO stripping are systematically lower than those derived from H_UPD_ (by approx. 27–37% for the synthesized GO-containing electrocatalysts and ~9% for Pt/100GNP sample). Considering this fact, and the presence of a CO pre-oxidation feature, which appears at significantly lower potentials than the main CO oxidation peak, the use of the main CO oxidation peak alone for ECSA determination can underestimate the true ECSA values for these materials. This is why we rely on H_UPD_-derived ECSA values when comparing intrinsic activity.

The values of the position of the main CO stripping peak maximum (E_CO,max_), observed on the CO_ads_-stripping curves on these electrocatalysts, are presented in Table 5. As follows from Table 5 and Figure 11, all GO-containing catalysts showed a CO_ads_-electrooxidation peak at about 705 mV overlapping with a shoulder at 745 mV. On the pure GNP-containing composite-supported catalyst (Pt/100GNP), the broad CO_ads_-stripping peak was located at ca. 765 mV, while in the case of the reference Pt/C catalyst, the corresponding peak was at about 805 mV (see Table 5 and Figure 11). According to these results, it can be concluded that the presence of GO in composite materials leads to an increase in the tolerance of catalysts to CO.

In conclusion, enhanced CO tolerance of the Pt/Ti_0.8_Mo_0.2_O_2_-C catalytic systems was evidenced by the appearance of a CO-oxidation-related “pre-peak” and by a considerable shift in the maximum of the main CO oxidation peak towards less positive potential compared to the reference commercial Pt/C.

**Figure 12:**
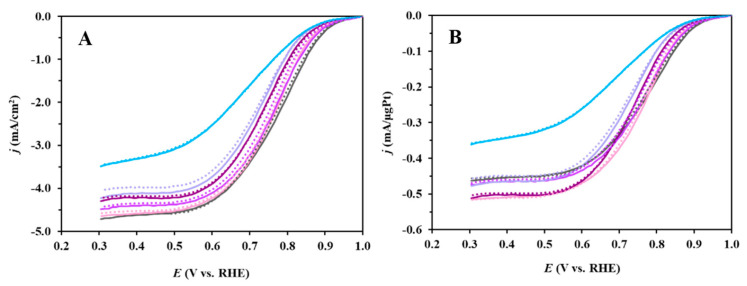
Electrochemical characterization in the ORR obtained before (solid lines) and after 500 cycles (dotted lines) of the stability test presented as current density in mA/cm^2^ (**A**) and mass activity in mA/μg_Pt_ (**B**) of the reference Pt/C (█) and Pt/Ti_0.8_Mo_0.2_O_2_-C electrocatalysts: Pt/100GNP-S2 (█), Pt/75GNP-S2 (█), Pt/50GNP-S2 (█), Pt/25GNP-S2 (█) and Pt/100GO (█). *j* vs. *E* curves were recorded in O_2_-saturated 0.5 M H_2_SO_4_ on an RDE at 1600 rpm. Sweep rate: 10 mV/s. The catalysts’ Pt loading is included in Table 4.

Given their high CO tolerance, these catalysts are generally recommended for use on the anode side of fuel cells, but taking into account the existence of the close contact between Pt and Mo, it is also worth investigating their properties in the ORR. As already mentioned in Section S2.2 of the Supplementary Materials, the activity of these catalysts in the ORR was studied by the RDE technique in 0.5 M H_2_SO_4_ solution saturated with O_2_ at six rotation speeds (225, 400, 625, 900, 1225 and 1600 rpm). Increasing the electrode rotation speed (see Figure S14 in the Supplementary Materials), as expected, resulted in an increase in the current density (*j*) in the potential dynamic polarization curves, indicating faster diffusion of oxygen to the catalyst surface. Figure 12 compares the ORR catalytic activity of the commercial Pt/C and Mo-containing Ti_0.8_Mo_0.2_O_2_–C composite-supported Pt electrocatalysts at 1600 rpm. Using the same rotation speed allows a direct and fair comparison between the reference Pt/C and the newly synthesized catalyst under identical hydrodynamic conditions.

First of all, it should be emphasized that the interpretation of the results of RDE measurements obtained on powder electrocatalysts with a high SSA should be carried out with caution [88,89]. However, in the case of studying catalysts with similar composition and structure, using the same method of electrode preparation, the RDE results can facilitate their qualitative comparison.

Very close values of the onset potentials for the ORR (E_ORR,onset_~955 ± 10 mV) were observed for all catalysts studied, indicating good activity in this reaction. The electrocatalytic efficiency of the catalysts in the ORR was presented as values normalized with respect to the geometric area of the electrode (Figure 12A), and as mass activity (MA) in units of mA/μg_Pt_ (Figure 12B).

The ORR current density in the mixed kinetic–diffusion controlled region presented in Figure 12 and Table 5 (cf. MA values at 0.9 V vs. RHE) decreased in the following order: Pt/C > Pt/50GNP-S2 > Pt/100GNP-S2 > Pt/75GNP-S2 > Pt/25GNP-S2 > Pt100GO. In addition, the MA results obtained at 0.85 V and 0.9 V on the Pt/Ti_0.8_Mo_0.2_O_2_-C electrocatalysts with Ti_0.8_Mo_0.2_O_2_/C = 75/25 mass ratio prepared in this work and in ref. [18] are also visualized in Figure S15. As shown in Figure S15, between the catalysts synthesized in this work, the highest activity in the ORR was obtained on the Pt/50GNP-S2 and Pt/100GNP-S2 samples; the best activity among other catalysts with a mass ratio of Ti_0.8_Mo_0.2_O_2_/C = 75/25 presented in ref. [18] was obtained on the GNP-NG-containing catalyst.

Current density and MA obtained in the ORR at different potentials on the Pt/Ti_0.8_Mo_0.2_O_2_-C catalytic systems with different Ti_0.8_Mo_0.2_O_2_/C mass ratios are compared in Table S8 in the Supplementary Materials. In Table S8, the results obtained on Pt/Ti_0.8_Mo_0.2_O_2_-C electrocatalysts with a Ti_0.8_Mo_0.2_O_2_/C ratio of 75/25 were compared with the results presented in our recent study on electrocatalysts with higher carbonaceous materials content [90]. As follows from Table S8, among other catalysts with a Ti_0.8_Mo_0.2_O_2_/C mass ratio of 25/75, the catalyst containing 75 wt.% of Vulcan XC-72 was the most promising.

As shown in Table S8, the mass activity of the Pt/Ti_0.8_Mo_0.2_O_2_-C (C: functionalized Black Pearls 2000 (FBP)) catalyst with Ti_0.8_Mo_0.2_O_2_/C = 50/50 mass ratio reported in [75] was comparable with the activity of both the GNP-containing catalysts with lower carbon content (Ti_0.8_Mo_0.2_O_2_/C = 75/25). The reductive pretreatment resulted in a significant increase in the MA values. In this series of experiments, the highest MA was observed in the catalyst reduced at 250 °C (Pt/FBP-250H); only this catalyst reached the mass activity of the commercial Pt/C.

In conclusion, it should be noted that the catalytic systems presented in this study contain too much oxide in their composition (Ti_0.8_Mo_0.2_O_2_/C = 75/25 mass ratio) to be used as a cathode. As is known, the load of the electrocatalyst on the cathode side, due to the slow kinetics of the oxygen reduction reaction, is approximately three times higher than on the anode side. Therefore, high oxide content typically causes increased resistance and mass transfer limitations, resulting in low activity. In this regard, GNP-GO mixture-containing catalytic systems may be of practical interest for use as a cathode only in the case of using catalysts with a lower content of mixed oxide (e.g., Ti_0.8_Mo_0.2_O_2_/C ratio of 25/75). Furthermore, based on the previous results, reduction of the most promising catalysts to create strong metal-support interactions may be the next strategy to enhance the activity of the oxygen reduction reaction.

As shown in Figure 12, as expected, fairly close values of the diffusion-limited current are observed for the reference Pt/C and all GNP-containing catalysts. The only exception is the result obtained for the Pt/100GO catalyst, apparently indicating the difference in the support morphology and/or structural characteristics of the support. It should be noted that in oxide-containing Pt catalysts, the lower *j*_lim_ can also be attributed to reversible oxide formation/reduction on Pt [91] and to slower oxygen diffusion through the oxide layer covering the Pt nanoparticles [92,93].

As can be seen in Figure 12A, very similar diffusion-limited currents were obtained on the Pt/100GNP-S2, Pt/50GNP-S2 and commercial Pt/C electrocatalysts, while the limiting current of the other two GNP-containing catalysts was slightly lower. Since, according to the Koutecký–Levich equation, in the presence of an ideal non-porous catalyst layer on the working electrode, the diffusion-limited current density (*j*_lim_) depends only on the rotation rate, thus it should theoretically be the same. In this regard, we should emphasize that the difference in the *j*_lim_ observed in Figure 12 on the GNP-containing catalysts was less than 10%. Note that, according to Mayrhofer et al. [74], for carbon-containing supported catalysts, this difference in the diffusion-limited current values observed in the ORR measurements is within the expected relative measurement errors. As shown in Figure 12B, representing ORR activity in units of mass activity only slightly affects the order of the *j*_lim_ decrease: in this case, the highest limiting current was observed on the Pt/100GNP-S2 and Pt/75GNP-S2 catalysts.

In additional experiments, the ORR measurements were carried out before and after the 500-cycle stability test. Figure 12 compares the ORR catalytic activity obtained before and after the short-term stability test of the commercial Pt/C and Mo-containing composite-supported Pt electrocatalysts at 1600 rpm. As shown in Figure 12, after 500 cycles, only minor changes in the polarization curves were observed for all the catalysts studied.

Figure S16 in the Supplementary Materials compares the mass activity values at 0.9 V for both the commercial reference Pt/C and the catalysts synthesized in this work calculated for the ORR measurements conducted before and after stability tests. As can be seen from Figure S16, the MA obtained on the Pt/75GNP-S2 and Pt/25GNP-S2 catalytic systems during the ORR carried out after the 500-cycle stability test was higher compared to the result obtained in the first ORR experiment. This behavior may be due to changes in the surface structure of the catalysts after the first ORR experiment.

As illustrated in Figure S16, the catalysts synthesized in this work exhibit lower initial mass activities than the commercial Pt/C, which is expected given that Pt/C serves as a well-optimized benchmark catalyst with highly uniform dispersion and controlled particle size distribution.

It should be noted that these results represent only short-term behavior; the 500-cycle polarization test is insufficient to draw definitive conclusions about long-term stability. Indeed, as we demonstrated above (see Figure 10) and in our previous study conducted on Pt/Ti_0.9_Sn_0.1_O_2_-C catalysts, after 2500 cycles the rate of activity decay for the composite-supported catalysts becomes significantly lower than that of Pt/C, indicating better durability under prolonged operation [78].

In conclusion, it should be noted that among the Mo-containing electrocatalysts, the best and similar activity in the ORR was obtained on the Pt/50GNP-S2 and Pt/100GNP-S2 electrocatalysts. Moreover, in this series of experiments, the Pt/50GNP-S2 catalyst demonstrated the highest long-term stability (ΔECSA_10,000_ = 21.2%), comparable to the best results we had previously obtained on Pt/Ti_0.8_Mo_0.2_O_2_-C catalytic systems. In addition, this catalyst with a GNPs/GO ratio of 50/50 had an increased CO tolerance, similar to that of the Pt/100GO.

Thus, based on the obtained results, it can be stated that the use of the GNPs-GO mixture with a GNPs/GO = 50/50 ratio as carbon material in the preparation of a composite support allows us to combine the beneficial properties of GNPs and GO, which leads to high activity and stability of the resulting catalyst.

## 4. Conclusions

In conclusion, 20 wt.% Pt/(75 wt.% Ti_0.8_Mo_0.2_O_2_—25 wt.% C)-type composite-supported electrocatalysts were successfully prepared from GNP and GNPs-GO mixtures. According to XRD measurements, mixed oxide existed in the desired rutile phase in all samples.

Regarding the results of the physicochemical characterization of the sample series with varying GO content, it can be concluded that both the large-scale texture of the composite-supported catalysts and the functional group content of the carbonaceous component of their support reflected the increasing presence of GO beside GNPs. It facilitates formation of a favorable, more dispersed oxide layer, similarly to the use of functionalized carbonaceous materials. The electrical conductivity values were significantly higher when the GNPs-GO mixtures were used as the starting carbon material of the composite, compared to the use of pure GNPs, indicating a percolating carbonaceous network in the composite.

In the case of all the composite-supported electrocatalysts, increased CO oxidation activity was achieved due to the incorporated Mo, and the stability of the composite-supported Pt catalysts was significantly higher than that of commercial Pt/C. Furthermore, the increased stability of GNPs-GO mixture-containing catalyst compared to the GO- and GNP-derived mixtures was observed. Based on these results, we believe that introduction of the GO component improved the surface chemical properties of the GNP-based hard template due to its functional groups, which were partially retained after the high-temperature treatment phase of the composite synthesis and can serve as anchoring sites for Pt nanoparticles.

## Figures and Tables

**Table 1:** Denomination and preparation details of Ti_(1−x)_Mo_x_O_2_-C (C = GNP, GNP-GO derived carbonaceous material) composites. Nominal composition: 75 wt.% Ti_0.8_Mo_0.2_O_2_-25 wt.% C.

Composite	Carbon Source	GNPs/GO Ratio ^1^	TiO_2_ Sol			Suspension of Carbonaceous Material ^4^			Mo Prec. ^2^, g	Ref.
			Ti Prec. ^2^, mL	HNO_3_ ^3^, mL	H_2_O, mL	GNP, g	GO Sol, g (% ^5^)	H_2_O, mL		
100GNP-NG	GNP ^6^	100/0	2.100	2.35	21	0.250	0	10	0.299	[18]
100GNP-S1	GNP ^7^	100/0	2.100	2.35	21	0.250	0	10	0.299	this work
100GNP-S2	GNP ^8^	100/0	2.100	2.35	21	0.250	0	10	0.299	this work
75GNP-S2	GNP ^8^-GO	75/25	1.079	1.52	29	0.128	3.01 (1.4)	9	0.117 ^9^	this work
50GNP-S2	GNP ^8^-GO	50/50	1.079	1.52	29	0.085	6.02 (1.4)	6	0.117 ^9^	this work
25GNP-S2	GNP ^8^-GO	25/75	1.079	1.52	29	0.043	9.03 (1.4)	3	0.117 ^9^	this work

^1^ The amount of GO was calculated according to the C_8_O_4_H_2_ general formula of GO. ^2^ Ti and Mo precursor compounds: Ti(O-i-Pr)_4_ and (NH_4_)_6_Mo_7_O_24_ × 4H_2_O. ^3^ cc. HNO_3_ (65%, Molar Chemicals, a.r.). ^4^ pH adjusted to 9 with concentrated NaOH solution. ^5^ concentration of GO in the sol. ^6^ Nanografi, specific surface area = 700 m^2^/g. ^7^ Sigma-Aldrich No900394, average surface area = 300 m^2^/g. ^8^ Sigma-Aldrich No900407, average surface area = 750 m^2^/g. ^9^ NaNO_3_ removal (washing step) before adding Mo precursor.

**Table 2:** Characterization of the composite catalyst supports prepared by GNPs from various sources (nominal value of Ti_(1−x)_Mo_x_O_2_/C = 75 wt.%/25 wt.%, nominal value of Ti/Mo = 4/1 at/at).

Composite	Parent GNP			Composites			
	Physisorption ^1^		XRD	Physisorption ^1^		XRD	
	SSA_BET_, m^2^/g	Total Pore Volume, cm^3^/g	D ^2^, nm	SSA_BET_, m^2^/g	Total Pore Volume, cm^3^/g	Lattice Parameters ^3^, Å	Mo Subst., %
100GNP-NG	700	1.08	9	98 ^4^	0.14 ^4^	a~4.63, c~2.94 ^4^	18 ^4^
100GNP-S1	347	0.62	20	92	0.13	a~4.63, c~2.94	18
100GNP-S2	1068	1.64	9	136	0.20	a~4.63, c~2.94	18

^1^ SSA_BET_ and total pore volume determined by low temperature N_2_ physisorption measurements. ^2^ Average crystallite size calculated from Scherrer equation. ^3^ Lattice parameters of the rutile phase after HTT; Pure rutile TiO_2_: a = 4.593 Å, c *=* 2.959 Å. ^4^ From Ref. [18].

**Table 3:** Characterization of the composite type of catalysts supports prepared from GNP-GO mixtures (nominal value of Ti/Mo = 4/1 at/at, nominal value of Ti_(1−x)_Mo_x_O_2_/C = 75 wt.%/25 wt.%).

Composite	Physisorption ^1^		XRD
	SSA_BET_, m^2^g^−1^	Total Pore Volume, cm^3^g^−1^	D ^2^, nm
75GNP-S2	128	0.31	11.2
50GNP-S2	105	0.33	12.5
25GNP-S2	125	0.45	9.1

^1^ SSA_BET_ and total pore volume determined by low temperature N_2_ physisorption measurements. ^2^ Average crystallite size of mixed oxide calculated using the Scherrer equation.

**Table 4:** Characterization of composite-supported Pt catalysts (nominal composition: 20 wt.% Pt/(75 wt.% Ti_0.8_Mo_0.2_O_2_-25 wt.% C); nominal value of Ti/Mo: 4/1).

Catalyst	XRD	ICP-OES		XPS		
	Pt Average Crystallite Size ^1^, nm	Pt, wt.%	Ti/Mo, mol/mol	Pt, wt.%	(Ti + Mo + O)/C ^2^ wt.%/wt.%	Ti/Mo, at%/at%
Pt/100GNP-S2	3.7	17.7	4.8/1	44.0	66.4/33.6	3.8/1
Pt/75GNP-S2	3.0	16.5	5.1/1	51.0	58.1/41.9	3.4/1
Pt/50GNP-S2	2.6	18.6	5.2/1	52.5	60.7/39.3	3.7/1
Pt/25GNP-S2	2.5	17.4	4.8/1	44.7	58.0/42.0	3.7/1

^1^ Average crystallite size of Pt calculated from Scherrer equation; ^2^ Calculated without the Pt content.

**Table 5:** Electrochemical performance of the 20 wt.% Pt/Ti_0.8_Mo_0.2_O_2_-C catalysts prepared using GNP-GO mixtures with different GNPs/GO ratios.

Sample	E_CO,max_^1^, mV	ECSA_1_, m^2^/g_Pt_^2^	ΔECSA_500_, % ^3,4^	ΔECSA_10,000_, % ^3^	MA @ 0.9 V, ^5^ mA/mg_Pt_
Pt/100GO	705 (sh: 745)	79.7 ± 1.8	8.4	36.0	13.3
Pt/25GNP-S2	705 (sh: 745)	60.8 ± 1.7	−1.3	31.4	14.6
Pt/50GNP-S2	705 (sh: 745)	72.4 ± 3.5	−1.4	21.2	26.6
Pt/75GNP-S2	705 (sh: 745)	42.6 ± 3.6	5.0	33.2	19.0
Pt/100GNP-S2	765	63.3 ± 2.3	2.5	36.1	23.8
Pt/C ^6^	805	87.2 ± 2.3	12.7	47.8	33.2

^1^ The position of the main CO stripping peak measured on fresh catalysts; ^2^ The average ECSA_1_ value obtained on fresh catalysts; ^3^ ΔECSA_500_ and ΔECSA_10,000_ were calculated from the charges originated from the hydrogen desorption in the 1st and 500th or 10,000th cycles according to Equation (S2) in the Supplementary Materials (Section S2.2); ^4^ ΔECSA_500_ values were calculated from the 10,000-cycle stability measurements; ^5^ Mass activity (MA) at 0.9 V obtained in the ORR experiments on an RDE at 1600 rp; ^6^ From Ref. [75]; *s*h = should*er*.

**Table 6:** Our best results from the 10,000-cycle stability tests obtained on the Pt/Ti_0.8_Mo_0.2_O_2_-C catalysts.

Type of Carbon	Preparation Details	Oxide/C Ratio	ΔECSA_10,000_, %	Ref.
GNPs/GO = 50/50	sol–gel	75/25	21.2	present work
functionalized BP ^1^	sol–gel/350 °C reduction ^2^	50/50	22.1	[75]
functionalized BP ^1^	sol–gel/250 °C reduction ^2^	50/50	23.8	[75]
GO-derived carbon	sol–gel + ST ^3^	75/25	23.8	[18]
functionalized BP ^1^	sol–gel	25/75	24.1	[17]
unmodified BP ^1^	sol–gel	25/75	27.6	[17]

^1^ BP: Black Pearls 2000; ^2^ Reduction in H_2_ flow for 2 h (to induce the SMSI effect); ^3^ ST: solvothermal treatment.

## Data Availability

The original contributions presented in this study are included in the article/Supplementary Materials. Further inquiries can be directed to the corresponding authors.

## Supplementary Materials

The following supporting information can be downloaded at https://www.mdpi.com/article/10.3390/nano15231753/s1, Figure S1. Flow charts for preparation of Ti_(1−x)_Mo_x_O_2_-C composite electrocatalyst supports by using sol–gel-based multistep synthesis routes from GNPs (A), GO-GNP mixture (B). GNPs: graphene nanoplatelets; GO: graphite oxide; Figure S2. Temperature program for high-temperature heat treatment (HTT); Figure S3. Flow chart of platinum loading; Figure S4. Setup for conductivity measurements; Figure S5. Adsorption isotherms of the parent GNPs, red: GNP-S1; green: GNP-S2, blue: GNP-NG; Figure S6. Adsorption isotherms of Ti_(x−1)_Mo_x_O_2_-C (C: GNP) composites. (A) 100GNP-NG [18], (B) 100GNP-S1, (C) 100GNP-S2 (see denomination of the samples in Table 1 of the main text); Figure S7. XRD patterns of the composite samples from various types of GNP materials in the different stages of their preparation. (A) parent GNP materials, line a: GNP-1S (Sigma-Aldrich No900394, average surface area: 300 m^2^/g); line b: GNP-2S (Sigma-Aldrich No900407, average surface area: 750 m^2^/g); line c: GNP-NG (Nanografi; SSA_BET_: 700 m^2^/g), (B) Composites before high-temperature heat treatment (HTT), line a: 100GNP-1S; line b: 100GNP-2S; line c: 100GNP-NG [18], ●-rutile, *-carbon, (C) Composites after HTT, line a: 100GNP-1S; line b: 100GNP-2S; line c: 100GNP-NG [18], ●-rutile, *-carbon, (D) rutile TiO_2_ (JCPDS card no. 21-1276); Figure S8. Adsorption isotherms of Ti_(x−1)_Mo_x_O_2_-C C: GNP-GO mixture-derived carbon) composites. (A) 75GNP-S2; (B) 50GNP-S2; (C) 50GNP-S2R; (D) 25GNP-S2 (see composition of the samples in Table 1 of the main text); Figure S9. A: Mo 3d and B: Pt 4f core level spectra of (a) Pt/100GNP-S2; (b) Pt/75GNP-S2, (c) Pt/50GNP-S2; (d) Pt/25GNP; Figure S10. A: C 1s and B: O 1s core level spectra of (a) Pt/100GNP-S2; (b) Pt/75GNP-S2, (c) Pt/50GNP-S2; (d) Pt/25GNP-S2; and (e) GO prepared by Hummer’s method; Figure S11. Transmission electron micrographs of Pt/100GNP-S2 (A,B), Pt/50GNP-S2 (C,D,G) and Pt/25GNP-S2 (E,F). Sheet-like objects are emphasized by yellow encircling in (C) and (E). High resolution micrographs (D) and F) were taken from the sheet-like regions. (G) Fragment of the Pt/50GNP-S2 sample at high resolution. The yellow lines, separated by 2.055 nm, are laid over parallel atomic planes with six interlayers between the lines. The average interlayer distance is then 0.342 nm, providing clear indication of multilayered carbon particles; Figure S12. Elemental map of Pt/75GNP-S2 electrocatalyst. (A) HAAD, (B) C, (C) Ti, (D) Mo, (E) O, (F) Pt; Figure S13. Elemental map of Pt/50GNP-S2 electrocatalyst. (A) HAAD, (B) C, (C) Ti, (D) Mo, (E) O, (F) Pt; Figure S14. ORR polarization curves of the reference Pt/C (A) and Pt/Ti_0.8_Mo_0.2_O_2_-C electrocatalysts: Pt/100GNP-S2 (B), Pt/75GNP-S2 (C), Pt/50GNP-S2 (D), Pt/25GNP-S2 (E) and Pt/100GO (F). *j* vs. *E* curves were recorded in O_2_-saturated 0.5 M H_2_SO_4_ on an RDE at 225–1600 rpm. Sweep rate: 10 mV/s; Figure S15. Mass activity obtained in the ORR on an RDE at 1600 rpm at 0.85 V (A) and 0.9 V (B) on the Pt/Ti_0.8_Mo_0.2_O_2_-C electrocatalysts with Ti_0.8_Mo_0.2_O_2_/C = 75/25 mass ratio presented in this work and in Ref. [18]; Figure S16. The mass activities determined at 0.9 V before and after 500 polarization cycles of the synthesized catalysts and the commercial Pt/C evaluated at 1600 rpm rotation speed; Figure S17. Cyclic voltammograms of the Pt/100GO (A), Pt/100GNP-S2 (B), Pt/25GNP-S2 (C) and Pt/75GNP-S2 (D) electrocatalysts obtained during the 10,000-cycle stability test. Recorded in 0.5 M H_2_SO_4_ solution with 100 mV/s sweep rate, T = 25 °C; Table S1. Calculated parameters of parent GNPs based on low temperature N_2_ physisorption; Table S2. Data of the thermal analysis of parent GNP-S2; Table S3. Data of the thermal analysis of sample 100GNP-S2; Table S4. Data of the thermal analysis of sample 75GNP-S2; Table S5. Data of the thermal analysis of sample 50GNP-S2; Table S6. Data of the thermal analysis of sample 25GNP-S2; Table S7. XPS results of 20 wt.% Pt/Ti_(1−x)_Mo_x_O_2_-C, catalysts: Pt/100GNP-S2, Pt/75GNP-S2, Pt/50GNP-S2 and Pt/25GNP-S2; Table S8. Electrochemical performance of the reference 20 wt.% Pt/C (Quintech) and composite-supported Pt catalysts: current density and mass activity obtained in the ORR at different potentials. Refs. [18,57,58,67,72,73,75,90,94] are also cited in the supplementary materials.
