# Supplementary material for: Linking Structure to Electrocatalytic Performance: Graphene Nanoplatelets-Derived Novel Mixed Oxide–Carbon Composites as Supports for Pt Electrocatalysts with Enhanced Stability
Source: Nanomaterials (Basel). 2025 Nov 22;15(23):1753. doi: 10.3390/nano15231753 (PMC12693164; doi:10.3390/nano15231753)
Supplement: Supplementary file 1 [file nanomaterials-15-01753-s001.zip › nanomaterials-3958886-supplementary.pdf]

SUPPLEMENTARY MATERIALS

Linking structure to electrocatalytic performance: Graphene nanoplatelets derived novel mixed oxide-carbon composites as supports for Pt electrocatalysts with enhanced stability

Ilgar Ayyubov[1], Emília Tálas[1], Irina Borbáth[1], Zoltán Pászti[1*], László Trif[1], Ágnes Szegedi[1], Catia Cannilla[2], Giuseppe Bonura[2], Tamás Szabó[3], Erzsébet Dodony[4], András Tompos[1*]
[1]Renewable Energy Research Group, Institute of Materials and Environmental Chemistry, HUN-REN Research Centre for Natural Sciences, Magyar Tudósok körútja 2, H-1117 Budapest, Hungary, ilgar.ayyubov@ttk.hu (A.I.); talas.emilia@ttk.hu (E.T.), borbath.irina@ttk.hu (I.B.); trif.laszlo@ttk.hu (L.T.); szegedi.agnes@ttk.hu (Sz. Á.);
[2]Institute of Advanced Technology for Energy CNR-ITAE, S. Lucia sopra Contesse, 5, 98126 Messina, Italy, catia.cannilla@cnr.it (C.C.), giuseppe.bonura@cnr.it (G.B.);
[3]Department of Physical Chemistry and Materials Science, University of Szeged, Rerrich Béla tér 1, H-6720 Szeged, Hungary, sztamas@chem.u-szeged.hu (T.Sz.);
[4]Institute for Technical Physics and Materials Science, HUN-REN Centre for Energy Research, Konkoly-Thege M. út 29-33, H-1121 Budapest, Hungary, dodony.erzsebet@ek.hun-ren.hu (E.D.);
Correspondence: paszti.zoltan@ttk.hu (Z.P.); tompos.andras@ttk.hu (A.T.); Tel.: +36-1-3826-412 (Z.P.); +36-1-3826-501 (A.T.)

S1. Experimental

S1.1. Preparation of composite type of electrocatalyst supports

Sol-gel procedure for the preparation of $Ti_{(1-x)}Mo_xO_2$-C (C= GNP, GO-GNP derived carbon; x=0-0.2) composite type of electrocatalyst supports was carried out in three separate steps: i) formation and aging of TiO_2 nuclei on the carbonaceous material, ii) addition of Mo-precursor and drying at low temperature, iii) high temperature heat treatment for Mo incorporation (see flow chart in Figure S1). The syntheses were carried out similarly as we have described previously [1]. Intensive sonication was provided by Hielscher UP200S ultrasonic device and in the GO containing mixtures pH was adjusted to 9 with concentrated NaOH solution to reach intense exfoliation. Latter case a washing step with HNO_3 was inserted (Figure S1B) in order to avoid the harmful $NaNO_3$ formation [2].

High temperature heat treatment (HTT) as a final step of synthesis of the sol-gel based support material is performed for the molybdenum incorporation [3]: to replace the Ti^{4+} ions of rutile TiO_2 with Mo^{n+} ions. The heat treatment was performed under argon atmosphere by use of a quartz reactor fitted into a furnace. HTT process consisted of several steps. In order to remove the oxygen from the system totally and to create an inert condition for avoiding burning out of the carbon content of composite materials the reactor was purged with Ar for 3 h at room temperature. Then, the temperature was increased from 25 °C up to 300 °C with the rate of 5 °C/min. After reaching 300 °C the sample was kept at this temperature for 1 h. In the following step, the sample was heated up to 600 °C with rate of 1 °C/min and kept under this condition for 8 h. As the last step, the furnace was turned off and the sample was cooled down in Ar flow. Figure S2 shows the temperature program of the furnace for HTT.

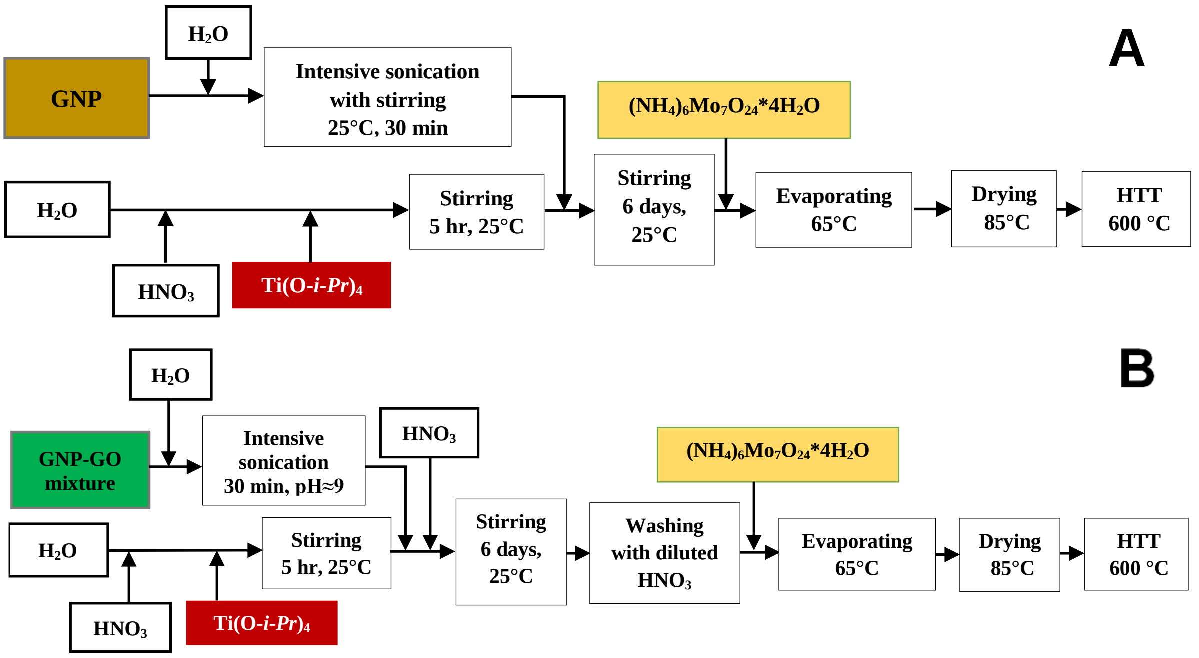

Figure S1. Flow charts for preparation of $Ti_{(1-x)}Mo_xO_2$-C composite electrocatalyst supports by using sol–gel-based multistep synthesis routes from GNP (A), GO-GNP mixture (B). GNP: graphene nanoplatelets; GO: graphite oxide

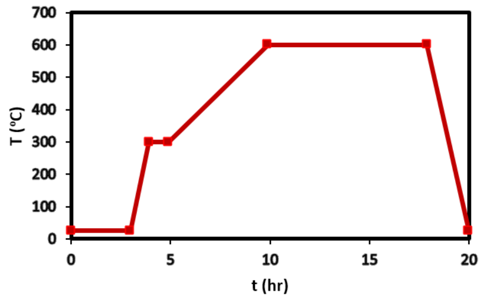

Figure S2. Temperature program for high temperature heat treatment (HTT)

S1.2. Synthesis of Pt electrocatalysts

$Ti_{(1-x)}Mo_xO_2$-C support materials were loaded with 20 wt.% Pt via a modified, sodium borohydride ($NaBH_4$) assisted ethylene-glycol (EG) reduction-precipitation method in order to obtain platinum containing electrocatalyst as we described before [4]. The flow chart of Pt loading process is depicted in Figure S3.

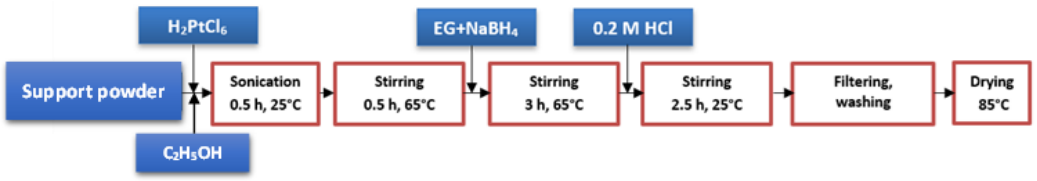

Figure S3. Flow chart of platinum loading

S2. Characterization methods

S2.1. Physicochemical characterization methods

X-ray powder diffraction (XRD) patterns were obtained in a Philips model PW 3710 based PW 1050 Bragg-Brentano parafocusing goniometer using CuKα radiation (λ= 0.15418 nm), graphite monochromator and proportional counter. Silicon powder (NIST SRM 640) was used as an internal standard and the scans were evaluated with profile fitting methods. The cell parameters of the crystalline phases were determined from the fitted values.

Nitrogen physisorption measurements were carried out at temperature of liquid nitrogen using Thermo Scientific Surfer automatic volumetric adsorption analyzer (Thermo Fischer Scientific, Berlin, Germany). The specific surface area was calculated by the BET method in the range of relative pressures from 0.05 to 0.30.

The surface morphology of the samples was investigated using an Ultra-High-Resolution Scanning Electron Microscope (UHR-SEM-FEG, Helios 5 UC DualBeam, Thermo Scientific) equipped with a Field Emission Gun (FEG), which provides high spatial resolution and improved image quality. Elemental analysis and mapping analysis was performed by Energy Dispersive X-ray (EDX) spectroscopy.

Morphology of the electrocatalysts was investigated by TEM in HRTEM and STEM HAADF modes by means of a FEI Titan Themis 200 kV Cs - corrected TEM with 0.09 nm HRTEM and 0.16 nm STEM resolution. Composition of the samples was measured by STEM-EDS.

X-ray photoelectron spectroscopy (XPS) measurements were performed using an EA 125 electron spectrometer manufactured by OMICRON Nanotechnology GmbH (Germany). Ti, Mo and Pt content of the samples was measured by inductively coupled plasma-optical emission spectrometry (ICP-OES) technique by use of a simultaneous SPECTRO GENESIS instrument with axial plasma observation. Samples were measured after microwave assisted dissolution in 1:2:6 mixtures of concentrated hydrofluoric acid: hydrochloric acid: nitric acid.

The simultaneous thermogravimetric (TG) and mass spectrometric (MS) evolved gas analyses were recorded on a Setaram LabsysEvo (Setaram, Lyon, France) thermal analyzer, in high purity (99.9999%) He, with a flow rate of 90 cm^3/min. The measurements were done with a heating rate of 20 °C/min, in the temperature range of 25–1000 °C. Samples were weighed without any sample preparation into 100 µL alumina crucibles. The results were baseline-corrected, and then evaluated with the thermal analyzer's processing software (AKTS Calisto Processing, ver. 2.15). Parallel with the TG measurements, the analysis of the evolved gas was carried out on a Pfeiffer Vacuum OmniStar™ (Pfeiffer Vacuum, Asslar, Germany) gas analysis system. The gas splitter and transfer line to the mass spectrometer was preheated to 200 °C. The scanned m/z interval was 11–80 amu, with a scan speed of 50 ms amu^{-1}. The mass spectrometer was operated in electron impact mode.

Electrical conductivity (σ) was estimated at room temperature, by means of impedance spectroscopy using a home-made equipment utilizing the two-probe principle (Figure S4), as we detailed previously [1].

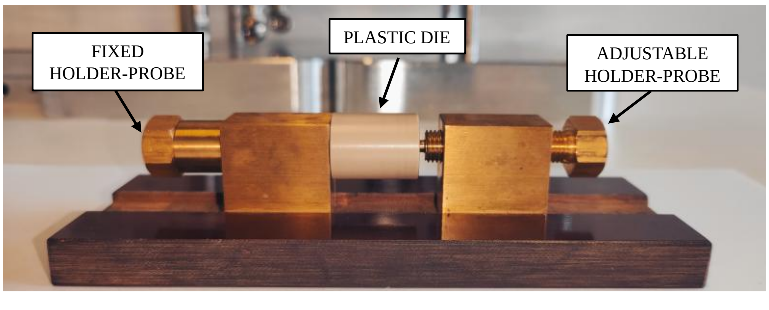

Figure S4. Setup for conductivity measurements

S2.2. Electrochemical characterization

Electrochemically active Pt surface area (ECSA) was determined from the charge needed for oxidation of underpotentially deposited hydrogen on the platinum surface according to the traditional method. ECSA, *i.e.*, the area of the platinum surface on which electrochemical reactions may occur can be determined from CVs in the so-called hydrogen underpotential deposition region, between 0-350 mV, according to Eq. S3 [5,6].

$$\mathbf{ECSA_{Hupd}\ (cm^2) = Q_{ox_{Hupd}}\ (\mu C)\ /\ 210\ (\mu C/cm^2)} \qquad \text{Eq. S1}$$

where $Q_{ox_{Hupd}}$ is the charge accompanies oxidation of underpotentially deposited hydrogen, while 210 µC is the transported charge, while monolayer of H atoms is oxidized over 1 cm^2 Pt surface.

Electrocatalytic performance of the 20 wt.% Pt/$Ti_{0.8}Mo_{0.2}O_2$-C electrocatalysts was studied by cyclic voltammetry and CO_{ads}-stripping voltammetry measurements combined with stability test involving 500 polarization cycles and the second CO_{ads}-stripping voltammetry measurement. In the long-term stability test, the samples were submitted to cyclic polarization at a 100 mV s^{-1} scan rate for 10,000 cycles between 50 and 1000 mV potential limits; these measurements took ca. 54 hours.

The loss in electrochemically active Pt surface area upon N-cycle stability test ($\Delta ECSA_N$; N: 500, 2,500, 5,000 and 10,000) was calculated from the charges originated from the hydrogen desorption in the 1st and N^{th} cycles according to the Eq. S4:

$$\mathbf{\Delta ECSA_N = \{1-(ECSA_N/ECSA_1)\}\times 100\%} \qquad \text{Eq. S2}$$

After every stability test, the electrolyte was changed to fresh one to avoid the re-deposition of the dissolved metals.

Catalytic activity of the catalyst samples was tested in the ORR by rotating disc electrode (RDE) technique. A RDE is a glassy carbon working electrode used in a three-electrode system. The rotating speed of the electrode can be controlled, yielding variable diffusion rate of the reactant. The ORR measurements were done in O_2 saturated 0.5 M H_2SO_4 solution. The diameter of RDE electrode and Pt loading used in these experiments was the same as during CV measurements. Polarization curves were recorded by cathodic scan sweeping the potential between 1000 and 200 mV with 10 mVs^{-1} sweep rate, rotating the electrode at 225, 400, 625, 900, 1225 and 1600 revolutions/min (rpm). In order to characterize the electrochemically active Pt surface area of the catalysts before and after the RDE measurements, 10 CVs between 50 and 1000 mV potential window in Ar saturated electrolyte were also measured.

S3. Results

S3.1. Preliminary results obtained by use of GNPs from different sources

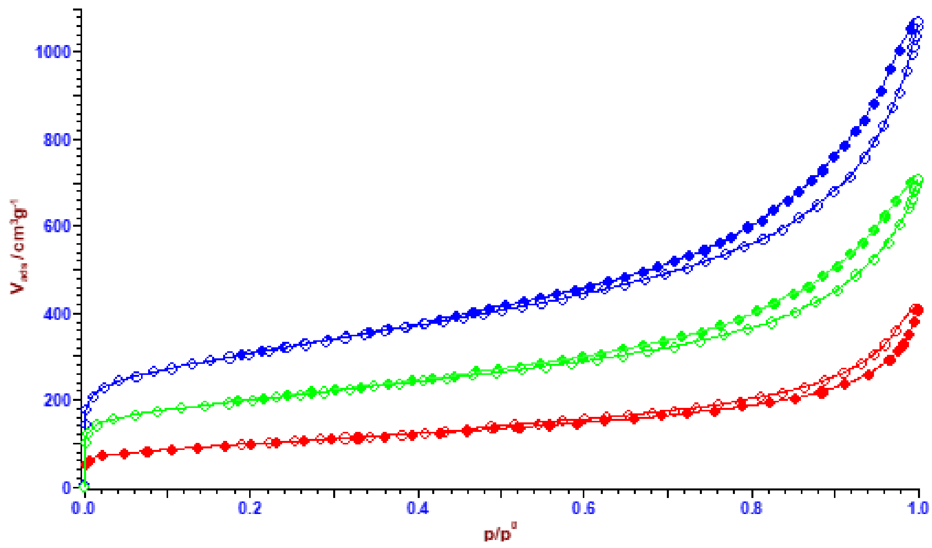

Figure S5. Adsorption isotherms of the parent GNPs, red: GNP-S1; green: GNP-S2, blue: GNP-NG

Table S1. Calculated parameters of parent GNPs based on low temperature N_2 physisorption

Parent GNP	SSA_{BET}, m^2/g	Mesopores surface area, m^2/g	Total pore volume, cm^3/g	Micropore volume, cm^3/g
GNP-NG	700	484	1.08	0.10
GNP-S1	347	268	0.62	0.04
GNP-S2	1068	747	1.64	0.15

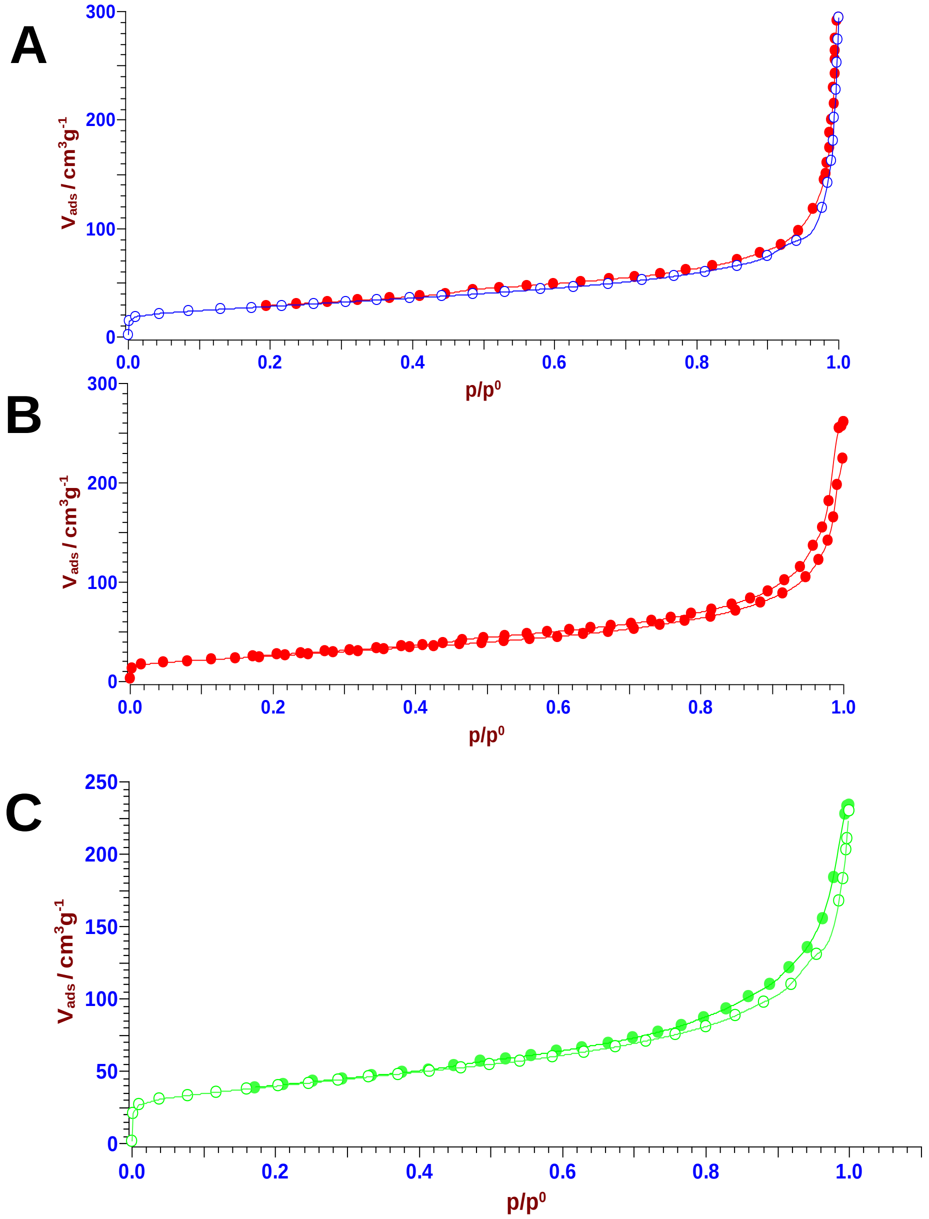

Figure S6. Adsorption isotherms of $Ti_{(x-1)}Mo_xO_2$-C (C: GNP) composites. A: 100GNP-NG [1], B: 100GNP-S1, C: 100GNP-S2 (see denomination of the samples in Table 1 of the main text).

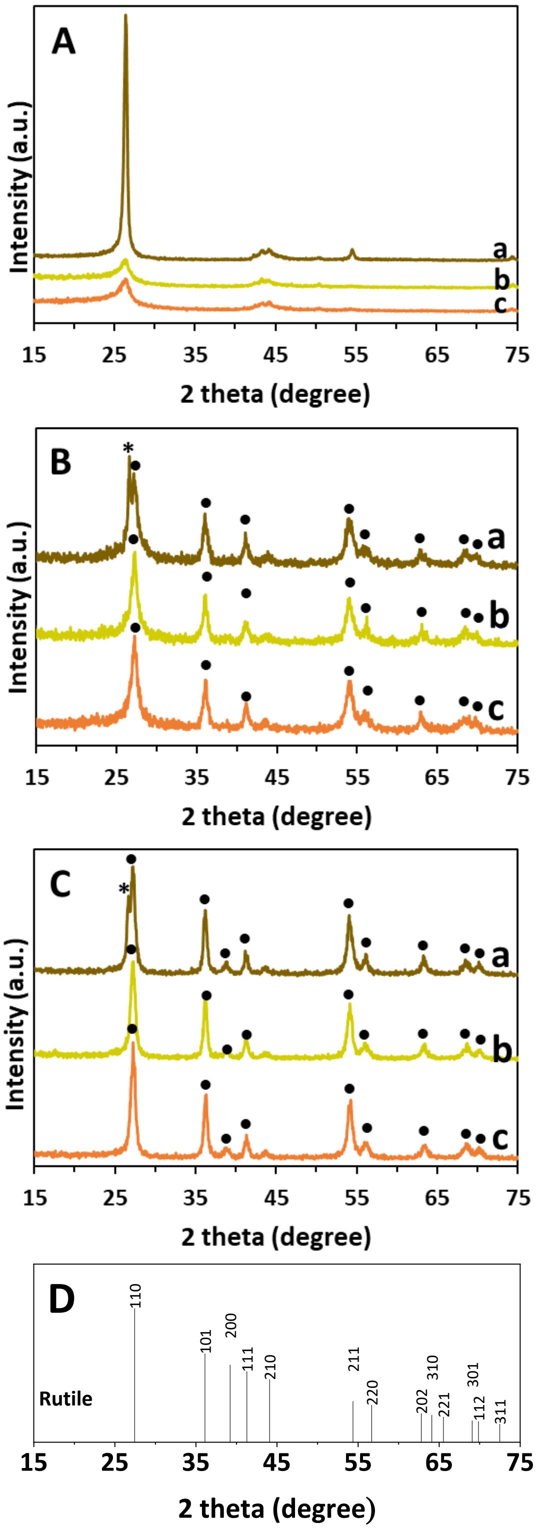

Figure S7. XRD patterns of the composite samples from various type of GNP materials in the different stages of their preparation. A: parent GNP materials, line a: GNP-1S (Sigma-Aldrich No900394, average surface area: 300 m^2/g); line b: GNP-2S (Sigma-Aldrich No900407, average surface area: 750 m^2/g); line c: GNP-NG (Nanografi; SSA_{BET}: 700 m^2/g;), B: Composites before high temperature heat treatment (HTT), line a: 100GNP-1S; line b: 100GNP-2S; line c: 100GNP-NG [1] ●- rutile, *-carbon; C: Composites after HTT, line a: 100GNP-1S; line b: 100GNP-2S; line c: 100GNP-NG [1], ●- rutile, *-carbon, D: rutile TiO_2 (JCPDS card no. 21-1276)

S3.2. Results obtained by use of mixtures GNP-S2 and GO

S3.2.1. Low temperature N_2 physisorption

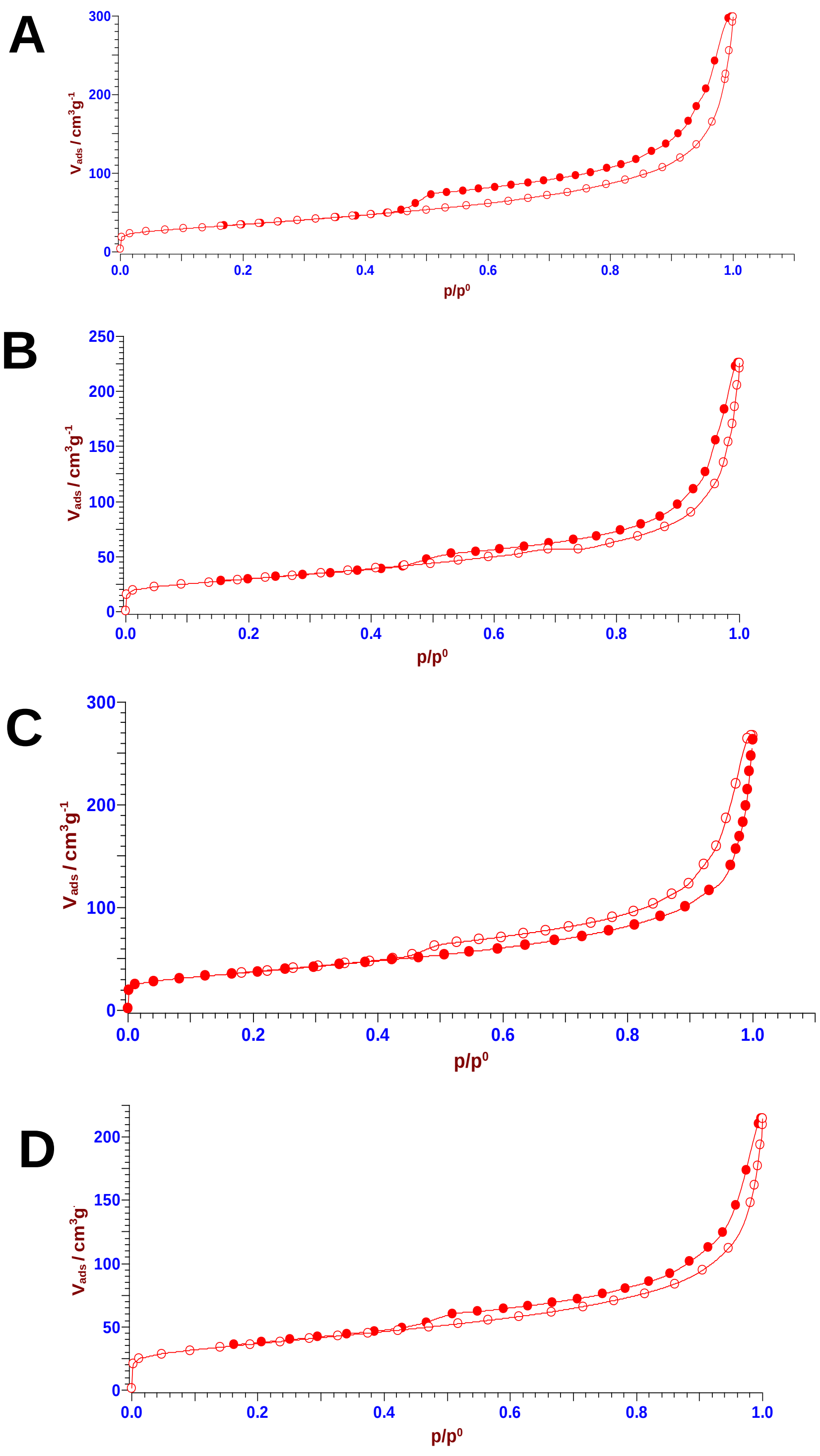

Figure S8. Adsorption isotherms of $Ti_{(x-1)}Mo_xO_2$-C (C: GNP-GO mixture derived carbon) composites. A: 75GNP-S2; B: 50GNP-S2; C: 50GNP-S2R; D: 25GNP-S2 (see composition of the samples in Table 1 of the main text).

S3.2.2 Results of thermal analysis

Table S2. Data of the thermal analysis of parent GNP-S2

**Type of curves**	**as received**
Mass loss (TG) and heat flow	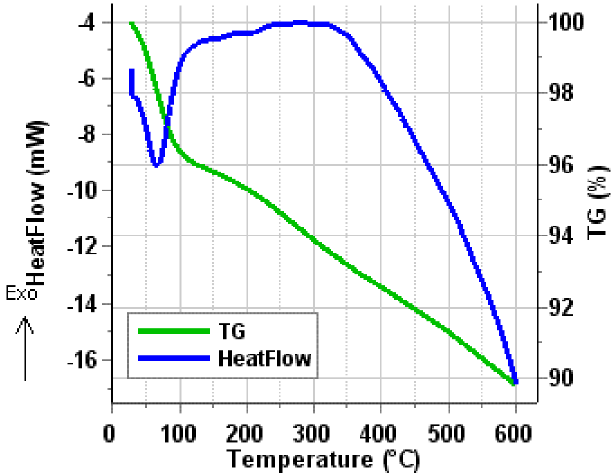

Mass loss (TG) and mass loss rate (dTG)	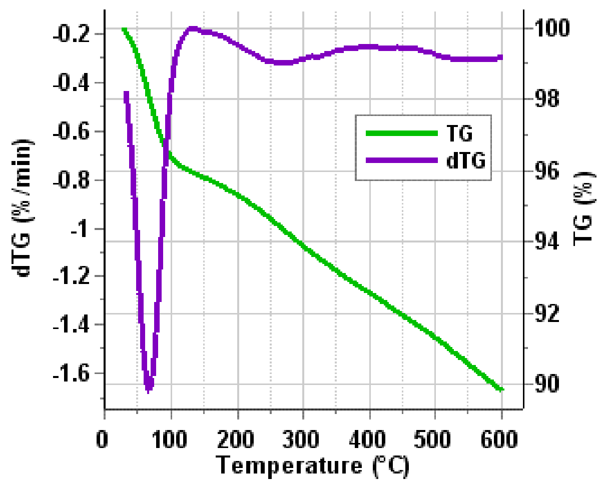
Quantitatively evaluated mass loss	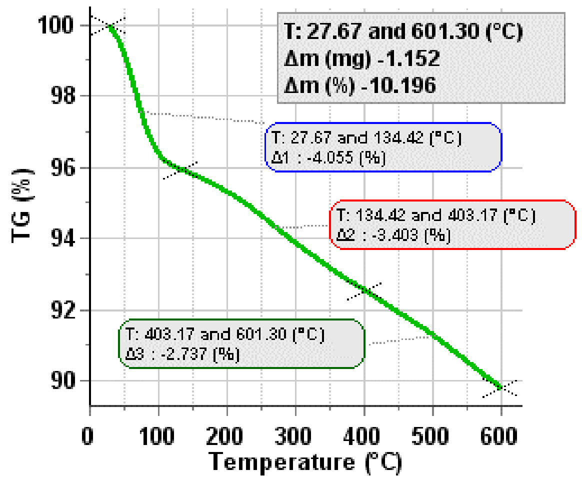

Quantitatively evaluated heat flow	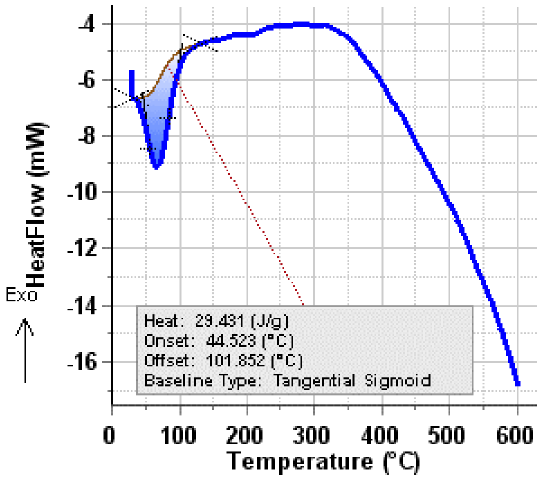
Ion current of the CO_2 and its fragments	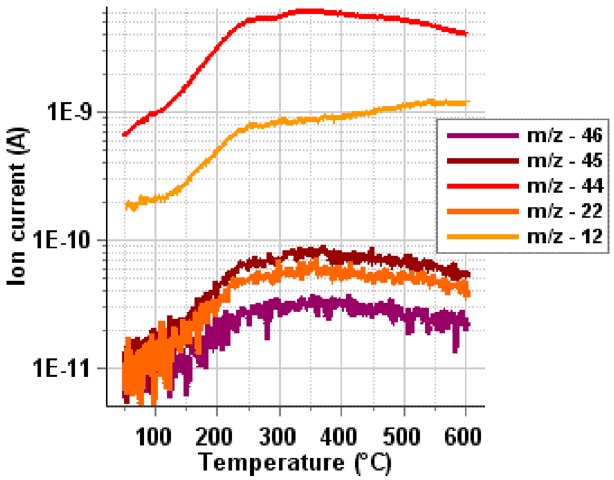

<table>
<tr>
<td>Ion current of the CO and its fragments</td>
<td>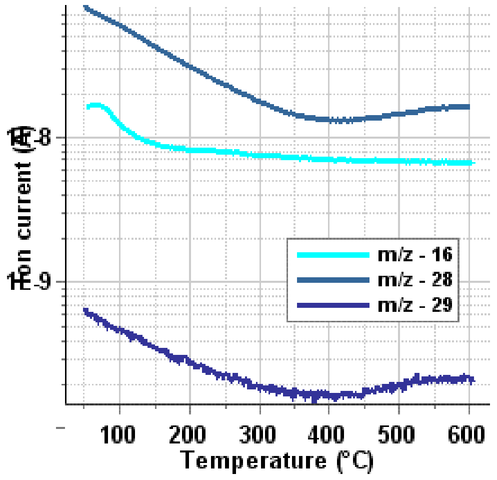
</td>
</tr>
<tr>
<td>Water fragments</td>
<td>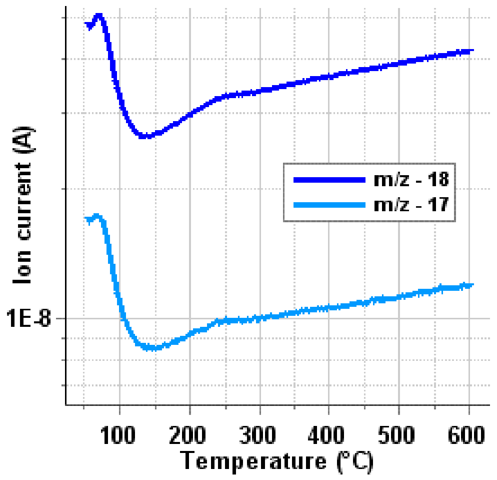
</td>
</tr>
</table>

Table S3. Data of the thermal analysis of sample 100GNP-S2

**Type of curves**	**Before HTT (dried at 80°C)**	**After HTT (600°C in Ar)**
Mass loss (TG) and heat flow	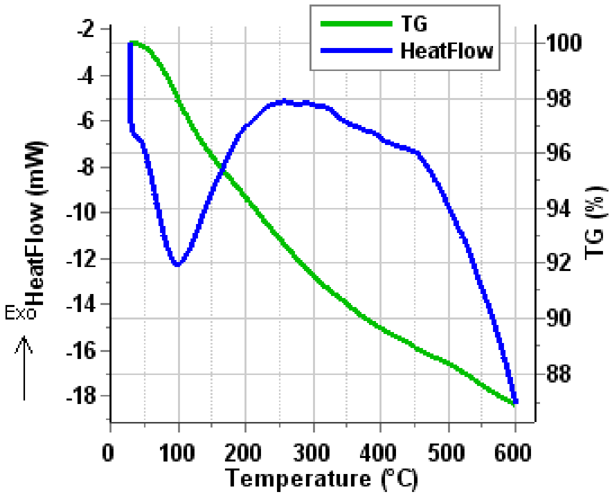	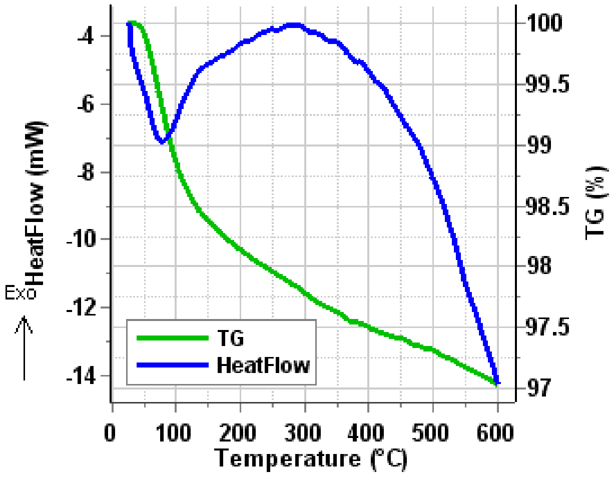

Mass loss (TG) and mass loss rate (dTG)	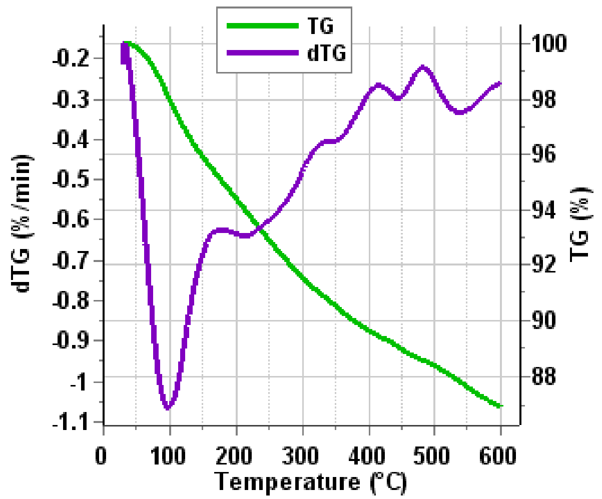	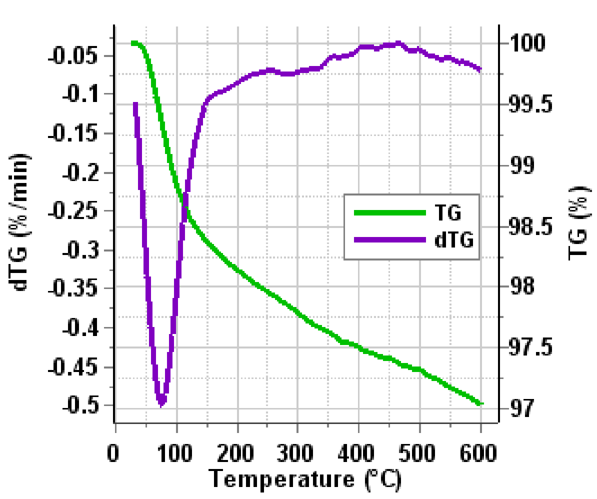
Quantitatively evaluated mass loss	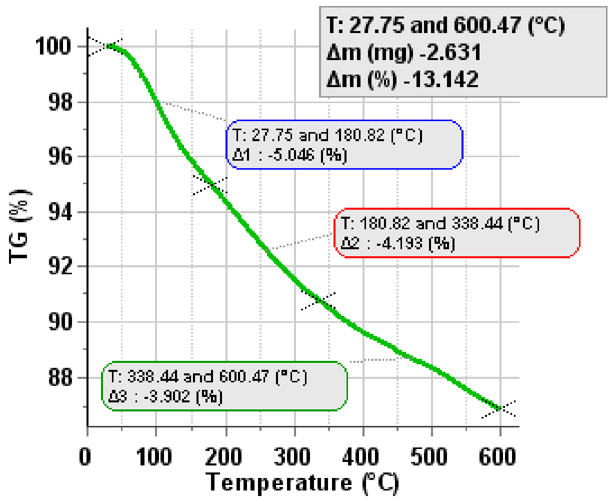	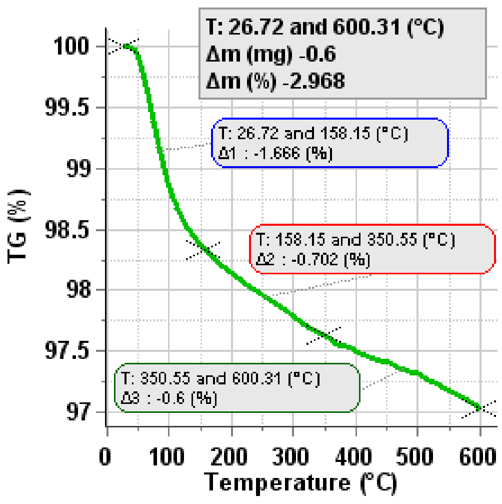

Quantitatively evaluated heat flow	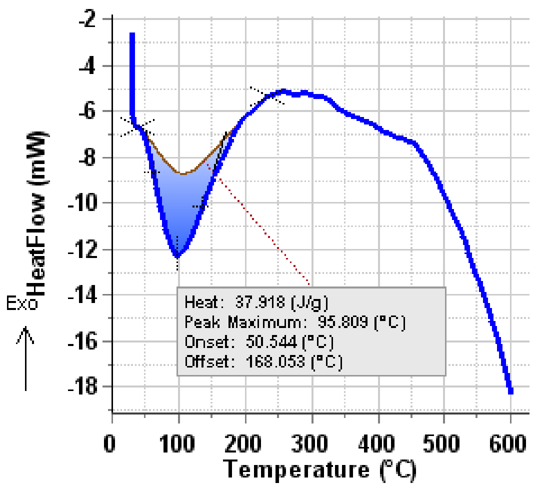	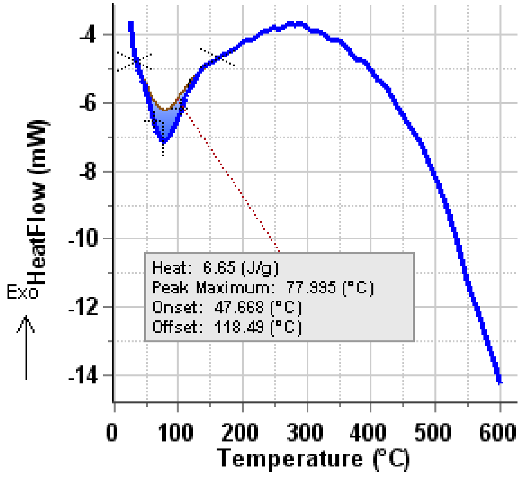
Ion current of the CO_2 and its fragments	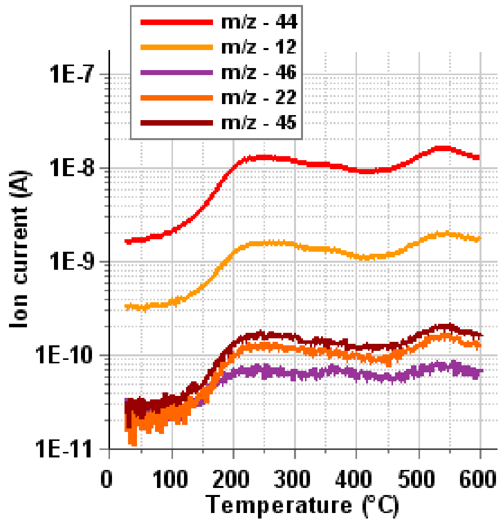	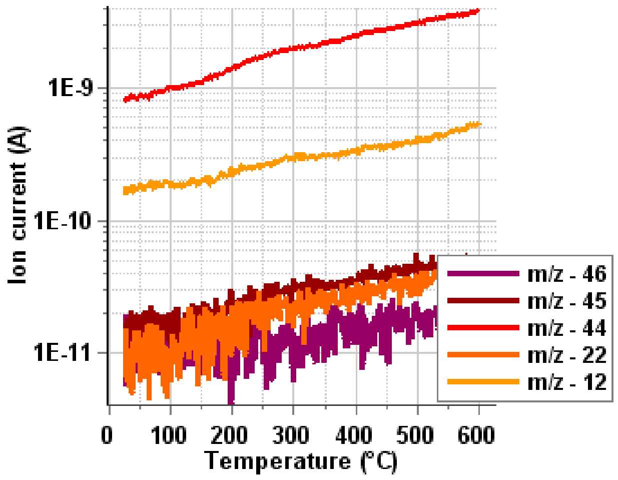

Ion current of the CO and its fragments	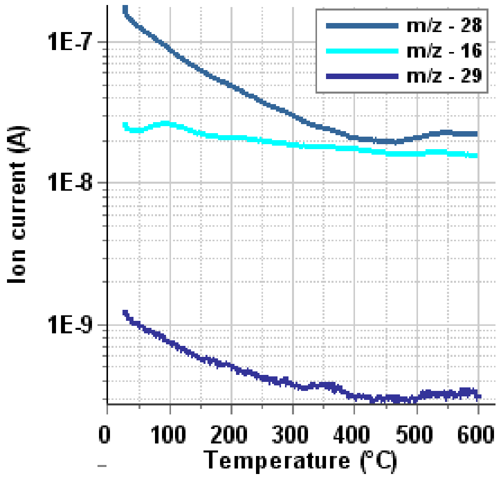	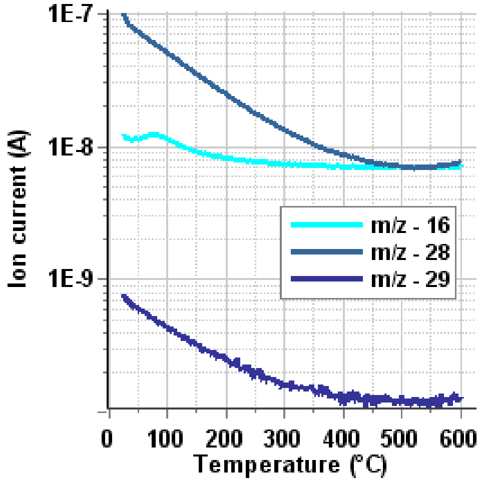
Ion current of the formaldehyde's molecular ion	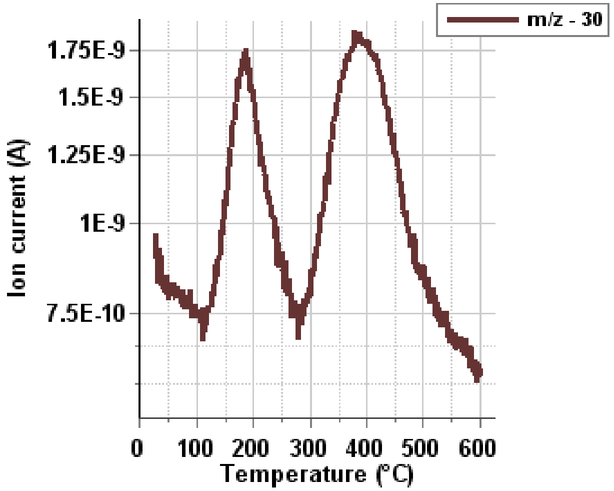	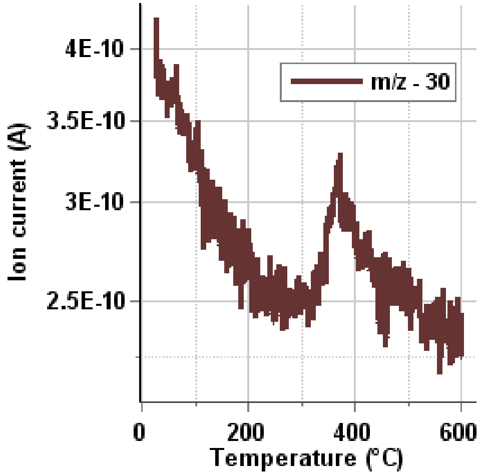

Ion current of the hydrogen cyanide	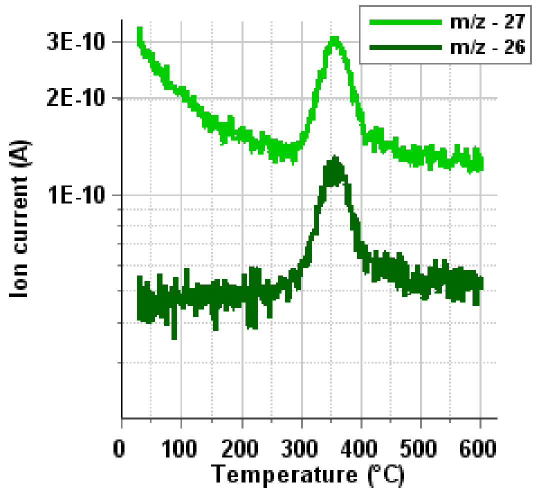	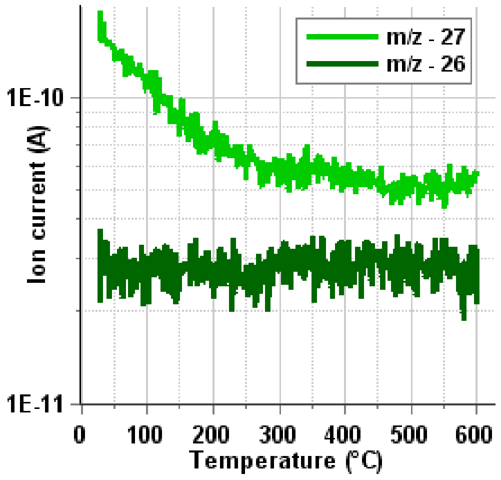
Water fragments	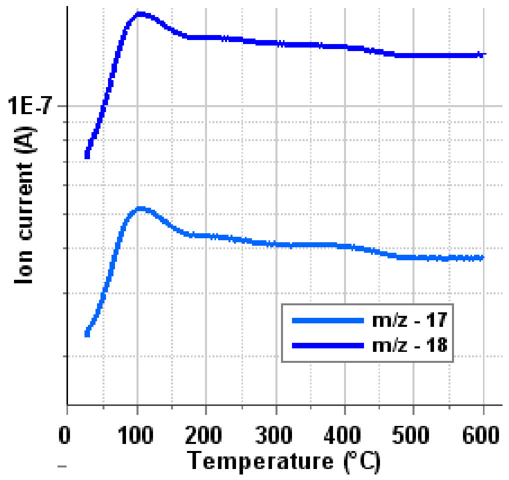	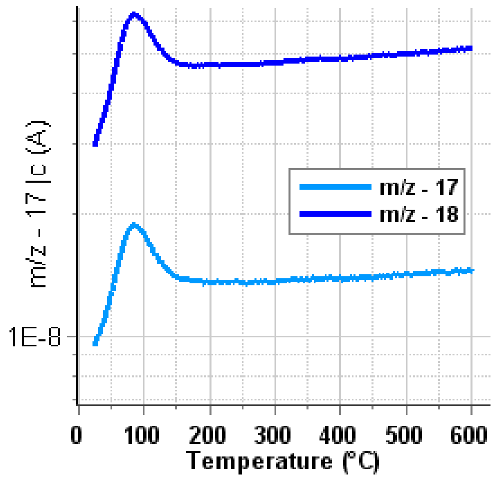

Other fragments	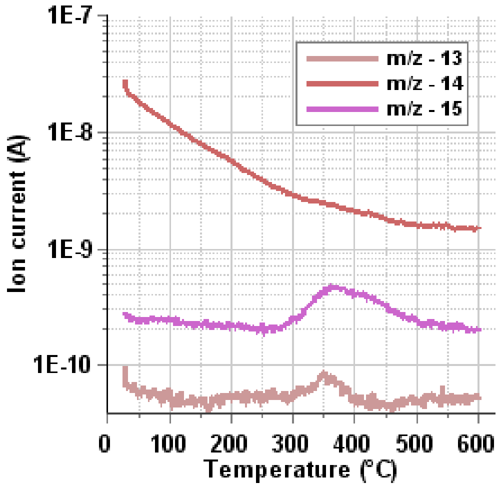	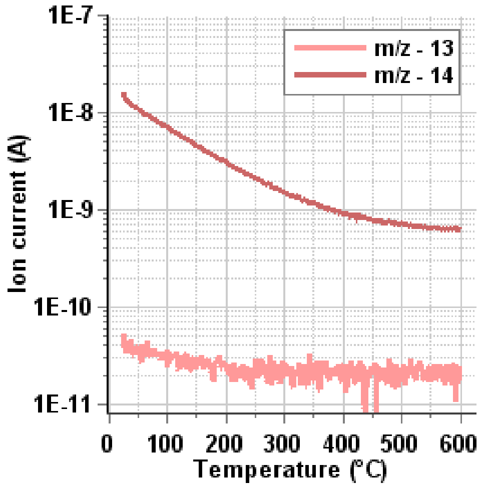
Isopropoxide fragments	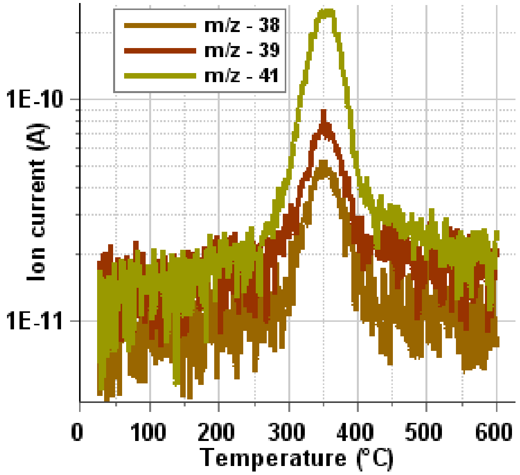	

Sulfur dioxide fragments	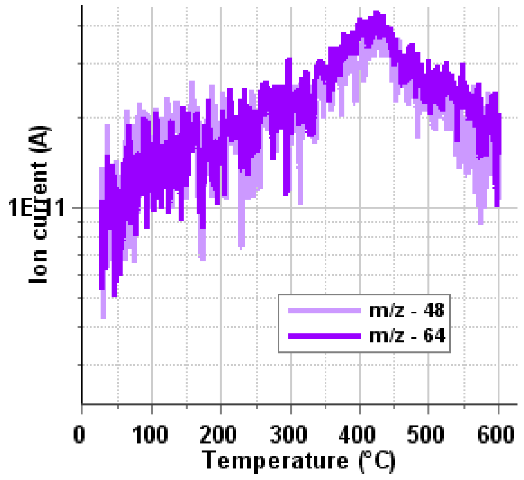	

Table S4. Data of the thermal analysis of sample 75GNP-S2

**Type of curves**	**Before HTT (dried at 80°C)**	**After HTT (600°C in Ar)**
Mass loss (TG) and heat flow	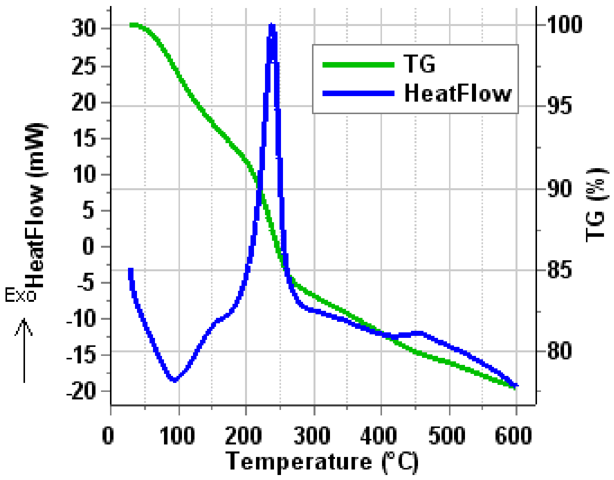	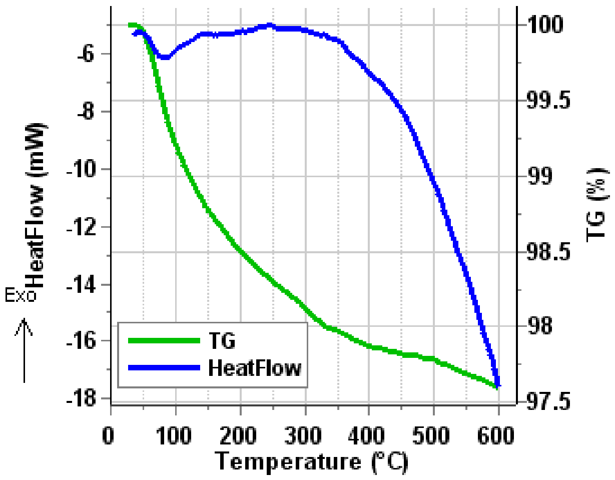

<table>
<tr>
<td>Mass loss (TG) and mass loss rate (dTG)</td>
<td>
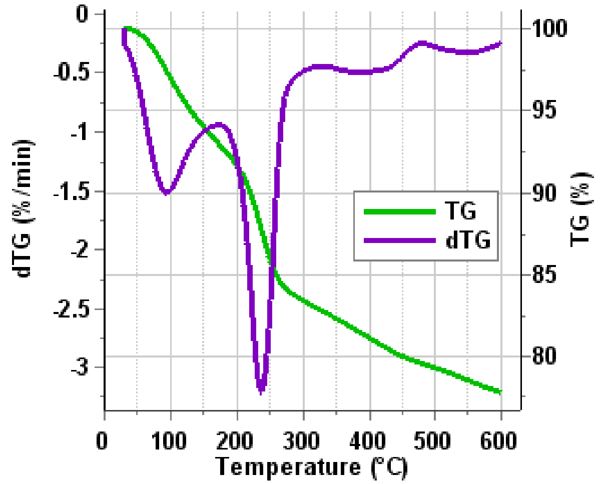

</td>
<td>
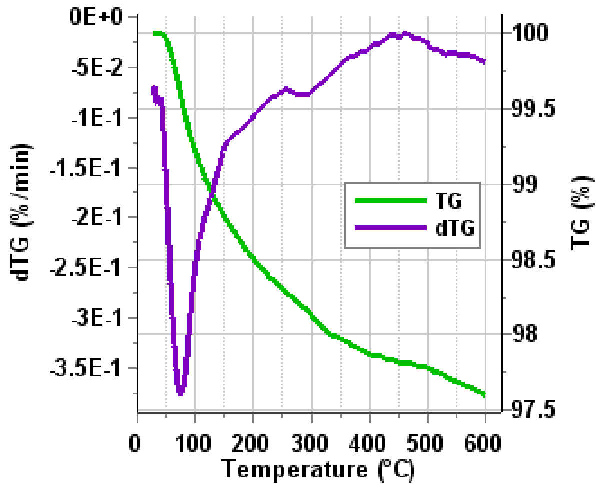

</td>
</tr>
<tr>
<td>Quantitatively evaluated mass loss</td>
<td>
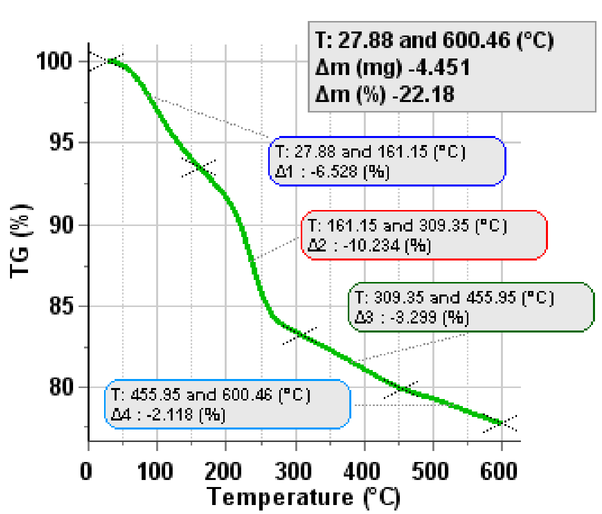

</td>
<td>
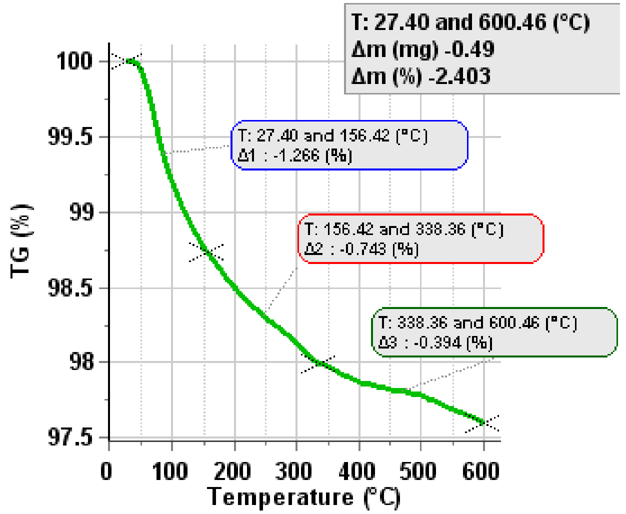

</td>
</tr>
</table>

<table>
<tr>
<td>Quantitatively evaluated heat flow</td>
<td>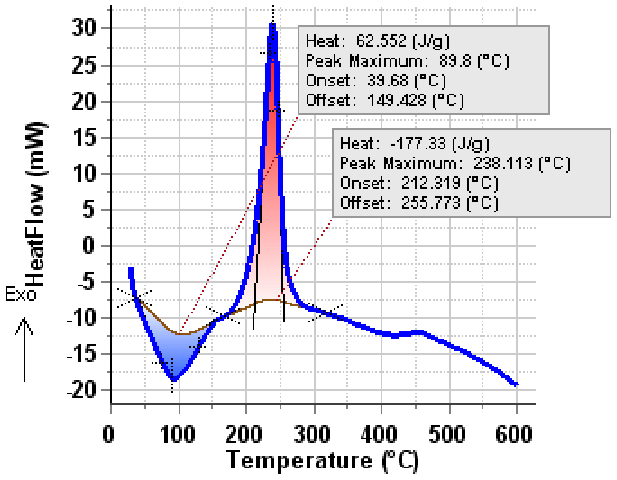
</td>
<td>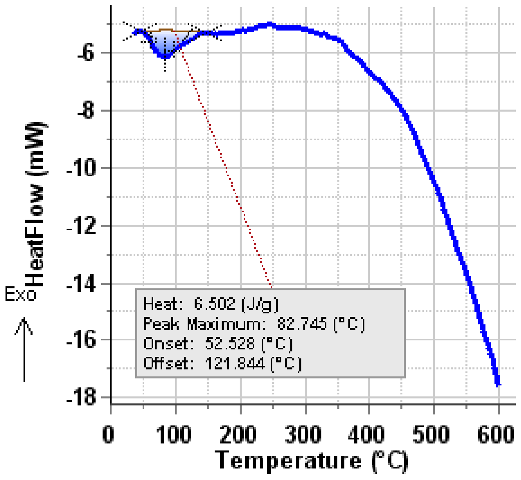
</td>
</tr>
<tr>
<td>Ion current of the CO_2 and its fragments</td>
<td>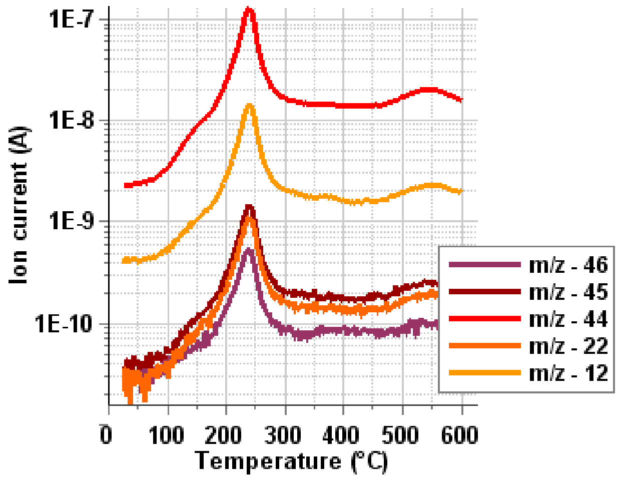
</td>
<td>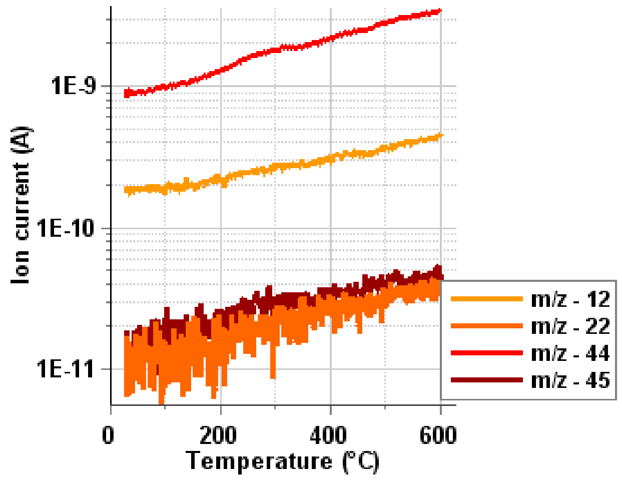
</td>
</tr>
</table>

<table>
<tr>
<td>Ion current of the CO and its fragments</td>
<td>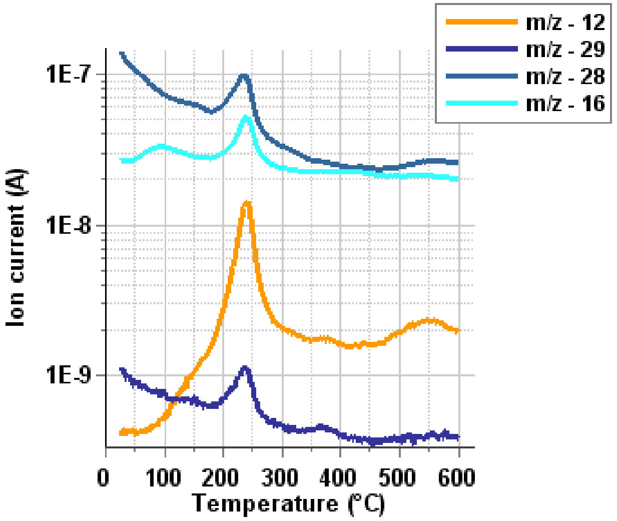
</td>
<td>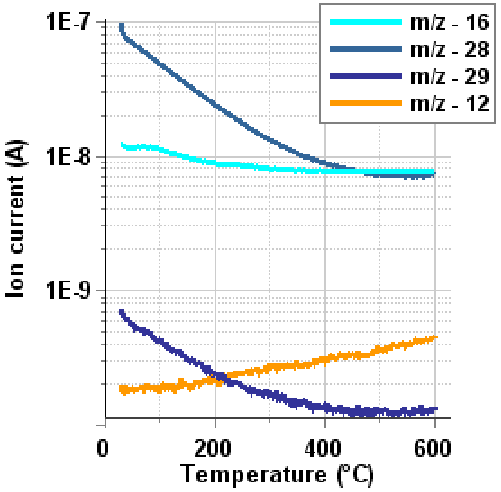
</td>
</tr>
<tr>
<td>Ion current of the formaldehyde’s molecular ion</td>
<td>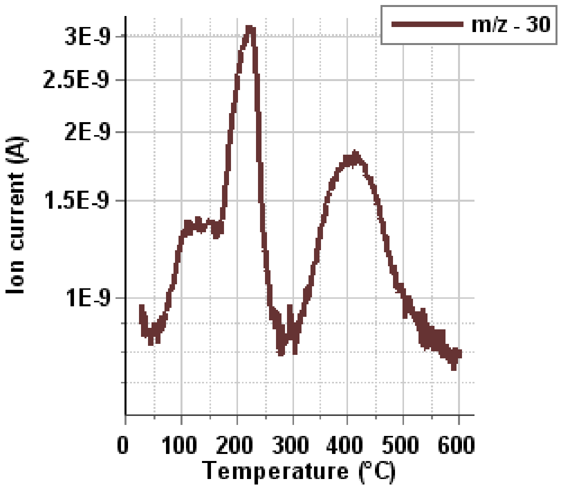
</td>
<td>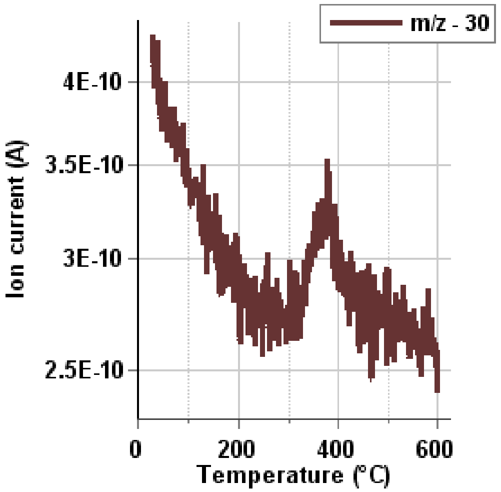
</td>
</tr>
</table>

Ion current of the hydrogen cyanide	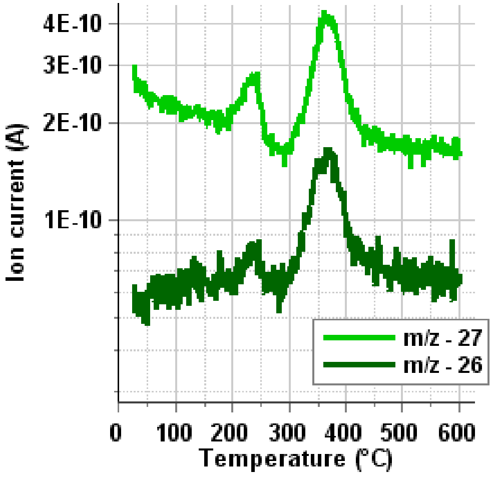	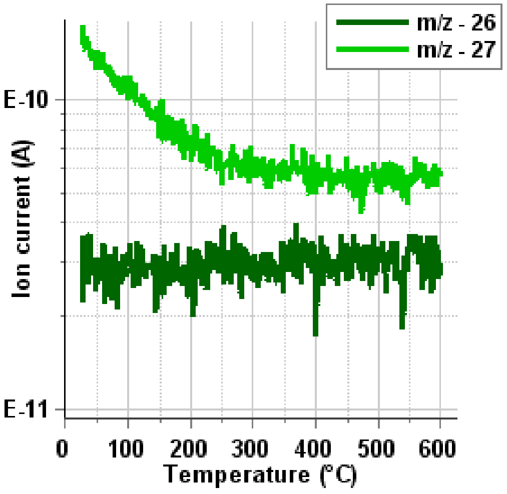
Water fragments	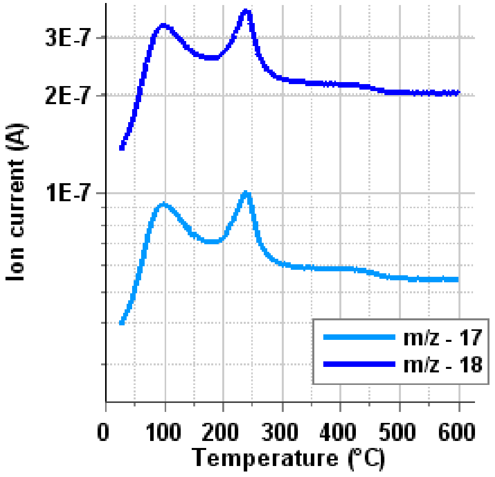	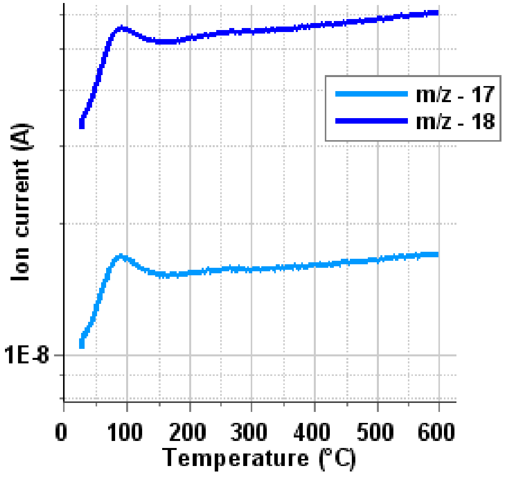

<table>
<tr>
<td>Other fragments</td>
<td>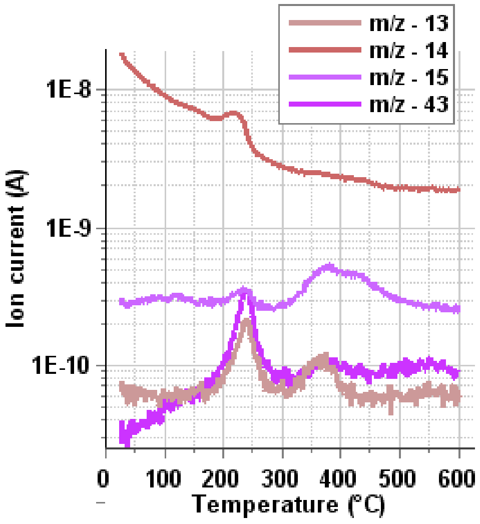</td>
<td>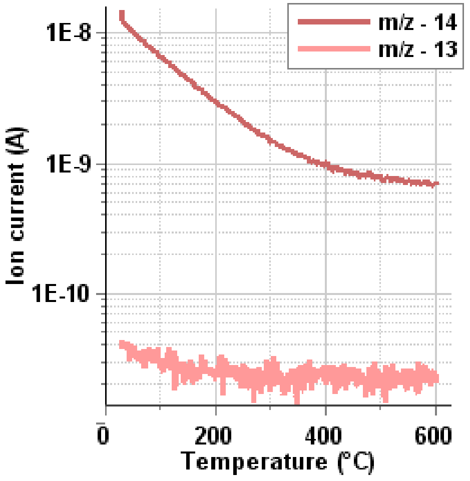</td>
</tr>
<tr>
<td>Isopropoxide fragments</td>
<td>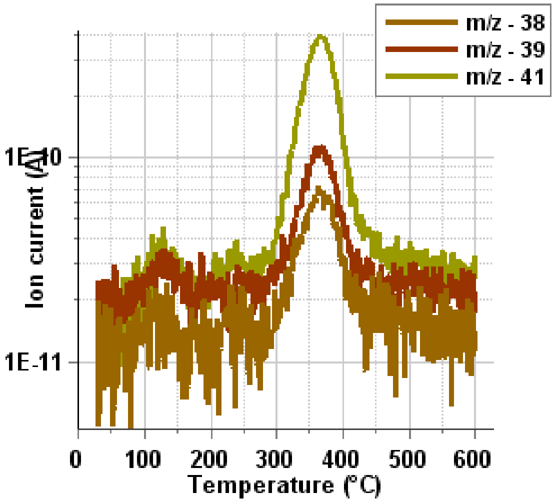</td>
<td></td>
</tr>
</table>

Sulfur dioxide fragments	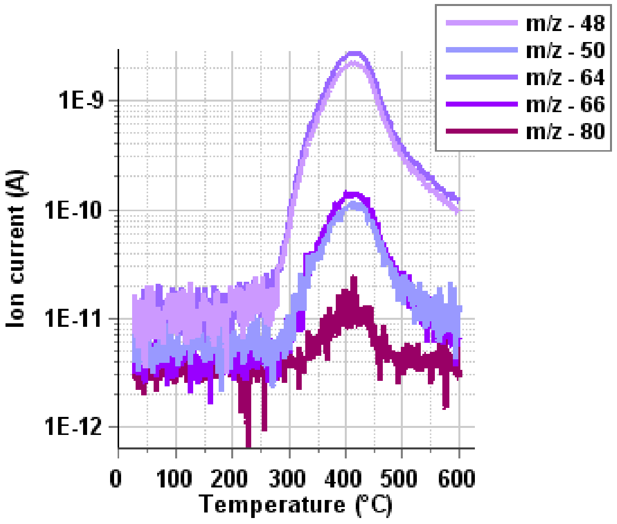	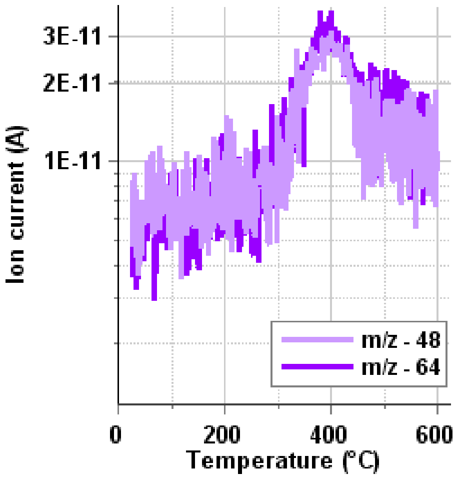

Table S5. Data of the thermal analysis of sample 50GNP-S2

**Type of curves**	**Before HTT (dried at 80°C)**	**After HTT (600°C in Ar)**
Mass loss (TG) and heat flow	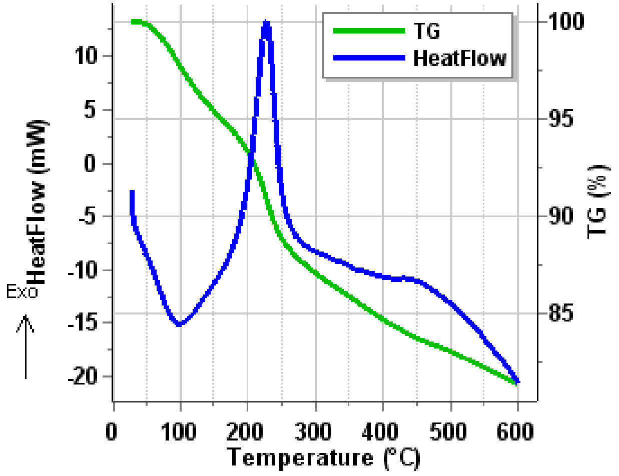	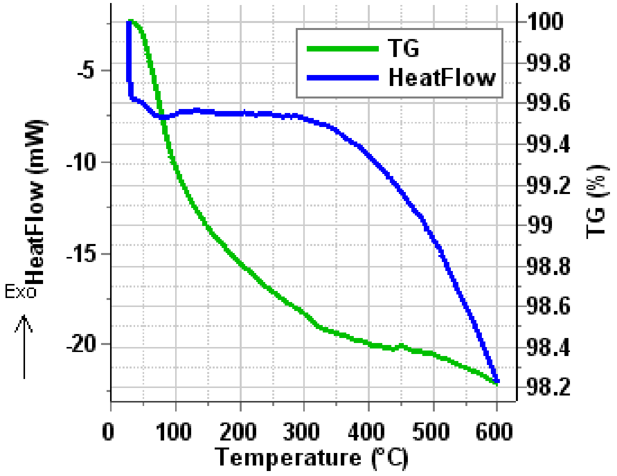

Mass loss (TG) and mass loss rate (dTG)	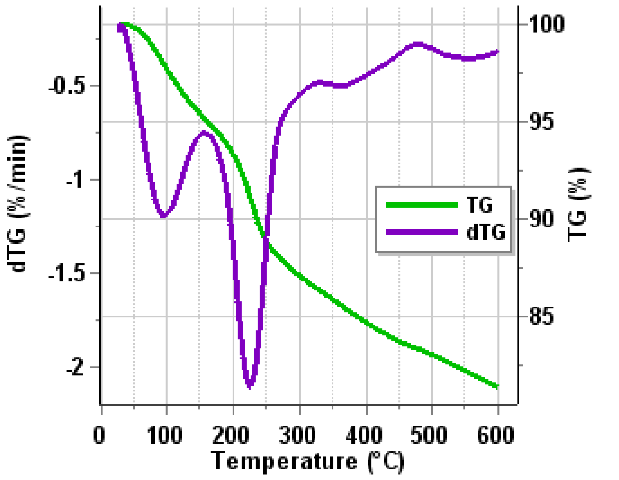	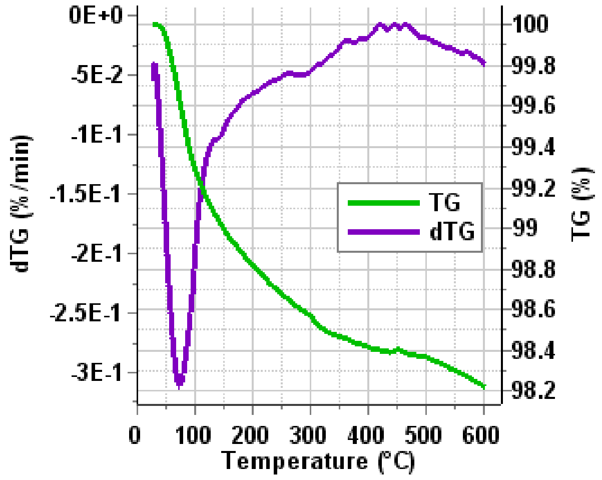
Quantitatively evaluated mass loss	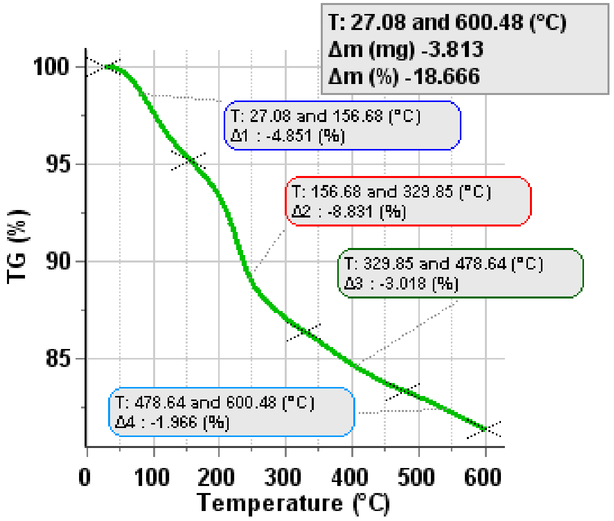	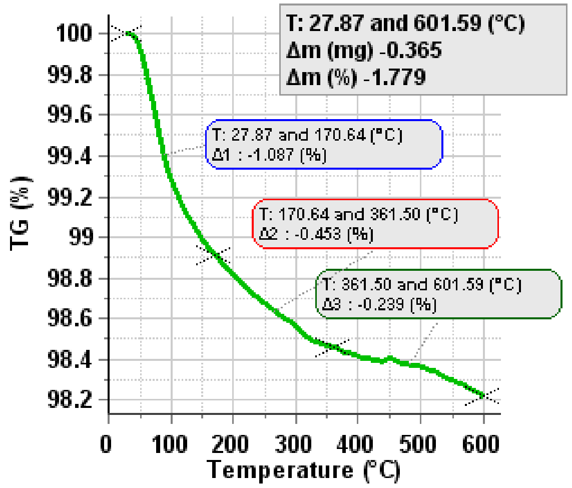

<table>
<tr>
<td>Quantitatively evaluated heat flow</td>
<td>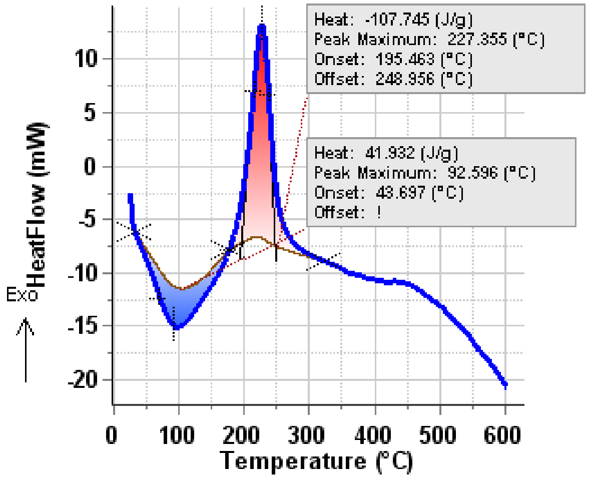
</td>
<td>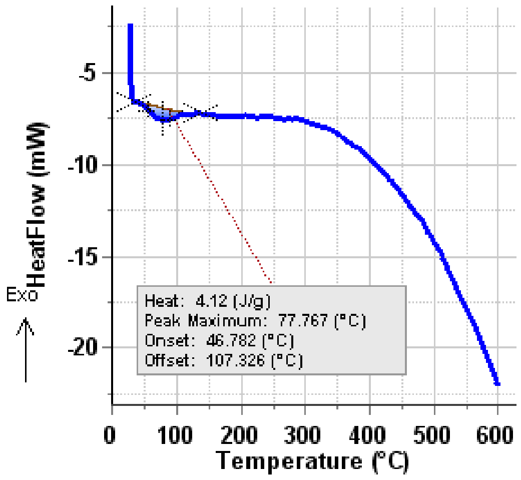
</td>
</tr>
<tr>
<td>Ion current of the CO_2 and its fragments</td>
<td>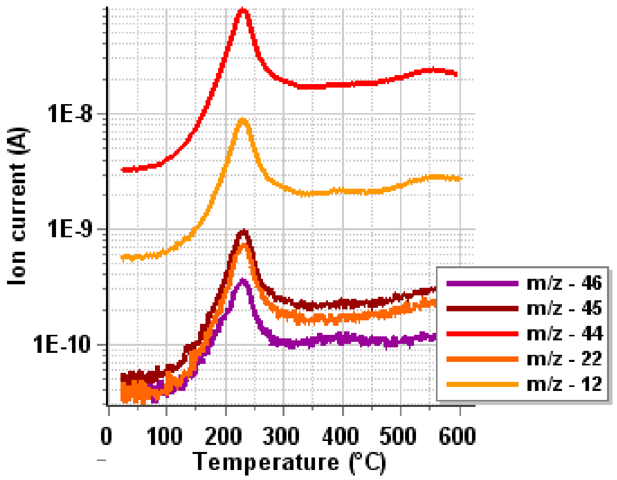
</td>
<td>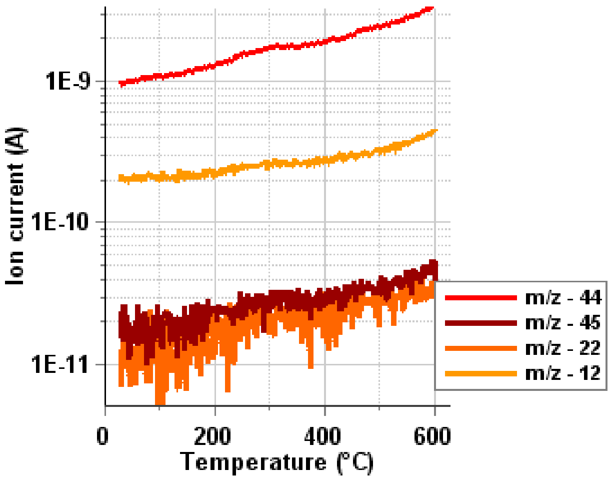
</td>
</tr>
</table>

<table>
<tr>
<td>Ion current of the CO and its fragments</td>
<td>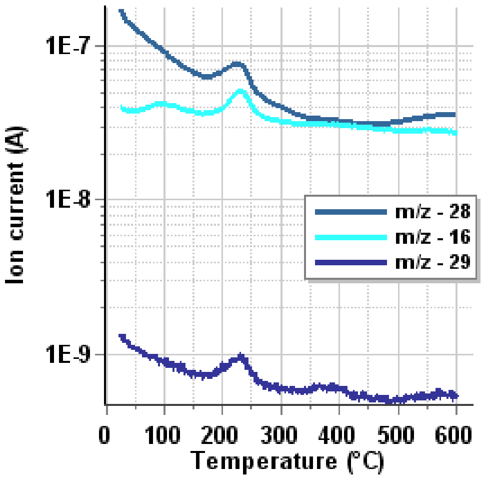
</td>
<td>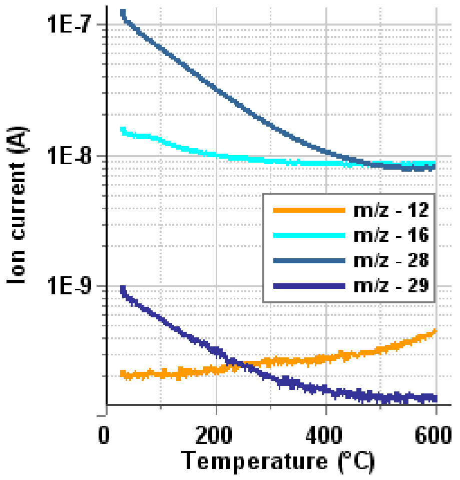
</td>
</tr>
<tr>
<td>Ion current of the formaldehyde's molecular ion</td>
<td>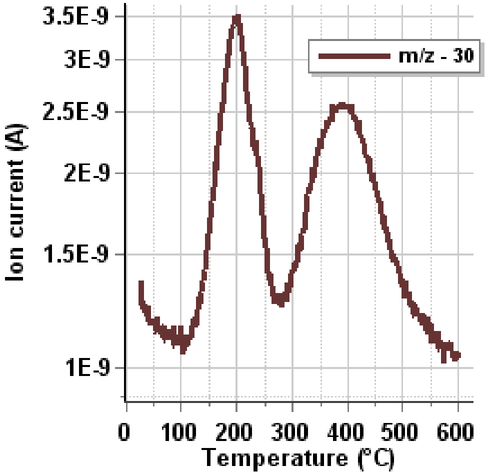
</td>
<td>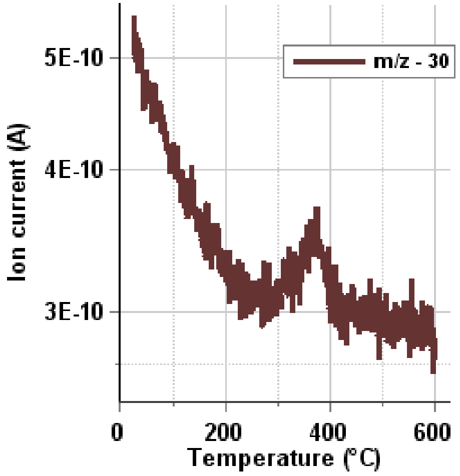
</td>
</tr>
</table>

Ion current of the hydrogen cyanide	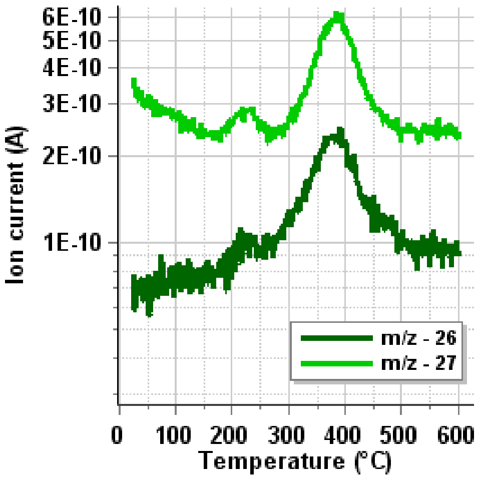	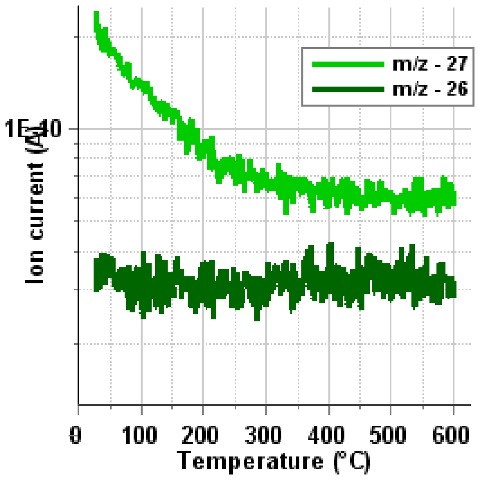
Water fragments	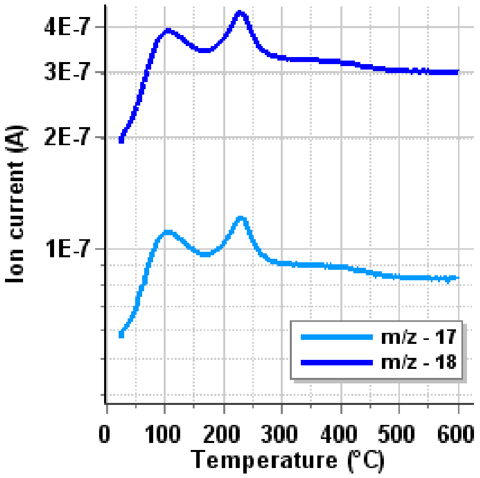	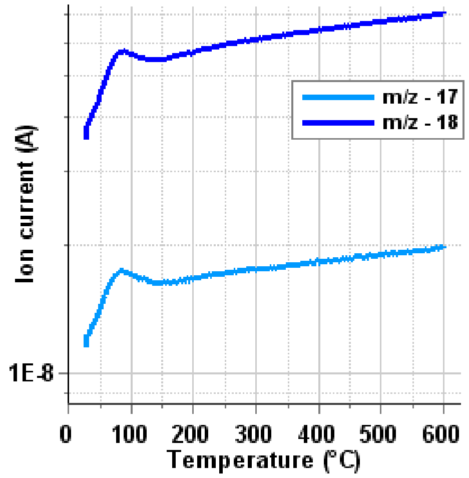

Other fragments	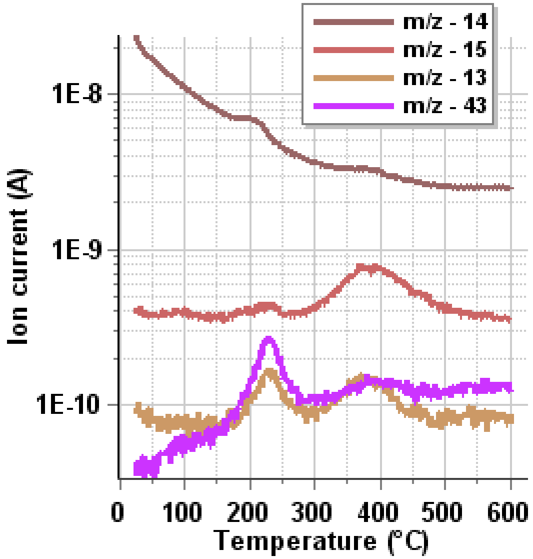	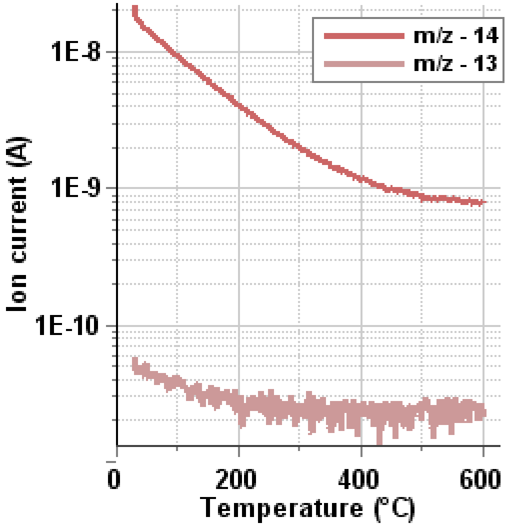

Isopropoxide fragments	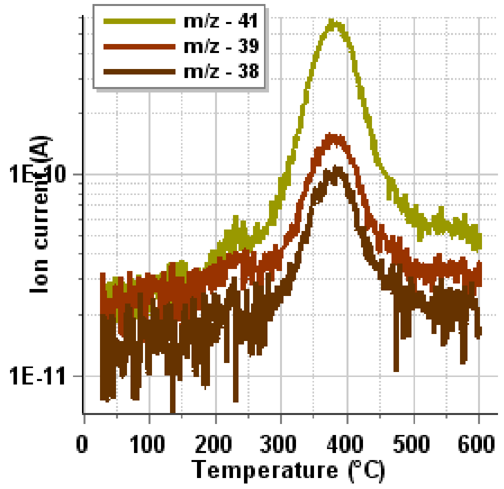	
Sulfur dioxide fragments	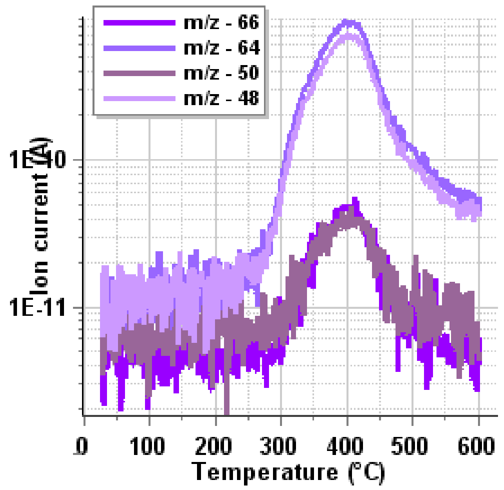	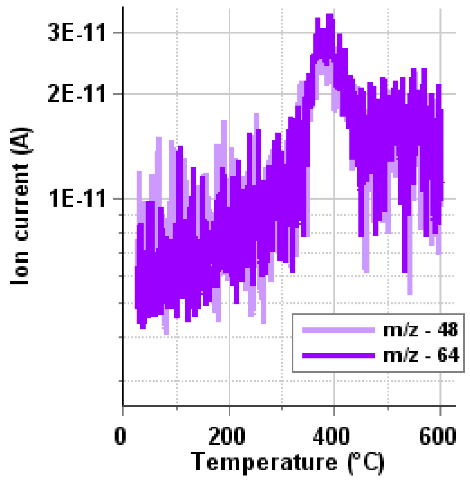

Table S6. Data of the thermal analysis of sample 25GNP-S2

**Type of curves**	**Before HTT (dried at 80°C)**	**After HTT (600°C in Ar)**
Mass loss (TG) and heat flow	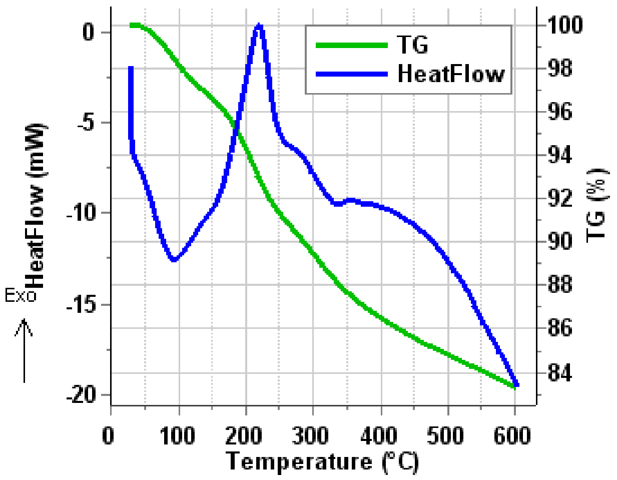	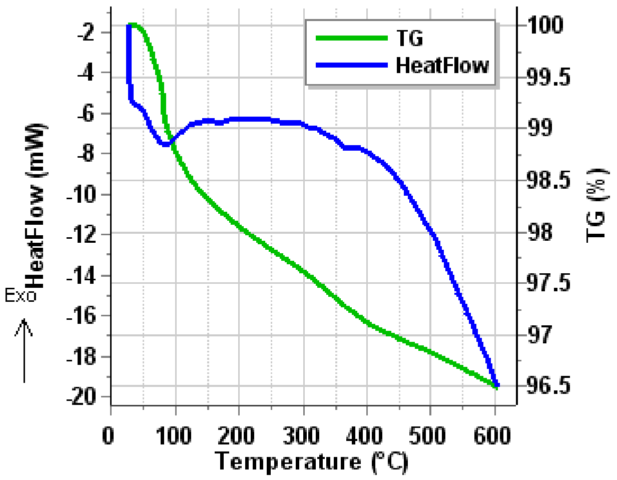

<table>
<tr>
<td>Mass loss (TG) and mass loss rate (dTG)</td>
<td>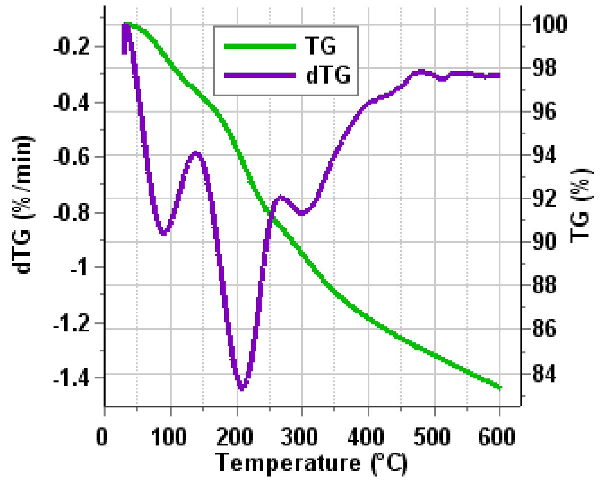
</td>
<td>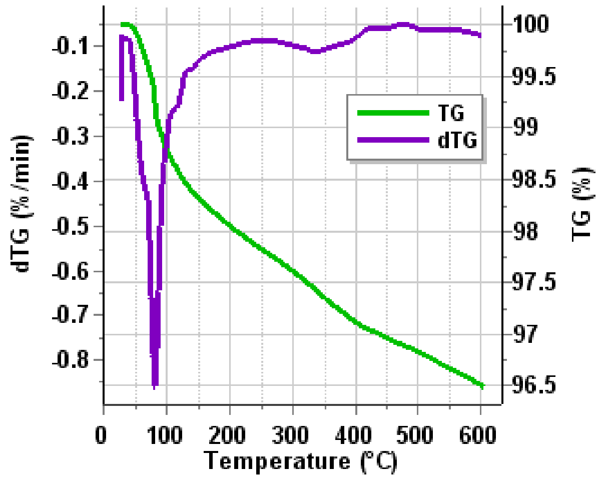
</td>
</tr>
<tr>
<td>Quantitatively evaluated mass loss</td>
<td>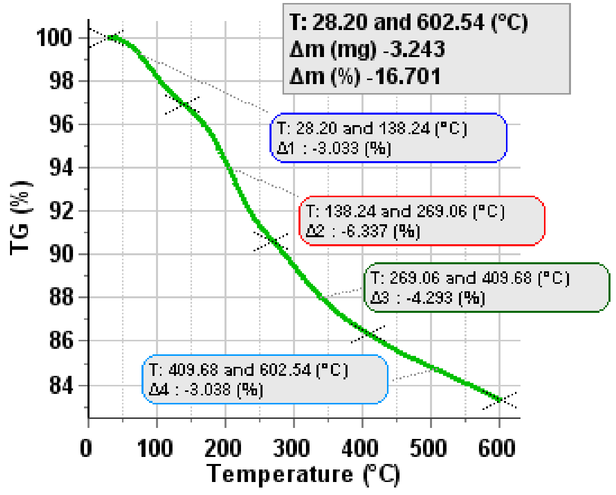
</td>
<td>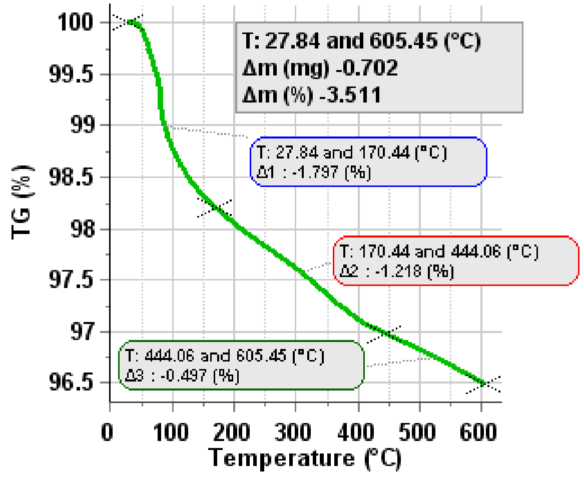
</td>
</tr>
</table>

<table>
<tr>
<td>Quantitatively evaluated heat flow</td>
<td>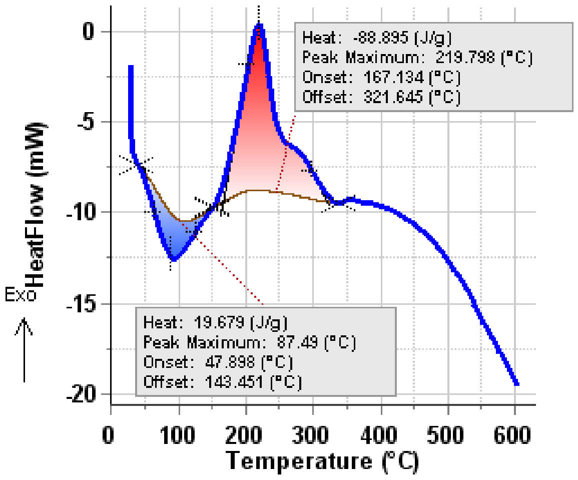
</td>
<td>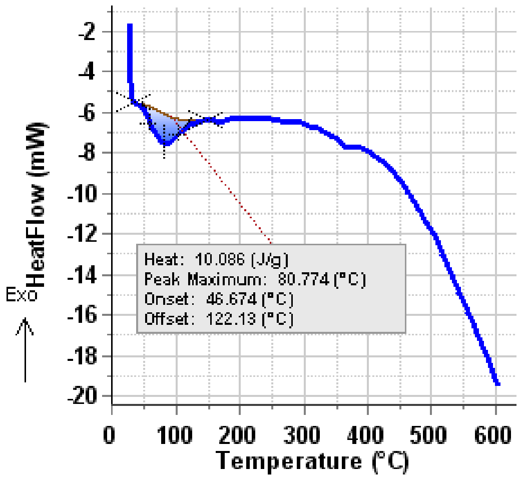
</td>
</tr>
<tr>
<td>Ion current of the CO_2 and its fragments</td>
<td>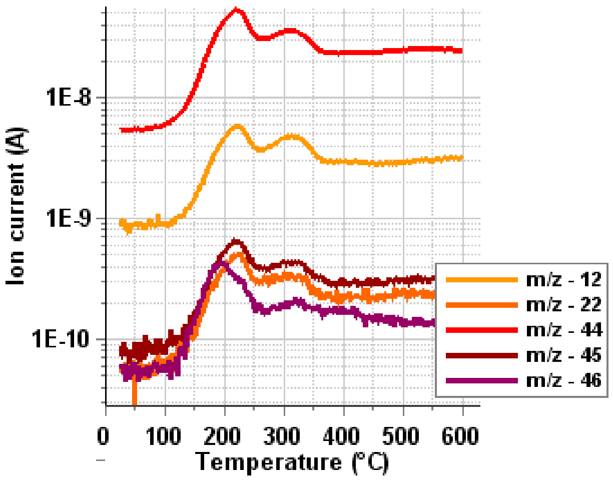
</td>
<td>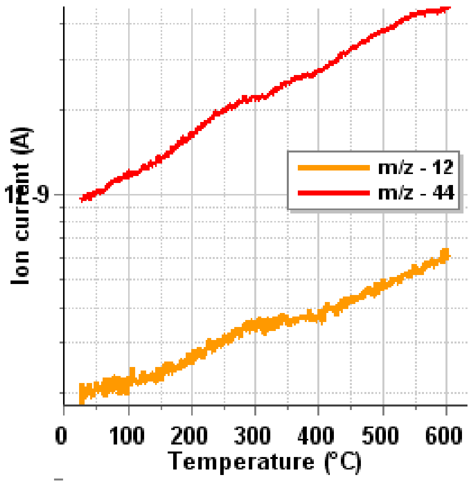
</td>
</tr>
</table>

Ion current of the CO and its fragments	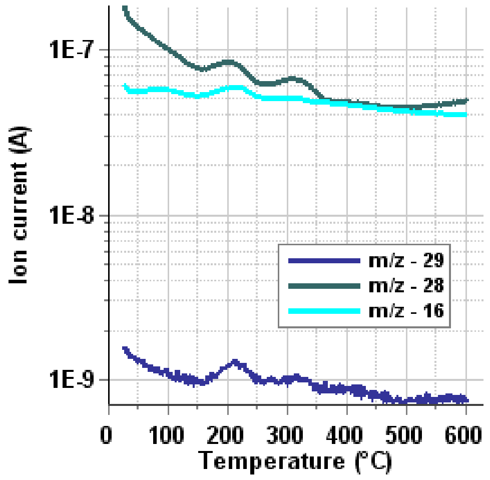	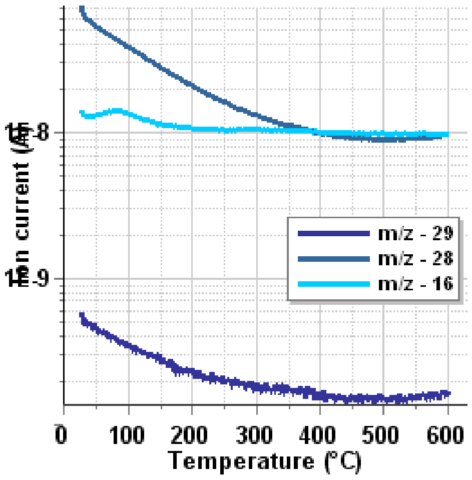
Ion current of the formaldehyde's molecular ion	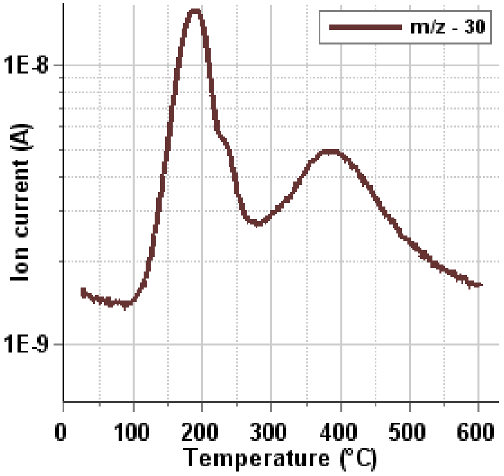	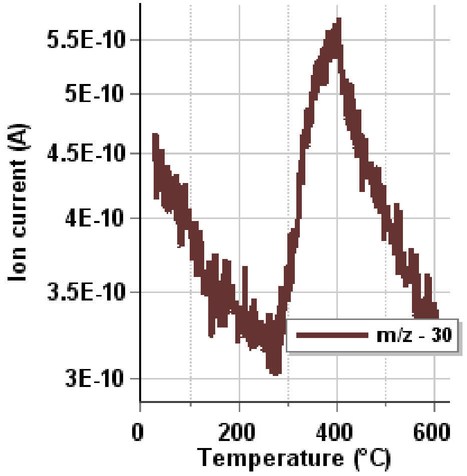

<table>
<tr>
<td>Ion current of the hydrogen cyanide</td>
<td>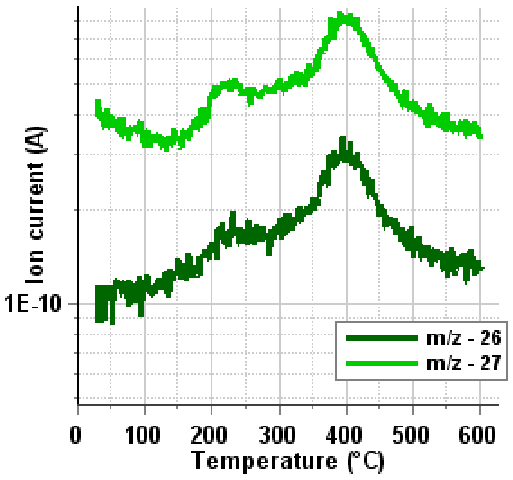
</td>
<td>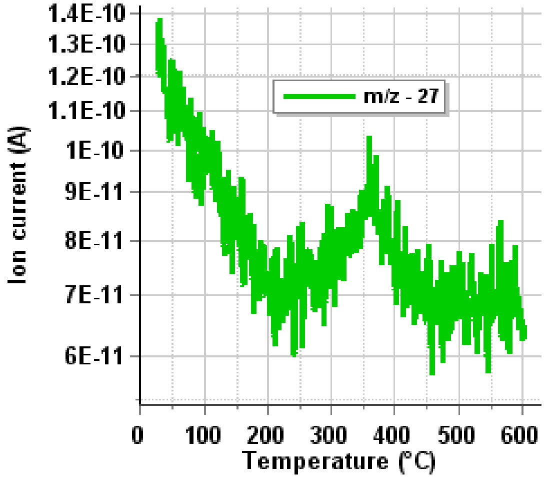
</td>
</tr>
<tr>
<td>Water fragments</td>
<td>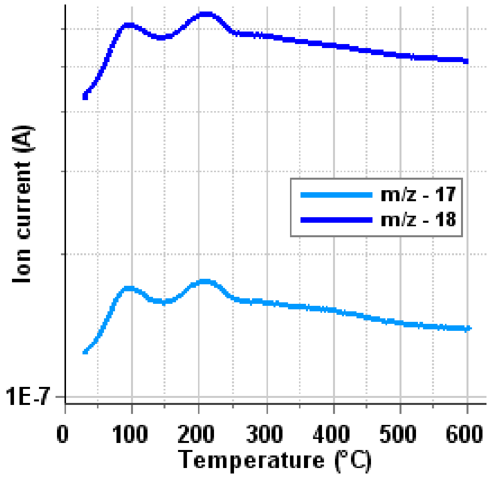
</td>
<td>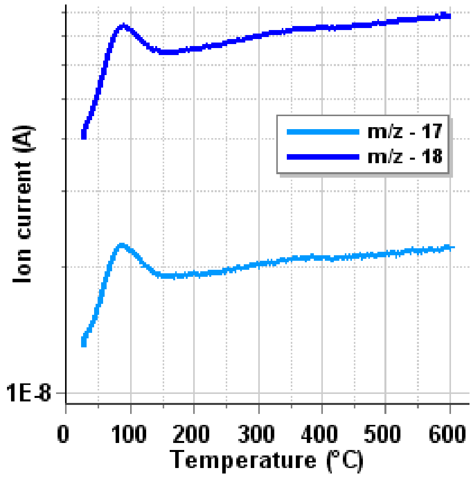
</td>
</tr>
</table>

Other fragments	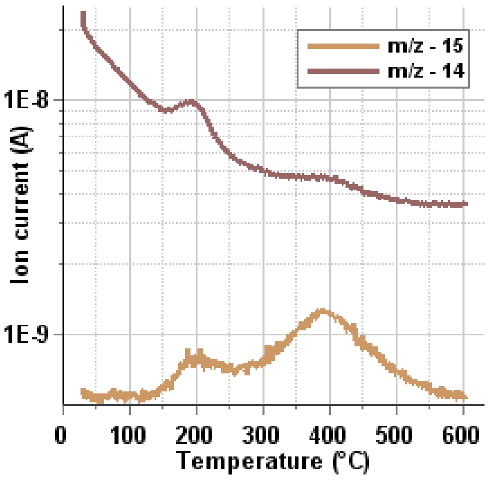	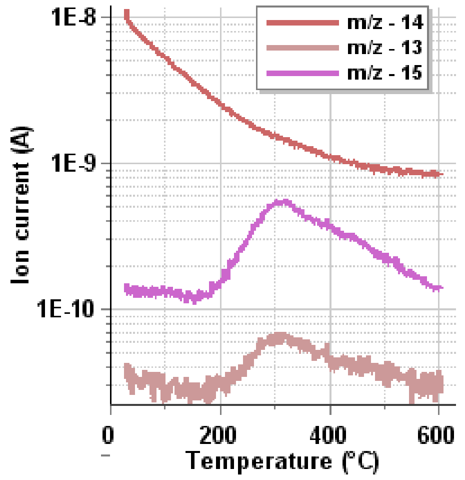
Isopropoxide fragments	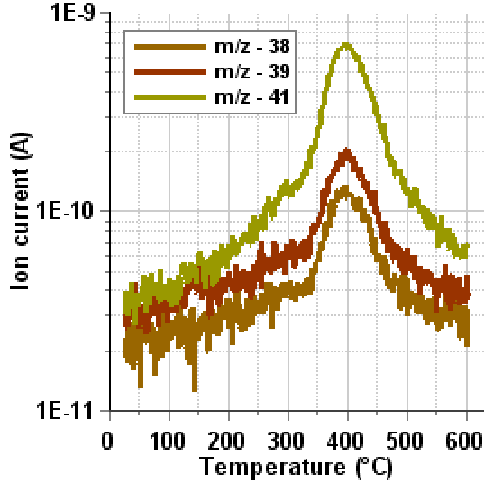	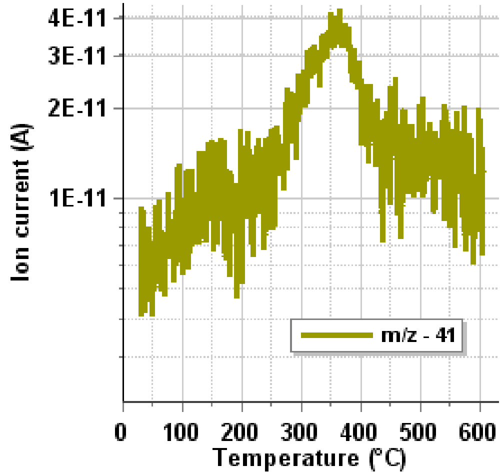

Sulfur dioxide fragments	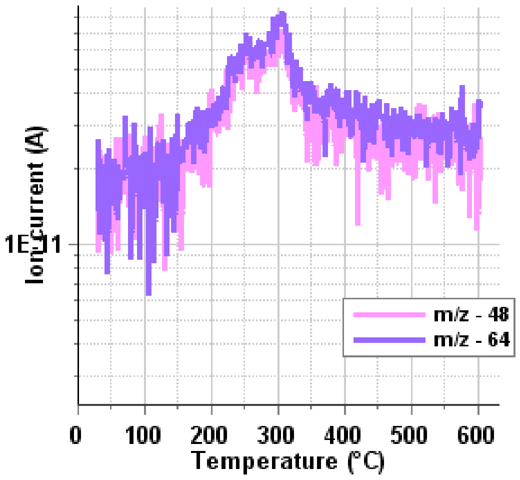	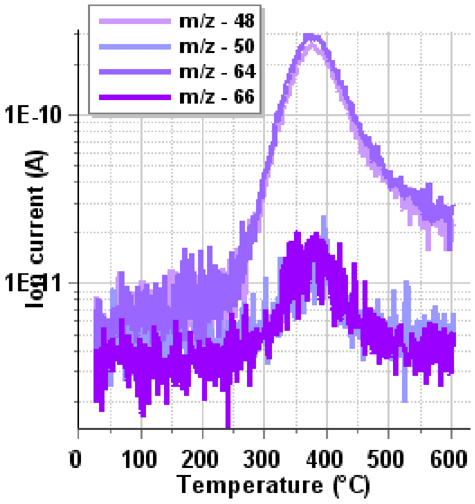

S3.2.3 Results of XPS measurements

Table S7. XPS results of 20 wt.% Pt/$Ti_{(1-x)}Mo_xO_2$-C [1] catalysts: Pt/100GNP-S2, Pt/75GNP-S2, Pt/50GNP-S2 and Pt/25GNP-S2.

**Sample ID**	**Composition**	**Chemical state**				
	**(in at%)**	**BE [2] (ratio)**	**BE [2] assign.**	**BE [2] assign.**	**BE [2] assign.**	**BE [2] (ratio)**
		**Mo 3d**	**Ti 2p**	**O 1s**	**C 1s**	
Pt/100GNP-S2	Mo: 2.5<br>Ti: 9.5<br>O: 30.8<br>C: 50.0<br>Pt: 7.2	Mo^{4+}: 230.0 (18% [3]-30% [4])<br>Mo^{5+}: 231.1 (13% [3]-16% [4])<br>Mo^{6+}: 232.4 (69% [3]-54% [4])	458.7<br>TiO_2	530.0 metal oxide<br>531.2 -OH on metal oxides, CO_x: epoxide (31%)<br>533.2 carboxyls (6%)<br>535.9 moisture, satellite (3%)	284.4 graphitic C<br>286.2 C-O (2%)<br>288.6 C(O)-O (3%)	71.2 Pt^0<br>72.5 Pt^{2+}: PtO, $Pt(OH)_2$<br>75.0 Pt^{4+}: PtO_2
Pt/75GNP-S2	Mo: 2.1<br>Ti: 7.3<br>O: 24.9<br>C: 57.0<br>Pt: 8.7	Mo^{4+}: 229.9 (19% [3]-29% [4])<br>Mo^{5+}: 231.1 (11% [3] -11% [4])<br>Mo^{6+}: 232.5 (70% [3] -60% [4])	458.7<br>TiO_2	530.0 metal oxide<br>531.2 -OH on metal oxides, CO_x: epoxide (27%)<br>533.2 carboxyls (6%)<br>536.0 moisture, satellite (2%)	284.4 graphitic C<br>286.1 C- O (3%)<br>288.5 C(O)-O (1%)	71.2 Pt^0<br>72.6 Pt^{2+}: PtO, $Pt(OH)_2$
Pt/50GNP-S2	Mo: 1.9<br>Ti: 7.1<br>O: 28.7<br>C: 53.1<br>Pt: 9.2	Mo^{4+}: 230.0 (12% [3]-22% [4])<br>Mo^{5+}: 231.1 (16% [3] -16% [4])<br>Mo^{6+}: 232.5 (72% [3] -62% [4])	458.6<br>TiO_2	530.0 metal oxide<br>531.1 -OH on metal oxides, CO_x: epoxide (29%)<br>533.2 carboxyls (10%)<br>535.2 moisture, satellite (3%)	284.4 graphitic C<br>286.2 C-O (4%)<br>288.4 C(O)-O (3%)	71.3 Pt^0<br>72.6 Pt^{2+}: PtO, $Pt(OH)_2$<br>75.1 Pt^{4+}: PtO_2
Pt/25GNP-S2	Mo: 1.9<br>Ti: 7.0<br>O: 27.1<br>C: 57.0<br>Pt: 7.0	Mo^{4+}: 229.8 (17% [3]-26% [4])<br>Mo^{5+}: 231.1 (14% [3] -14% [4])<br>Mo^{6+}: 232.4 (69% [3] -60% [4])	458.6<br>TiO_2	530.0 metal oxide<br>531.1 -OH on metal oxides, CO_x: epoxide (26%)<br>533.1 carboxyls (13%)<br>535.7 moisture, satellite (3%)	284.4 graphitic C<br>286.1 C-O (6%)<br>288.5 C(O)-O (4%)	71.1 Pt^0

[1] nominal Mo/Ti ratio: 20:80; nominal mixed oxide/carbonaceous material ratio: 75:25; [2] BE: binding energy (in eV);
[3] at the first measurement; [4] after 3 h X-ray exposition.

Details of fitting of the XPS spectra of the samples were described in our previous publications (e.g., [1] and references cited therein), so they are not repeated here.

In general, the spectra and their general interpretation was very similar to those described in our previous works [1] and the differences between the samples with varying GNP/GO ratio were small.

In Figure S9 Mo 3d and Pt 4f spectra of the investigated electrocatalysts are shown. Apparently, the composition of the carbonaceous component of the support had no effect on the chemical states of its oxide component. The practically identical Mo 3d spectra (Fig. S9 A) revealed the presence of the Mo^{4+} (Mo $3d_{5/2}$ peak at 229.8-230.0 eV), Mo^{5+} (around 231.0 eV) and Mo^{6+} (at 232.4-232.5 eV) chemical states; the dominant contribution arose always from the Mo^{6+} state. Ti was exclusively in the Ti^{4+} ionic state in all samples.

Pt (Fig. S9 B) was predominantly metallic in all samples, irrespective to the composition of the support. The slight (not more than a few percents) ionic Pt signals can be attributed to slight oxidation upon storage in air.

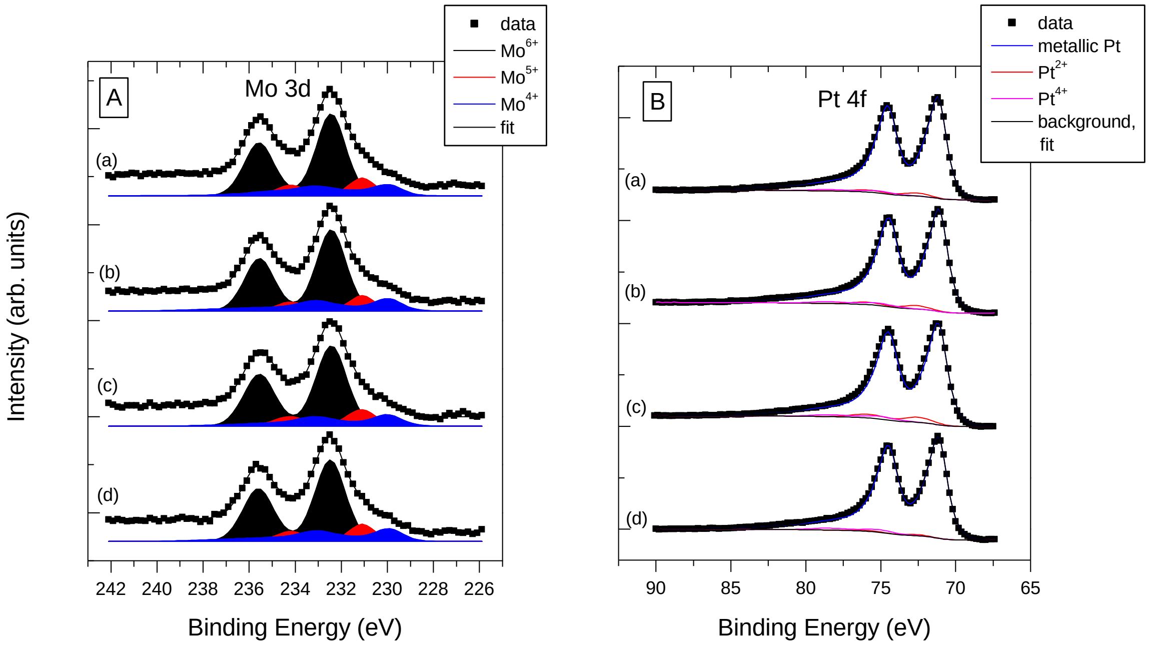

Figure S9. A: Mo 3d and B: Pt 4f core level spectra of (a): Pt/100GNP-S2; (b): Pt/75GNP-S2, (c): Pt/50GNP-S2; (d): Pt/25GNP-S2.

In line with the expectations, a slight effect of the changing GNP/GO ratio can be observed in the C 1s and O 1s spectra shown in Figure S10.

C 1s spectra of the electrocatalysts are presented in Fig. S10 A; for comparison, the spectrum of the parent GO material is also shown.

The GO spectrum (Fig. S10 A, curve (e)) exhibited the double-peaked envelope characteristic for graphite oxide materials [7]: a broad feature around 284.4 eV (due to graphene domains) was accompanied by a strong peak at 286.4 eV (C singly bound to O like in C-OH or C-O-C (epoxide, cyclic ether) groups). A weaker peak at 287.2 eV indicated the presence of carbonyl species in GO.

The C 1s spectrum of the electrocatalyst samples was dominated in all cases by the asymmetric line shape of the graphitic contributions (Fig. S10 A). Additional small peaks necessary for fitting the measured envelope were assigned to oxygen-containing functional groups (C singly bound to O slightly above 286 eV and carboxyl/lactone-like species around 288.5 eV, Table S7). Interestingly, a relatively strong carboxylic contribution (at 288.6 eV) was observed in the electrocatalyst with the purely GNP carbonaceous component. Nevertheless,

the predominantly graphitic nature of the carbon component of the catalysts on the GO-containing supports clearly indicated the significant transformation of the GO during support preparation, in agreement with the TG-MS results. Still, the amount of surface functional groups in these catalysts slightly increased with increasing nominal GO content.

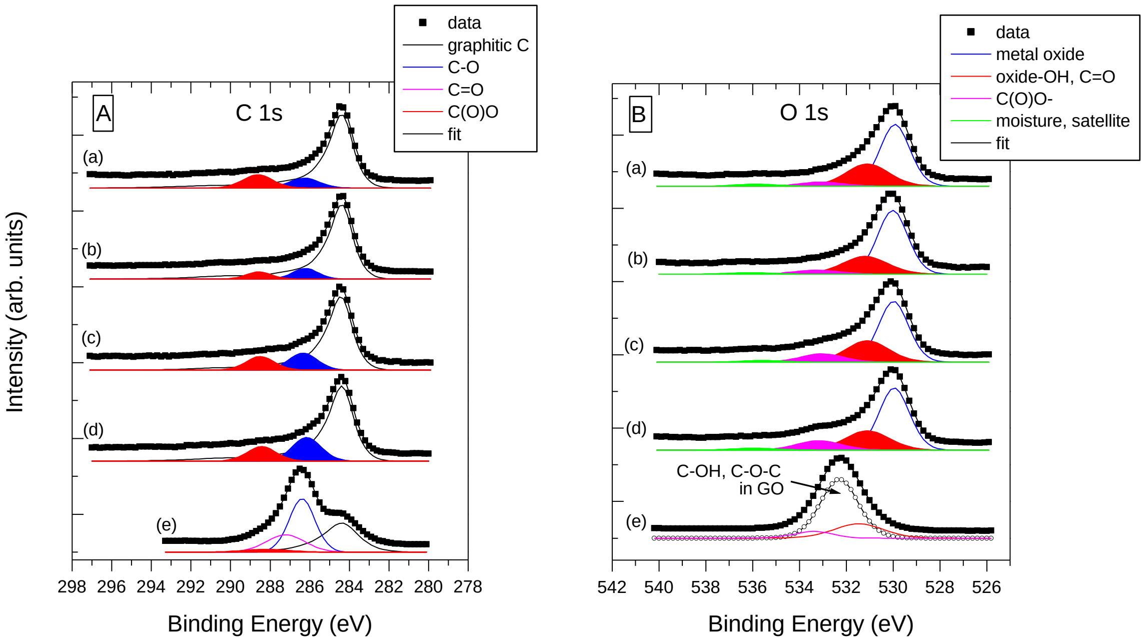

Figure S10. A: C 1s and B: O 1s core level spectra of (a): Pt/100GNP-S2; (b): Pt/75GNP-S2, (c): Pt/50GNP-S2; (d): Pt/25GNP-S2; and (e): GO prepared by Hummer's method.

O 1s spectra of the electrocatalyst samples, completed with the spectrum of the starting GO material, are shown in Fig. S10 B.

The GO spectrum (Fig. S10 B, curve (e)) corresponded to literature examples [7]: the main peak at 532.3 eV (arising from hydroxyl and epoxide groups of GO) was accompanied by smaller contributions at 531.2 eV (carbonyl species) and at 533.3 eV (mostly carboxyl groups).

The dominant contribution to the O 1s spectrum of the catalyst samples always arose from metal oxide species around 530.0 eV binding energy. In the spectrum of the catalyst prepared on the composite containing exclusively GNP (Pt/100GNP-S2), the metal oxide peak was accompanied by a broad, featureless band; it was tentatively decomposed into contributions from -OH groups on the metal oxide (overlapping with possible carbonyl signals) and further functional groups on the carbon. On the other hand, the high binding energy part of the O 1s spectrum of the GO-containing catalysts was more structured and decomposition into a range of different oxygen chemical states was possible (metal-oxide related hydroxyl groups and epoxide groups around 531 eV, carboxyl groups around 533 eV, Table S7). In particular, the peak around 533 eV, assigned to carboxylic species, clearly increased with increasing GO content. Nevertheless, the very significant decrease or even disappearance of the main GO-related peak evidenced once more the transformation of the GO material during the composite preparation.

XPS data for the carbonaceous component of the composite support demonstrate that even if the high temperature treatment effectively graphitizes the GO component, some of its functional groups are still preserved, which may be beneficial for stabilization of the oxide or the Pt component of the related electrocatalyst.

S3.2.4 TEM investigations

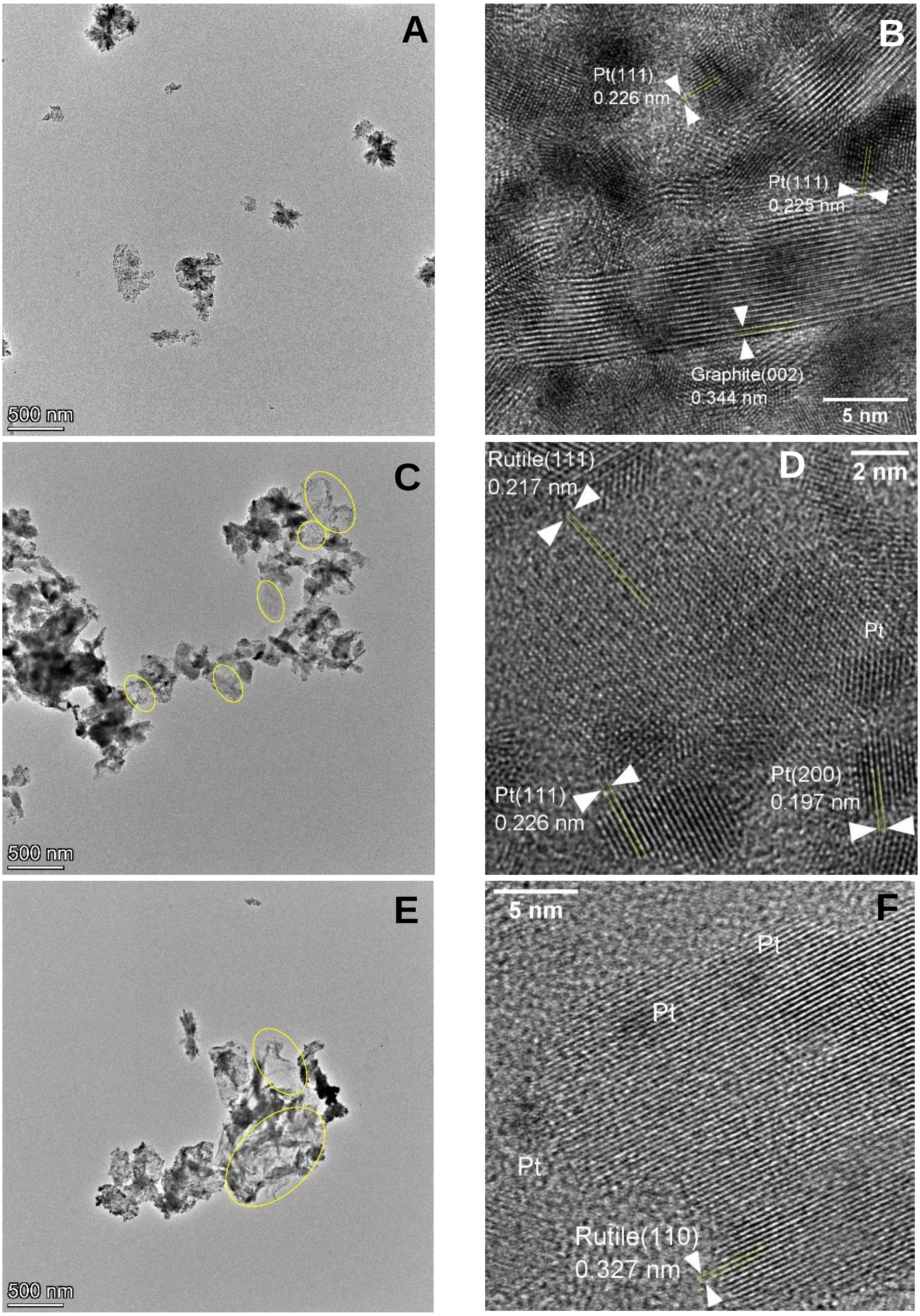

(continued on next page)

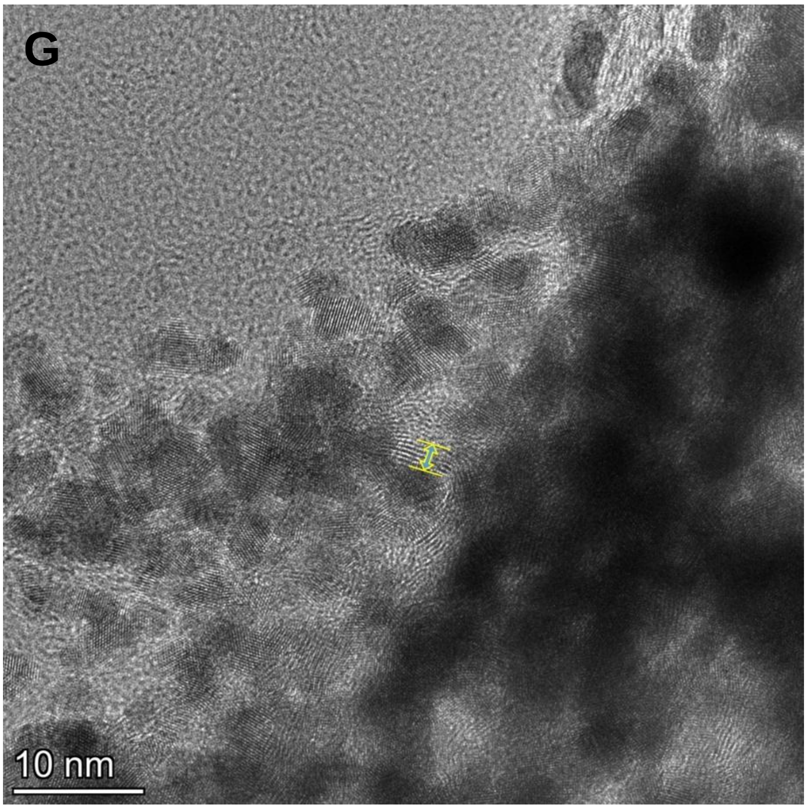

Figure S11. Transmission electron micrographs of Pt/100GNP-S2 (A, B), Pt/50GNP-S2 (C, D, G) and Pt/25GNP-S2 (E, F). Sheet-like objects are emphasized by yellow encircling in C and E. High resolution micrographs D and F were taken from the sheet-like regions. G: Fragment of the Pt/50GNP-S2 sample at high resolution. The yellow lines, separated by 2.055 nm, are laid over parallel atomic planes with 6 interlayers between the lines. The average interlayer distance is then 0.342 nm, providing clear indication of multilayered carbon particles.

S3.2.5 Elemental mapping

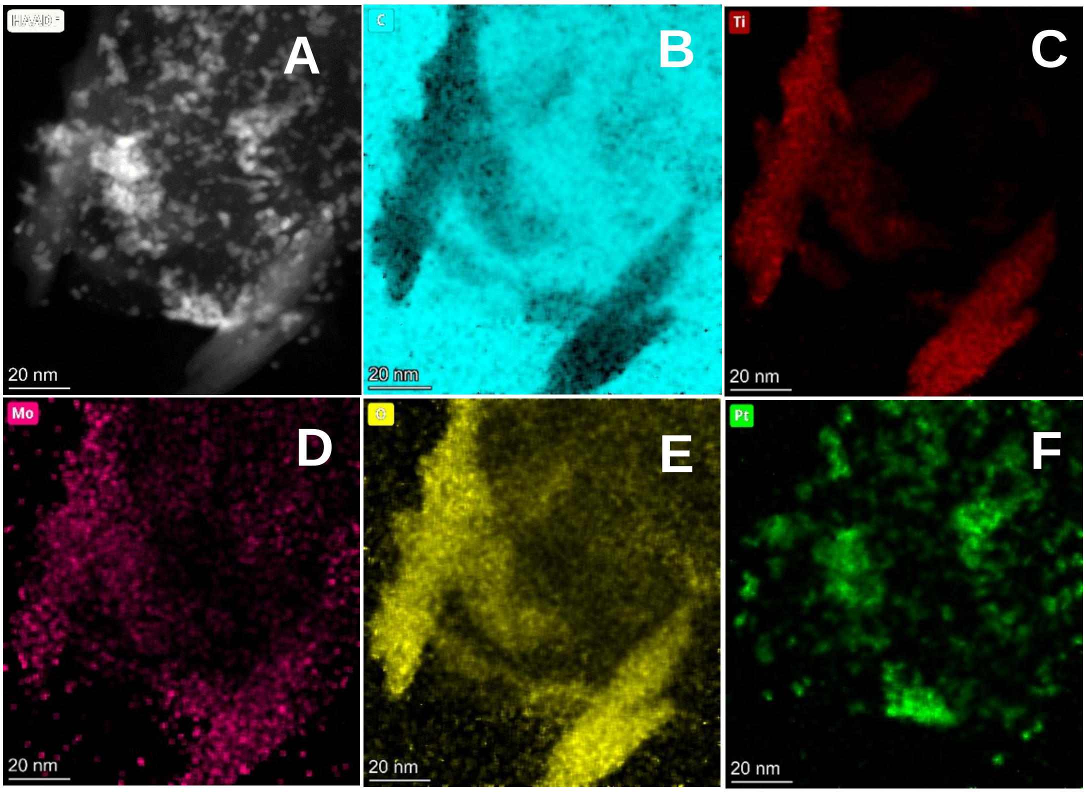

Figure S12. Elemental map of Pt/75GNP-S2 electrocatalyst. A: HAAD, B: C, C: Ti, D: Mo, E: O, F: Pt

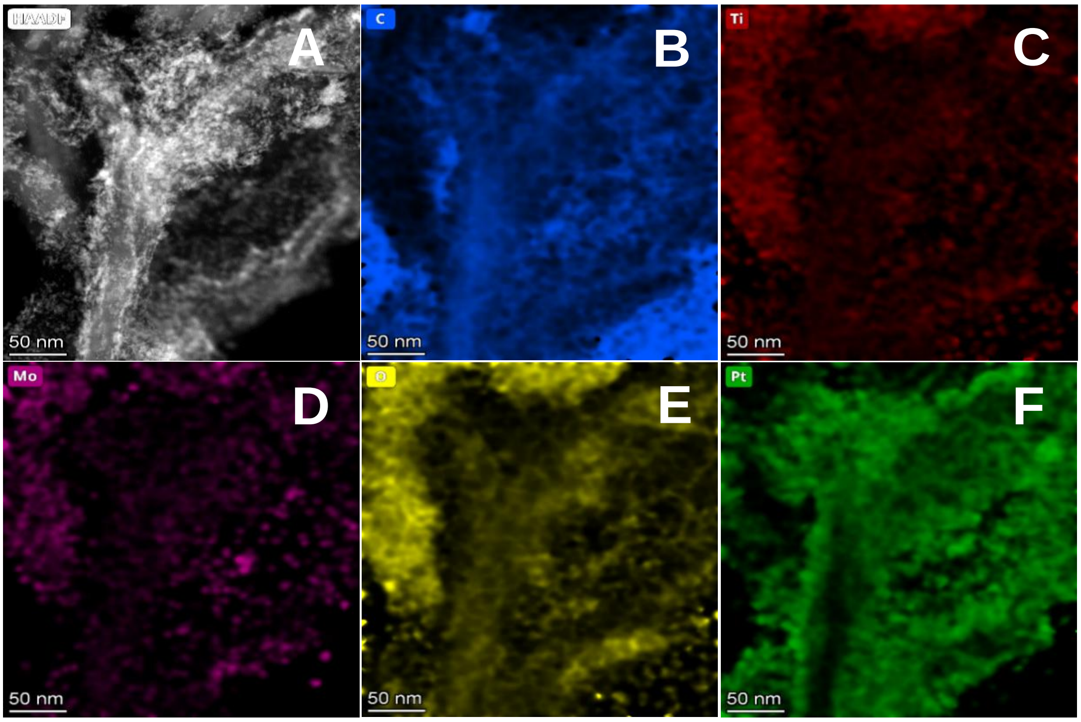

Figure S13. Elemental map of Pt/50GNP-S2 electrocatalyst. A: HAAD, B: C, C: Ti, D: Mo, E: O, F: Pt

S3.3. Electrochemical behavior of Pt/$Ti_{(1-x)}Mo_xO_2$-C composite type of catalyst derived from GNPs-GO mixtures with different GNPs/GO ratios

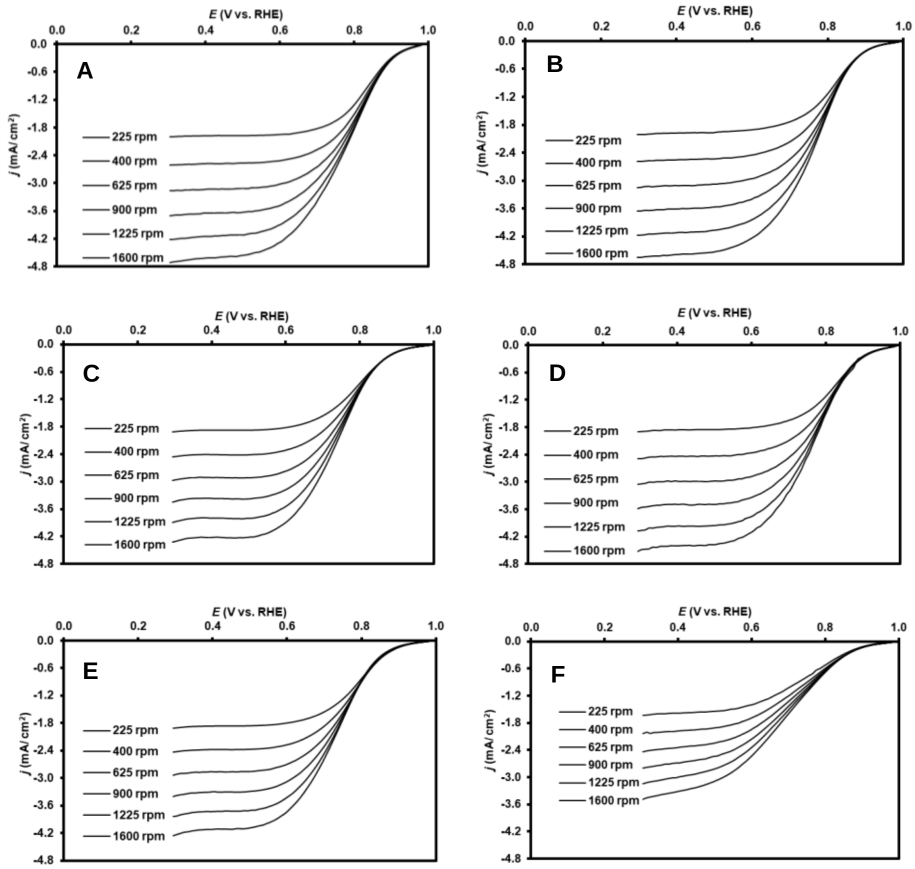

Figure S14. ORR polarization curves of the reference Pt/C (A) and Pt/$Ti_{0.8}Mo_{0.2}O_2$–C electrocatalysts: Pt/100GNP-S2 (B), Pt/75GNP-S2 (C), Pt/50GNP-S2 (D), Pt/25GNP-S2 (E) and Pt/100GO (F). *j* vs. *E* curves were recorded in O_2-saturated 0.5 M H_2SO_4 on a RDE at 225-1600 rpm. Sweep rate: 10 mV/s.

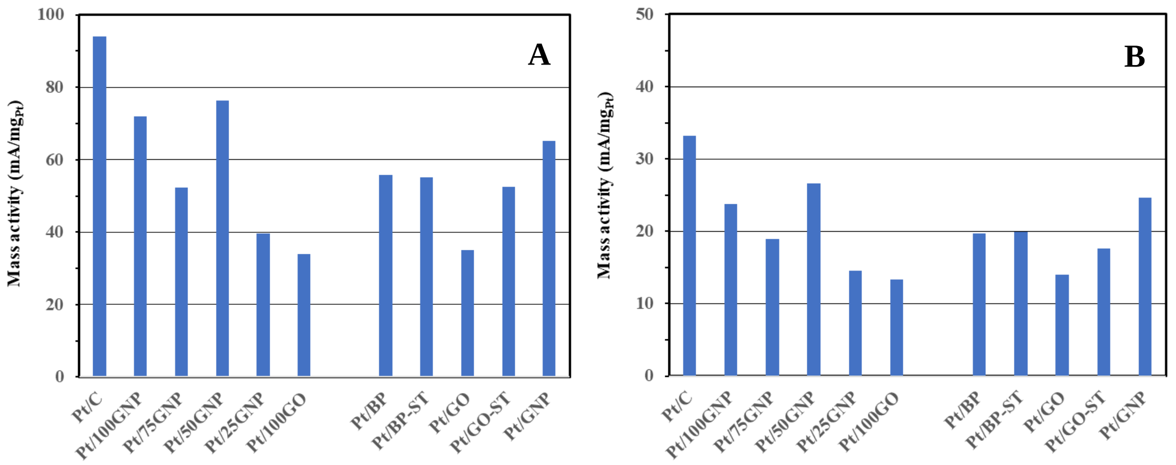

Figure S15. Mass activity obtained in the ORR on a RDE at 1600 rpm at 0.85 V (A) and 0.9 V (B) on the Pt/$Ti_{0.8}Mo_{0.2}O_2$–C electrocatalysts with $Ti_{0.8}Mo_{0.2}O_2$/C= 75/25 mass ratio presented in this work and in Ref. [1].

Table S8. Electrochemical performance of the reference 20 wt.% Pt/C (Quintech) and composite supported Pt catalysts: current density and mass activity obtained in the ORR at different potentials.

**Sample**	***j* @ 0.8 V, mA/cm^2** at 1600 rpm	***j* @ 0.8 V, mA/mg$_{Pt}$** at 1600 rpm	***j* @ 0.85 V, mA/mg$_{Pt}$** at 1600 rpm	***j* @ 0.9 V, mA/mg$_{Pt}$** at 1600 rpm	**Ref.**
**$Ti_{0.8}Mo_{0.2}O_2$/C=75/25**					This work
**Pt/100GO**	0.68	70.1	34.0	13.33	
**Pt/25GNP-S2**	0.88	98.7	39.7	14.6	
**Pt/50GNP-S2**	1.51	159.6	76.4	26.6	
**Pt/75GNP-S2**	1.00	123.6	52.4	19.0	
**Pt/100GNP-S2**	1.53	169.9	72.0	23.8	
**Pt/C**	1.84	180.9	94.0	33.2	
**$Ti_{0.8}Mo_{0.2}O_2$/C=75/25**					[1]
**Pt/BP**[1]	1.16	113.8	55.8	19.7	
**Pt/BP-ST**[2]	1.06	104.1	55.2	19.9	
**Pt/GO**	0.72	70.7	35.0	14.0	
**Pt/GO-ST**	1.23	121.0	52.5	17.6	
**Pt/GNP-NG**[3]	1.48	145.3	65.3	24.6	
**$Ti_{0.8}Mo_{0.2}O_2$/C=25/75**					[8]
**Pt/FBP**[4]	0.94	91.9	51.8	20.6	
**Pt/BP**	1.29	127.1	67.5	24.1	
**Pt/V**[5]	1.36	133.0	75.0	28.9	
**$Ti_{0.8}Mo_{0.2}O_2$/FBP=50/50**					[9]
**Pt/FBP**[4]	1.30	127.6	67.7	27.1	
**Pt/FBP-150H**[6]	1.64	161.3	80.8	29.5	
**Pt/FBP-250H**[6]	2.02	197.9	101.3	39.4	
**Pt/FBP-350H**[6]	1.79	176.1	89.5	34.1	
**Pt/FBP-450H**[6]	1.89	185.9	93.8	33.9	
**Pt/C**	1.94	190.0	101.9	37.2	

[1] BP: Black Pearls 2000;
[2] ST: solvothermal treatment was inserted before the HTT step (for details see ref. [1]);
[3] GNP-NG: Nanographi;
[4] FBP: functionalized BP;
[5] V: Vulcan XC-72;
[6] Pt/FBP reduced in H_2 in the 150-450°C temperature range.

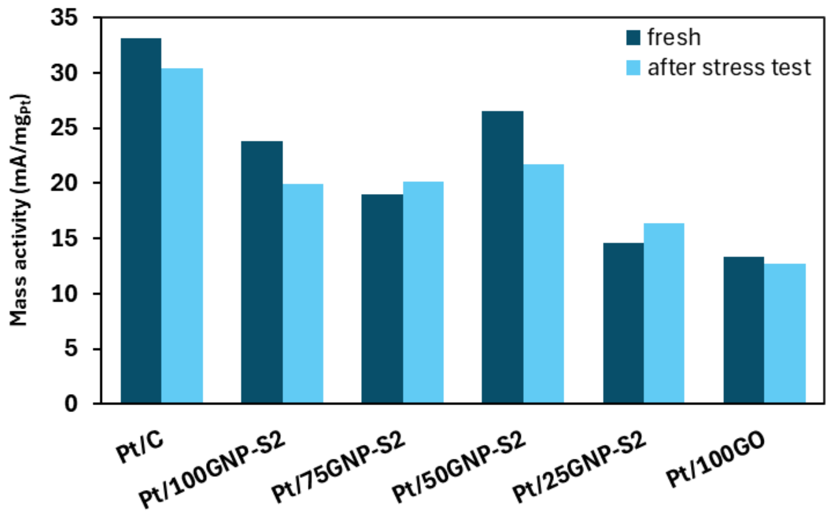

Figure S16. The mass activities determined at 0.9 V before and after 500 polarization cycles of the synthesized catalysts and the commercial Pt/C evaluated at 1600 rpm rotation speed.

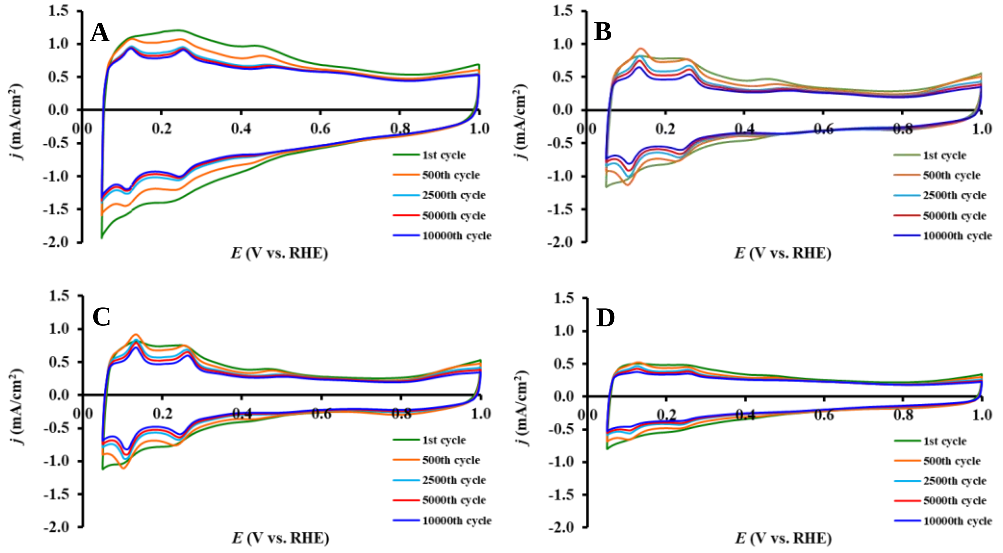

Figure S17. Cyclic voltammograms of the Pt/100GO (A), Pt/100GNP-S2 (B), Pt/25GNP-S2 (C) and Pt/75GNP-S2 (D) electrocatalysts obtained during 10,000-cycle stability test. Recorded in 0.5 M H_2SO_4 solution with 100 mV/s sweep rate, T= 25 °C.

References:

1. Ayyubov, I.; Tálas, E.; Borbáth, I.; Pászti, Z.; Silva, C.; Szegedi, Á.; Kuncser, A.; Yazici, M.S.; Sajó, I.E.; Szabó, T.; et al. Composites of Titanium–Molybdenum Mixed Oxides and Non-Traditional Carbon Materials: Innovative Supports for Platinum Electrocatalysts for Polymer Electrolyte Membrane Fuel Cells. *Nanomaterials* **2024**, *14*, doi:10.3390/nano14121053.
2. Ayyubov, I.; Borbáth, I.; Pászti, Z.; Sebestyén, Z.; Mihály, J.; Szabó, T.; Illés, E.; Domján, A.; Florea, M.; Radu, D.; et al. Synthesis and Characterization of Graphite Oxide Derived TiO_2-Carbon Composites as Potential Electrocatalyst Supports. *Top. Catal.* **2021**, doi:10.1007/s11244-021-01513-1.
3. Vass, Á.; Borbáth, I.; Bakos, I.; Pászti, Z.; Sáfrán, G.; Tompos, A. Stability Issues of CO Tolerant Pt-Based Electrocatalysts for Polymer Electrolyte Membrane Fuel Cells: Comparison of Pt/$Ti_{0.8}Mo_{0.2}O_2$–C with PtRu/C. *React. Kinet. Mech. Catal.* **2019**, *126*, 679–699, doi:10.1007/s11144-018-1512-z.
4. Vass, Á.; Borbáth, I.; Pászti, Z.; Bakos, I.; Sajó, I.E.; Németh, P.; Tompos, A. Effect of Mo Incorporation on the Electrocatalytic Performance of Ti–Mo Mixed Oxide–Carbon Composite Supported Pt Electrocatalysts. *React. Kinet. Mech. Catal.* **2017**, *121*, 141–160, doi:10.1007/s11144-017-1155-5.
5. Woods, R. *Electroanalytical Chemistry: A Series of Advances*; Bard, A.J., Ed.; M. Dekker: New York; Basel, 1976; Vol. 9, pp. 1–162;.
6. Schulenburg, H.; Durst, J.; Müller, E.; Wokaun, A.; Scherer, G.G. Real Surface Area Measurements of Pt_3Co/C Catalysts. *J. Electroanal. Chem.* **2010**, *642*, 52–60, doi:10.1016/j.jelechem.2010.02.005.
7. Yamada, Y.; Yasuda, H.; Murota, K.; Nakamura, M.; Sodesawa, T.; Sato, S. Analysis of Heat-Treated Graphite Oxide by X-Ray Photoelectron Spectroscopy. *J. Mater. Sci.* **2013**, *48*, 8171–8198, doi:10.1007/s10853-013-7630-0.
8. Ayyubov, I.; Tálas, E.; Salmanzade, K.; Kuncser, A.; Pászti, Z.; Neaţu, Ș.; Mirea, A.G.; Florea, M.; Tompos, A.; Borbáth, I. Electrocatalytic Properties of Mixed-Oxide-Containing Composite-Supported Platinum for Polymer Electrolyte Membrane (PEM) Fuel Cells. *Materials (Basel).* **2022**, *15*, 3671, doi:10.3390/ma15103671.
9. Silva, C.; Borbáth, I.; Dodony, E.; Olasz, D.; Sáfrán, G.; Szegedi, Á.; Zelenka, K.; Tompos, A.; Pászti, Z. Stability Enhancement of Molybdenum Modified Rutile – Carbon Composite Supported Platinum Electrocatalysts by Reductive Pretreatment: Surface Characteristics and Advanced Electrocatalytic Properties. *Mater. Res. Bull.* **2025**, *182*, doi:10.1016/j.materresbull.2024.113114.
